# Comparing patterns of intergenerational class mobility using log-linear models: evidence from seven countries, two cohorts, and gendered stratification

**DOI:** 10.3389/fsoc.2026.1757240

**Published:** 2026-05-01

**Authors:** César Augusto Ricardi Morgavi

**Affiliations:** Department of Social and Legal Science, CUCEA, University of Guadalajara, Zapopan, Mexico

**Keywords:** birth cohorts, comparative sociology, gender inequality, intergenerational social class mobility, Latin America and Europe, log-linear models, Mexico, social fluidity

## Abstract

Intergenerational class mobility remains a central indicator of social openness and stratification dynamics. Comparative analyses increasingly show that mobility patterns vary across national contexts, welfare regimes, birth cohorts, and gender structures. However, few studies simultaneously assess these dimensions using harmonized class schemas and modeling frameworks across multiple countries and controlling multiple layers in loglinear models. This study examines intergenerational class mobility using harmonized mobility tables linking respondents' social class of origin and social class of destination across seven countries: Mexico, Chile, Uruguay, Spain, Sweden, the United Kingdom, and Germany. The analysis includes two birth cohorts (1930–1975 and 1976–1990) and gender. A sequence of log-linear models was estimated, including a conditional independence model as the baseline model, constant social fluidity (homogeneous association) models, and uniform difference models to assess whether, and to what extent, the association between origin and destination varies across country, cohort, and gender layers. Baseline models showed poor fit, indicating strong intergenerational class persistence. Constant social fluidity models substantially improved fit, suggesting stable structures of association. Uniform difference estimates revealed patterned variability across countries, with Mexico and Chile showing stronger class inheritance, and Sweden and the United Kingdom displaying comparably weaker associations. Gender and cohort differences were present, though less pronounced and not uniform across countries. Findings provide comparative evidence of both stability and structured variation in mobility patterns across national contexts. The results highlight the importance of jointly analyzing countries, cohorts, and gender when evaluating class mobility regimes.

## Introduction

Intergenerational class mobility provides a key lens for understanding the extent to which social origins influence individual life chances and the degree to which societies reproduce or overcome inequality across generations.

Comparative research has increasingly demonstrated that countries differ significantly in the extent of relative social mobility, reflecting differences in institutional designs, welfare regimes, educational systems, and labor market segmentation. These differences may also manifest across birth cohorts, as institutional reforms, demographic shifts, and economic restructuring alter the pathways linking class origins to adult outcomes. Likewise, gender may constitute a relevant axis of variation, as women's expanded participation in education and employment has transformed stratification patterns, although the degree and timing of change varies across national contexts.

Despite the growing literature, relatively few studies examine the joint variation of class mobility across multiple countries, cohorts, and gender within a harmonized analytical framework using comparable class schemas and statistical models. This type of design is essential for assessing whether mobility patterns are stable or dynamic, convergent or divergent, and gender-neutral or gendered. Moreover, distinguishing stability from structured variation requires statistical models capable of testing whether the strength of association between origin and destination is constant across contexts or differs in patterned ways.

This study contributes to the comparative analysis of class mobility by examining harmonized five-category origin (O) and destination (D) class schemas across seven countries: Mexico, Chile, Uruguay, Spain, Sweden, the United Kingdom, and Germany. Two birth cohorts are included to capture temporal change, and analyses are disaggregated by gender to observe possible differentiation in stratification processes. Using a series of log-linear models, the analysis evaluates whether the association between origin and destination is constant across countries, cohorts, and gender, or whether patterned variation exists.

Three guiding research questions motivate the analysis: (1) to what extent do patterns of intergenerational class relative mobility (social fluidity) differ across national contexts? (2) Does the strength or structure of class relative mobility change across birth cohorts? (3) Are mobility patterns gendered, and if so, does this variation depend on the national context or historical period? By addressing these questions with harmonized data and a consistent modeling strategy, the study seeks to provide empirical clarity on whether mobility regimes exhibit stability, change, or structured heterogeneity across countries, time, gender, and welfare regimes.

### The debate among theoretical perspectives

Research grounded in classical modernization theory has long argued that processes of industrialization and educational expansion should weaken the influence of ascribed attributes—including parental class—while widening opportunities for attainment through meritocratic mechanisms. From this perspective, mobility increases across cohorts and societies converge toward higher levels of openness as institutional development and human capital investments expand. In contrast, reproduction-based perspectives maintain that class structures and institutional arrangements—rather than modernization alone—shape how mobility dynamics unfold, often preserving advantages for privileged groups even amid educational expansion and economic growth. More recent contributions have emphasized the interplay of individual strategies, institutional sorting mechanisms, and demographic change, suggesting that mobility patterns are shaped neither exclusively by structural constraints nor by universal modernization trends, but by contextual configurations that vary across time, space, and social groups.

Parsons viewed modernization as an evolutionary process in which achievement replaces ascription as the dominant principle of stratification, and where meritocratic allocation mechanisms gradually weaken the role of parental origin (Parsons, 1940). Similarly, Treiman (1970) formulated the thesis of industrialization and stratification, arguing that industrial societies should converge toward similar mobility patterns over time, driven by technological development, expanding educational systems, and rational-bureaucratic labor markets. Duncan's (1961, 1966) status attainment research further reinforced this logic, demonstrating that as educational attainment expands, it mediates and weakens the direct effect of class origin on occupational destinations.

From this perspective, modernization should produce three empirical consequences: (a) increasing absolute mobility as industrial labor markets expand; (b) declining effects of social origin on class destinations; and (c) convergence across societies in mobility patterns as industrialization proceeds (Ganzeboom et al., 1989). This expectation aligns with the first and second research questions insofar as modernization theory anticipates both cross-national convergence and cohort-based weakening of origin–destination associations, implying rising social fluidity.

However, strong critiques emerged questioning the teleological assumptions embedded in modernization theory. For reproduction theorists, led by Bourdieu and Passeron (1970), industrial societies do not gradually converge toward openness; instead, their institutions—particularly schools—act as mechanisms that legitimize and reproduce class inequalities. Education is neither neutral nor meritocratic but is embedded in symbolic hierarchies that privilege the dominant classes. Reproduction theorists argue that schooling functions as an ideological instrument in service of the dominant class reinforcing the unequal distribution of cultural capital and creating differential probabilities of educational and occupational success (Bourdieu and Passeron, 1970; Bernstein, 1971).

From this standpoint, the school-to-labor-market pipeline mirrors class privilege rather than disrupting it, producing persistent opportunity inequalities for social class mobility across generations. In this view, the expectation is not convergence but structural persistence, where the association between class origin and destination remains strong, regardless of economic growth or educational expansion. Therefore, the theory anticipates limited variation across cohorts and national contexts.

Between these approaches lies the influential hypothesis by Featherman, Jones, and Hauser (FJH), which affirms that industrial societies share a “genotypical” pattern of social fluidity that is broadly similar across contexts once structural occupational distributions are controlled (Featherman et al., 1978). FJH differentiate between phenotypical mobility—observable differences resulting from structural shifts—and genotypical mobility, conceptualized as the underlying relational pattern of origin–destination association, which they argue remains stable across industrial societies (Featherman et al., 1978). This formulation allowed scholars to reconcile observed differences in mobility levels with theoretical expectations of structural similarity among developed countries. This hypothesis was later challenged: Ganzeboom et al. (1989) and Sørensen (1992) provided empirical evidence rejecting full convergence, demonstrating statistically significant cross-national variation in mobility patterns across industrial societies, even after accounting for structural effects.

It is within this debate that the work of Erikson and Goldthorpe (1992) emerges as a methodological and conceptual turning point. Their thesis of constant social fluidity (CnSF) tests both modernization expectations of convergence and reproductionist claims of persistent inequality. Using harmonized data and log-linear modeling across twelve countries, they demonstrated that while occupational structural change generates rising absolute mobility, the underlying pattern of relative mobility—the extent to which origin predicts destination—is remarkably stable over time and across societies (Erikson and Goldthorpe, 1992). This insight aligns directly with the modeling strategy used in this study, where the CnSF model acts as a theoretical benchmark for comparing variation in the OD association across gender, cohorts, and institutional contexts.

However, the claim of universal stability has also been problematized by comparative welfare regime research. Esping-Andersen's framework of welfare state typologies—liberal, conservative-corporatist social democratic and, later added, familist-Mediterranean—argues that mobility dynamics are embedded in national institutional arrangements and redistributive policies, generating distinct mobility regimes (Esping-Andersen, 1990, 1999, 2015). From this perspective, rather than converging, societies differentiate along institutional trajectories shaped by labor markets, family structures, and state intervention. These patterns imply that the extent, direction, and gendering of mobility—directly relevant to the third research question—depend on welfare institutions and their alignment with opportunity structures.

Recent Latin American and European mobility studies have reinforced this view, emphasizing structural heterogeneity across national and regional contexts in terms of social fluidity—contrary to what happens with absolute mobility rates (Solís and Boado, 2016; Shavit and Blossfeld, 1993; Filgueira, 2013). The evidence reviewed further indicates that scholars now treat mobility regimes as historically and institutionally contingent rather than universally convergent.

Taken together, these theoretical perspectives offer contrasting expectations relevant to interpreting the findings of this study. To sum up:

- Modernization theory predicts rising and converging social fluidity across countries and cohorts, with declining relevance of class origin and minimal gender differentiation.
- Reproduction theories predict persistent structural inequalities and limited cohort change.
- FJH hypothesis predicts broad cross-national similarity once structural change is controlled.
- Erikson and Goldthorpe's thesis predicts stability in relative mobility despite structural shifts.
- Welfare regime approaches predict structured heterogeneity across national, temporal, and institutional contexts— including gendered variation.

Recent comparative research has renewed the traditional debate between modernization theory and the constant social fluidity hypothesis by demonstrating that mobility trajectories depend critically on institutional configurations and the interaction between welfare regimes, labor markets and educational systems. Contemporary analyses such as Martín-Artiles et al. (2021) shows that coordinated economies in Northern Europe combine strong pre-distribution mechanisms—like collective bargaining and wage coordination—with robust post-distribution policies, thereby generating structurally more equal opportunity regimes than the liberal or structurally heterogeneous systems typical of Latin America. These institutional arrangements provide a more nuanced framework for selecting country cases, aligned with Esping-Andersen's classical typology. Adopting a multidimensional perspective on social models or welfare regimes offers a theoretically grounded criterion for grouping countries in mobility research when comparing Europe and Latin America.

At the same time, the empirical research on mobility for Latin America regimes has advanced significantly, offering an updated basis for evaluating whether industrialization and educational expansion have increased merit-based mobility or not. One of the most recent cohort analyses for Argentina, Chile, and Uruguay (Jorrat et al., 2024) reveals that education has not uniformly worked as an equalizing force—and that improvements in relative mobility are far from generalizable across countries or genders. The comparative analysis across 26 European nations by Barone and Ruggera (2018) demonstrates that, despite widespread educational expansion, inequality of educational opportunity persisted across most of them in the postwar period. The heterogeneity across these 26 nations underscores the importance of institutional and policy contexts, challenging narratives of universalistic linear progress in mobility.

Similarly, studies of Spain and Chile show that while education remains a key determinant of mobility, gendered patterns of reproduction persist over time, reinforcing the need to integrate gender into debates on stratification and fluidity (Segura-Carrillo, 2025). These findings align with broader evidence from comparative Europe–Latin America research demonstrating that schooling is a powerful mediator of class mobility but only a limited equalizer (Solís and Dalle, 2019; Fachelli et al., 2021). These body of recent analysis underscores the importance of incorporating structural heterogeneity, gender, and institutional diversity into models, moving beyond simplistic modernization-based assumptions and toward a more empirical and comparative understanding of social fluidity patterns.

### Institutional development, welfare regimes, and labor market entry across periods

Understanding differences in intergenerational class mobility requires situating each birth cohort within the political economy, institutional structures, and welfare regimes shaping their transition into adulthood. The two cohorts analyzed—those born between 1930 and 1975 (entering adulthood approximately 1955–1995) and those born between 1976 and 1990 (entering adulthood approximately 1996–2011)—experienced markedly different configurations of industrial development, state intervention, educational expansion, gender occupational participation, and welfare state maturity.

For the older cohort (1930–1975 births), entry into the labor market occurred in a period characterized by the consolidation of Fordist production systems, the expansion of the bureaucratic-industrial state, the institutionalization of welfare systems designed to regulate class inequalities and stabilize labor markets. In the European cases—particularly Sweden, Germany, and the United Kingdom—this period reflected the height of postwar welfare settlement: publicly financed education systems expanded, vocational pathways formalized, and employment security regimes regulated hiring and dismissal practices. This historical phase produced the alignment between industrialization, corporatist regulation, and mass education, generating expectations of upward mobility and greater merit-based allocation of occupational positions. This cohort matured under relatively solidified welfare arrangements: social democratic in Sweden, conservative-corporatist in Germany, and liberal in the United Kingdom—each with different implications for the openness of class transitions and the gendered distribution of opportunity (Esping-Andersen, 1999; Hega and Hokenmaier, 2002; Moreno et al., 2014; Ricardi-Morgavi, 2020).

In contrast, the same period in Latin America—particularly Chile, Mexico, and Uruguay—followed a distinct institutional trajectory. These societies developed under import-substitution industrialization (ISI) models marked by state-led industrialization, segmented labor markets, and corporatist political structures. Welfare arrangements in this period were characteristically fragmented—generous for formal workers but absent or residual for informal labor sectors, producing structurally dualistic opportunity regimes. Although educational systems expanded and occupational structures diversified, the stratification system remained strongly influenced by ascription—family class background, gender, and region of birth—limiting fluidity at the top and bottom of the structure. This resulted in rigidity in access to elite positions and strong reproduction among working-class strata across these national cases. Women in this cohort, across both world regions, entered adulthood under gender regimes still heavily shaped by traditional male-breadwinner models, limiting their participation in the occupational hierarchy even where welfare states were progressive in redistributive policy.

The historical context of the younger cohort (1976–1990) diverges sharply. Their transition to adulthood—between 1996 and 2011—occurred amid economic globalization, technological restructuring, labor flexibilization, and welfare transformation. For Europe, this period reflects the shift toward post-industrial economies, marked by the decline of manufacturing, expansion of service employment, privatization trends, and the weakening of employment protection. What Esping-Andersen (1999) terms “the transition to post-industrial welfare states” brought new social risks—precarity, unemployment, and credential inflation—especially affecting youth and women. Educational access expanded, however, the relationship between education and occupational attainment became less linear as credential competition intensified.

In Latin America, the same period saw the dismantling of ISI models and the implementation of neoliberal reforms, including privatization, trade liberalization, labor deregulation, and restructuring of social protection—often toward means-tested poverty relief rather than universal insurance. This shift widened inequalities between formal and informal labor market participants and generated what multiple analyses describe as “dual or residual welfare regimes with stratified access to mobility opportunities” (Ricardi-Morgavi, 2020, p. 125–146). Unlike the older cohort, the younger cohort entered adulthood under conditions of greater educational attainment but weaker institutional guarantees linking credentials to mobility.

Across both regions, gender dynamics transformed significantly for the younger cohort: women's labor force participation expanded, legal frameworks strengthened anti-discrimination norms, and educational attainment often surpassed that of men. However, segmentation persists—horizontal in Europe and both horizontal and vertical in Latin America—meaning that gains in educational parity did not necessarily translate to comparable gains in relative mobility.

Considered as a whole, the institutional, economic, and welfare configurations of each period suggest theoretically meaningful expectations concerning the study's guiding questions: differences across national contexts should reflect regime structures rather than convergence; cohort dynamics may reveal either stability—as suggested by Erikson and Goldthorpe (1992)—or increasing heterogeneity as predicted by welfare-regime and globalization theoretical perspectives; and gendered mobility patterns should be more visible in the younger cohort where women's participation expanded but stratification barriers persisted.

**Figure 1:**
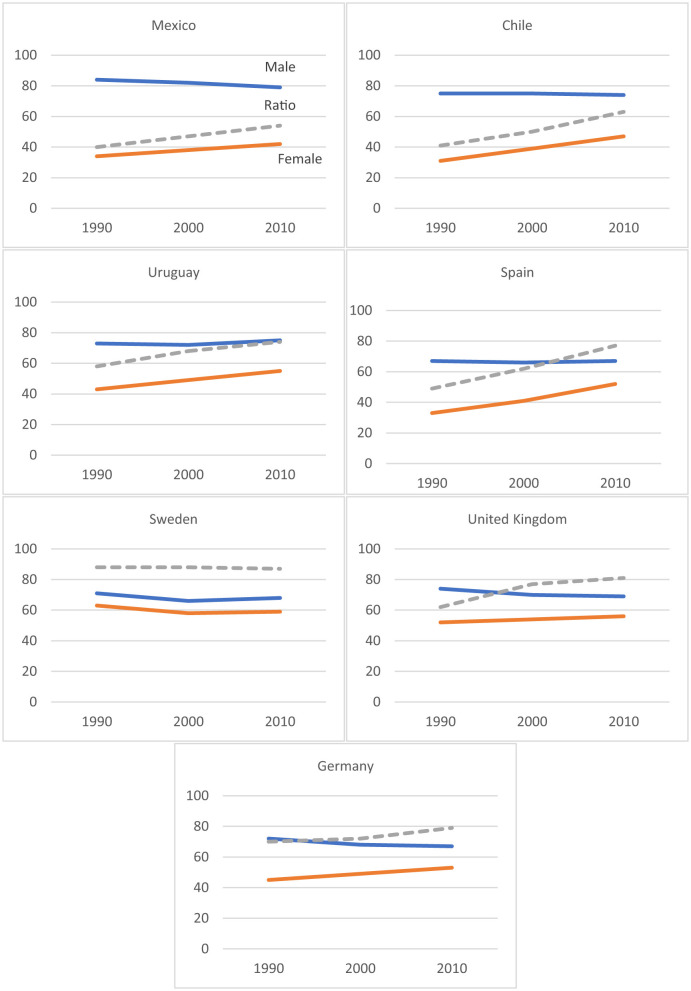
Male and female labor force participation rates and ratio of female to male labor force participation rate (% of total population ages 15+). Source: International Labour Organization, 2026.

In Mexico and Chile, male participation rates remain high and relatively stable over time, while female participation increases gradually, generating a slow but steady rise in the female-to-male participation ratio (Figure 1). This pattern contrasts with Uruguay and Spain, where the gender gap narrows more decisively due to both rising female participation and stagnation in male rates. Spain exhibits one of the most pronounced upward trends in the ratio, signaling a substantial long-term convergence between men and women participation.

In the high-income European countries—Sweden, the United Kingdom, and Germany—the gender gap is considerably smaller from the outset. Sweden displays near parity but with stagnation over time, reflecting a mature gender-egalitarian labor market with limited margin for further equalization. The United Kingdom shows convergence driven primarily by rising female participation, while Germany combines moderate increases in female participation with slight declines in male rates, resulting in a gradual improvement in the ratio. Overall, the comparative evidence suggests that while Latin American countries continue to experience incremental and heterogeneous gains, high-income European cases demonstrate either sustained parity (Sweden) or steady movement toward it (UK and Germany).

The trends depicted across countries suggest that changes in women's labor force participation are tightly linked to their potential for social mobility over time. In contexts such as Spain, the United Kingdom, Uruguay, and to a lesser extent Mexico and Chile, the steady rise in female participation expands women's access to mechanisms of social mobility and intergenerational transmission of human capital. By contrast, in countries like Sweden, where parity was achieved earlier and participation rates remain stable, social mobility is shaped less by labor market entry. Meanwhile, where female participation grows modestly relative to men, as in Germany, the capacity of women to convert labor market presence into substantive mobility may be constrained. Overall, it is prospected that mobility tend to improve where gender gaps shrink and female labor market participation rises, remain constant where parity has been institutionalized, and risk stagnation where gendered participation patterns change slowly or plateau.

## Materials and methods

### Data and sample design

The analysis draws on harmonized cross-national survey microdata from each country. The analytic sample includes men and women and treats gender as an axis of stratification rather than solely a demographic control. Individuals were included if valid information was available for their occupational class and the occupational class of at least one parent during adolescence. Respondents were grouped into two birth cohorts (1930–1975 and 1976–1990) to assess temporal variation in class mobility structures. Individuals with missing information on any key variable were excluded from the analysis to maintain consistent model estimation across all subgroup comparisons.

This research uses representative surveys of the economically active population of both sexes aged 20 and over, with a statistically significant sample size in relation to the total population, a confidence level of 95% and a sampling error equal to or less than ±5%. For Mexico, the subsamples were obtained from the 2011 ESRU Social Mobility Survey (ESRU-EMOVI; *N* = 5,981); for Chile, from the 2009 National Social Stratification Survey (ENES; n = 2,831); for Uruguay, from the 2010 Occupational Mobility and Educational Trajectories Survey (EMOTE; *n* = 2021). For European countries, the subsamples come from the European Social Survey European Research Infrastructure (ESS ERIC) (2025); Spain *n* = 1,396, Sweden *n* = 1,236, United Kingdom *n* = 1,211 and Germany *n* = 2,241.

### Measurement of class position

Intergenerational class mobility was operationalized using a harmonized five-category version of the Erikson–Goldthorpe–Portocarero (EGP) class schema. This reduction balances conceptual comparability and statistical feasibility across countries and subpopulations. The categories used were: (1) service class I + II; (2) routine non-manual occupations I + III; (3) petty bourgeoisie in European countries and self-employed traders in Latin American ones (Iva + b) (see Solís and Boado, 2016); (4) skilled manual and technical occupations; (5) semi-skilled and unskilled manual labor; (6) farm owners; and (7) agricultural workers. Respondents' class of origin was measured using the parental occupation during interviewee's adolescence, and class of destination was based on their current or last main occupation of the interviewee. These seven categories were grouped and reordered to optimize calculations given the sample sizes, resulting in the following (Table 1).

**Table 1:** Adapted EGP social class classification.

EGP European countries (Spain, Sweden, the UK, and Germany)	EGP Latin American countries (Mexico, Chile, and Uruguay)
I + II Service class	I + II Service class
IIIa + b routine non-manual occupations	IIIa + b routine non-manual occupations
IVabc petty bourgeoisie + farm owners	IVab self-employed traders
V + VI skilled manual and technical occupations	V + VI skilled manual and technical occupations
VIIab semi and unskilled manual + agricultural workers	IVc+VIIab semi and unskilled manual + farm employer and workers

Class IVc was placed at the base of the structure of Latina American countries after confirming that it has a lower hierarchy than its European counterpart in terms of economic income, assets, and education. Following the proposal of (Solís and Boado 2016), with which I agree, this position is the most appropriate for classifying Latin American cases under the EGP scheme.

### Stratification dimensions

Three additional stratifying variables were incorporated to examine contextual variation in the association between social origin and destination. Country (seven categories), represents cross-national diversity in institutional arrangements and welfare regimes. Birth cohort (two categories), captures possible historical change in stratification patterns. Gender (two categories), allows assessment of gendered mobility structures. These dimensions were used in multiple model specifications to test whether mobility patterns were constant or variable across contexts.

The social origin is based on the convention of the father's social class at the age of 15 of the respondents. The fathers (males) are used as the reference point for both male and female respondents (daughters). While this does raise the issue of occupational segregation by gender in intergenerational comparisons, it was not feasible to use mothers as a reference point, since a significant number of them were outside the labor market, especially in Latin American countries.

Furthermore, it maintains the same reference point for origins for both sexes, which allows to compare the results for sons and daughters, as there are doubts about whether it is more appropriate to use information from the father, the mother, or both, as the reference point for daughters' origins (Breen, 2004; Solís and Boado, 2016). Therefore, it is appropriate to maintain the comparison with the interviewees' fathers, since they participated in the labor market to a greater extent, providing information about their social class.

### Analytical strategy

Log-linear models were estimated to assess the strength and structure of the association between origin and destination class under nested modeling assumptions (Equations 1–14). The modeling sequence followed established practice in comparative mobility research and included:

Conditional independence model (baseline) which assesses the strength of association between origin and destination while accounting for the marginal distributions of layer variables.

Baseline conditional independence model with OD not subsumed [PCO PCD OD] for cohorts as layer variable for example, was calculated as follows:


$$\begin{array}{c} LogFe_{inlk}=\lambda^{G}+\lambda_{i}^{O}+\lambda_{n}^{D}+\lambda_{l}^{C}+\lambda_{k}^{P}+\lambda_{ik}^{PO}+\lambda_{nk}^{PD}+\lambda_{il}^{CO}\\ +\;\lambda_{jn}^{CD}+\lambda_{lk}^{PC}+\lambda_{ilk}^{PCO}+\lambda_{nlk}^{PCD}+\lambda_{in}^{OD} \end{array}$$ (1)

Three layers baseline conditional independence model with OD not subsumed [PCSO PCSD OD] as follows:


$$\begin{array}{l} LogFe_{inlk}=\lambda^{G}+\lambda_{i}^{O}+\lambda_{n}^{D}+\lambda_{k}^{P}+\lambda_{l}^{C}+\lambda_{J}^{S}+\lambda_{ik}^{PO}+\lambda_{nk}^{PD}\\ \;+\;\lambda_{il}^{CO}+\;\lambda_{nl}^{CD}+\lambda_{ij}^{SO}\;+\lambda_{nj}^{SD}+\lambda_{lk}^{PC}+\lambda_{jk}^{PS}++\lambda_{jl}^{CS}\\ \;+\lambda_{ilk}^{PCO}+\lambda_{nlk}^{PCD}+\lambda_{ijk}^{PSO}+\lambda_{njk}^{PSD}+\lambda_{ijl}^{CSO}\\ \;+\;\lambda_{njl}^{CSD}+\lambda_{ijlk}^{PCSO}+\lambda_{njlk}^{PCSD}+\lambda_{in}^{OD} \end{array}$$ (2)

Homogeneous association or constant social fluidity (CnSF) model tests whether the origin–destination association remains constant across country, cohort or gender.

Homogeneous association across countries [PCO PCD POD] as follows:


$$\begin{array}{c} LogFe_{inlk}=\lambda^{G}+\lambda_{i}^{O}+\lambda_{n}^{D}+\lambda_{l}^{C}+\lambda_{k}^{P}+\lambda_{ik}^{PO}+\lambda_{nk}^{PD}+\lambda_{il}^{CO}\\ +\;\lambda_{jn}^{CD}+\lambda_{lk}^{PC}+\lambda_{ilk}^{PCO}+\lambda_{nlk}^{PCD}+\lambda_{nik}^{POD} \end{array}$$ (3)

Homogeneous association across cohorts [PCO PCD COD]:


$$\begin{array}{c} LogFe_{inlk}=\lambda^{G}+\lambda_{i}^{O}+\lambda_{n}^{D}+\lambda_{l}^{C}+\lambda_{k}^{P}+\lambda_{ik}^{PO}+\lambda_{nk}^{PD}+\lambda_{il}^{CO}\\ +\;\lambda_{jn}^{CD}+\lambda_{lk}^{PC}+\lambda_{ilk}^{PCO}+\lambda_{nlk}^{PCD}+\lambda_{nil}^{COD} \end{array}$$ (4)

Homogeneous association across gender [PSO PSD SOD]:


$$\begin{array}{c} LogFe_{inlk}=\lambda^{G}+\lambda_{i}^{O}+\lambda_{n}^{D}+\lambda_{l}^{S}+\lambda_{k}^{P}+\lambda_{ik}^{PO}+\lambda_{nk}^{PD}+\lambda_{il}^{SO}\\ +\;\lambda_{jn}^{SD}+\lambda_{lk}^{PC}+\lambda_{ilk}^{PSO}+\lambda_{nlk}^{PSD}+\lambda_{nil}^{SOD} \end{array}$$ (5)

Homogeneous association across countries and cohorts [PCO PCD POD COD]:


$$\begin{array}{c} LogFe_{inlk}=\lambda^{G}+\lambda_{i}^{O}+\lambda_{n}^{D}+\lambda_{l}^{C}+\lambda_{k}^{P}+\lambda_{ik}^{PO}+\lambda_{nk}^{PD}+\lambda_{il}^{CO}\\ +\;\lambda_{jn}^{CD}+\lambda_{lk}^{PC}+\lambda_{ilk}^{PCO}+\lambda_{nlk}^{PCD}+\lambda_{nik}^{POD}+\lambda_{nil}^{COD} \end{array}$$ (6)

Homogeneous association across countries and gender [PSO PSD POD SOD]:


$$\begin{array}{c} LogFe_{inlk}=\lambda^{G}+\lambda_{i}^{O}+\lambda_{n}^{D}+\lambda_{l}^{S}+\lambda_{k}^{P}+\lambda_{ik}^{PO}+\lambda_{nk}^{PD}+\lambda_{il}^{SO}\\ +\;\lambda_{jn}^{SD}+\lambda_{lk}^{PS}+\lambda_{ilk}^{PSO}+\lambda_{nlk}^{PSD}+\lambda_{nik}^{POD}+\lambda_{nil}^{SOD} \end{array}$$ (7)

Homogeneous association across countries, cohorts and gender simultaneously [PCSO PCSD PCOD PSOD CSOD]:


$$\begin{array}{l} LogFe_{inlk}=\lambda^{G}+\lambda_{i}^{O}+\lambda_{n}^{D}+\lambda_{k}^{P}+\lambda_{l}^{C}+\lambda_{J}^{S}+\lambda_{ik}^{PO}+\lambda_{nk}^{PD}+\lambda_{il}^{CO}\\ \;+\;\lambda_{nl}^{CD}+\lambda_{ij}^{SO}\;+\lambda_{nj}^{SD}+\lambda_{lk}^{PC}+\lambda_{jk}^{PS}++\lambda_{jl}^{CS}+\lambda_{ilk}^{PCO}\\ \;+\;\lambda_{nlk}^{PCD}+\lambda_{ijk}^{PSO}+\lambda_{njk}^{PSD}+\lambda_{ijl}^{CSO}+\lambda_{njl}^{CSD}+\lambda_{ijlk}^{PCSO}\\ \;+\;\lambda_{njlk}^{PCSD}+\lambda_{nilk}^{PCOD}+\lambda_{nijk}^{PSOD}+\lambda_{nijl}^{CSOD}+\lambda_{in}^{OD} \end{array}$$ (8)

While uniform difference (Unidiff) model evaluates whether any variation in the association across contextual dimensions follows a uniform scaling pattern rather than structural change. Scaling parameters were estimated relative to Mexico (reference value = 1.000).

Uniform difference (Unidiff) across countries [PCO PCD spe(OD,1a,P,b)]:


$$\begin{array}{l} LogFe_{inlk}=\lambda^{G}+\lambda_{i}^{O}+\lambda_{n}^{D}+\lambda_{l}^{C}+\lambda_{k}^{P}+\lambda_{ik}^{PO}+\lambda_{nk}^{PD}+\lambda_{il}^{CO}\\ \;+\;\lambda_{jn}^{CD}+\lambda_{lk}^{PC}+\lambda_{ilk}^{PCO}+\lambda_{nlk}^{PCD}+\lambda_{in}^{OD}+\varphi_{P}\;\psi_{in}^{OD},\\ \;just\;if\;{\varphi_{P}}_{2}<1 \end{array}$$ (9)

Uniform difference (Unidiff) across cohorts [PCO PCD spe(OD,1a,C,b)]:


$$\begin{array}{l} LogFe_{inlk}=\lambda^{G}+\lambda_{i}^{O}+\lambda_{n}^{D}+\lambda_{l}^{C}+\lambda_{k}^{P}+\lambda_{ik}^{PO}+\lambda_{nk}^{PD}+\lambda_{il}^{CO}\\ \;+\;\lambda_{jn}^{CD}+\lambda_{lk}^{PC}+\lambda_{ilk}^{PCO}+\lambda_{nlk}^{PCD}+\lambda_{in}^{OD}+\varphi_{C}\;\psi_{in}^{OD},\\ \;just\;if\;{\varphi_{C}}_{2}<1 \end{array}$$ (10)

Uniform difference (Unidiff) across gender [PCO PCD spe(OD,1a,S,b)]:


$$\begin{array}{l} LogFe_{inlk}=\lambda^{G}+\lambda_{i}^{O}+\lambda_{n}^{D}+\lambda_{l}^{S}+\lambda_{k}^{P}+\lambda_{ik}^{PO}+\lambda_{nk}^{PD}+\lambda_{il}^{SO}\\ \;+\;\lambda_{jn}^{SD}+\lambda_{lk}^{PS}+\lambda_{ilk}^{PSO}+\lambda_{nlk}^{PSD}+\lambda_{in}^{OD}+\varphi_{S}\;\psi_{in}^{OD},\\ \;just\;if\;{\varphi_{S}}_{2}<1 \end{array}$$ (11)

Uniform difference (Unidiff) across countries and cohorts [PCO PCD spe(OD,1a,PC,b)]:


$$\begin{array}{l} LogFe_{inlk}=\lambda^{G}+\lambda_{i}^{O}+\lambda_{n}^{D}+\lambda_{l}^{C}+\lambda_{k}^{P}+\lambda_{ik}^{PO}+\lambda_{nk}^{PD}+\lambda_{il}^{CO}\\ \;+\;\lambda_{jn}^{CD}+\lambda_{lk}^{PC}+\lambda_{ilk}^{PCO}+\lambda_{nlk}^{PCD}+\lambda_{in}^{OD}+\varphi_{P}\varphi_{C}\;\psi_{in}^{OD},\\ \;just\;if\;{\varphi_{P}}_{2}<1\;and\;if\;{\varphi_{C}}_{2}<1 \end{array}$$ (12)

Uniform difference (Unidiff) across countries and gender [PSO PSD spe(OD,1a,PS,b)]:


$$\begin{array}{l} LogFe_{inlk}=\lambda^{G}+\lambda_{i}^{O}+\lambda_{n}^{D}+\lambda_{l}^{S}+\lambda_{k}^{P}+\lambda_{ik}^{PO}+\lambda_{nk}^{PD}+\lambda_{il}^{SO}\\ \;+\;\lambda_{jn}^{SD}+\lambda_{lk}^{PS}+\lambda_{ilk}^{PSO}+\lambda_{nlk}^{PSD}+\lambda_{in}^{OD}+\varphi_{P}\varphi_{S}\;\psi_{in}^{OD},\\ \;just\;if\;{\varphi_{P}}_{2}<1\;and\;if\;{\varphi_{S}}_{2}<1 \end{array}$$ (13)

Uniform difference (Unidiff) across countries, cohorts and gender simultaneously [ PCSO PCSD spe(OD,1a,PCS,b)]:


$$\begin{array}{l} LogFe_{inlk}=\lambda^{G}+\lambda_{i}^{O}+\lambda_{n}^{D}+\lambda_{k}^{P}+\lambda_{l}^{C}+\lambda_{J}^{S}+\lambda_{ik}^{PO}+\lambda_{nk}^{PD}+\lambda_{il}^{CO}\\ \;+\;\lambda_{nl}^{CD}+\lambda_{ij}^{SO}\;+\lambda_{nj}^{SD}+\lambda_{lk}^{PC}+\lambda_{jk}^{PS}++\lambda_{jl}^{CS}+\lambda_{ilk}^{PCO}\\ \;+\;\lambda_{nlk}^{PCD}+\lambda_{ijk}^{PSO}+\lambda_{njk}^{PSD}+\lambda_{ijl}^{CSO}+\lambda_{njl}^{CSD}+\lambda_{ijlk}^{PCSO}\\ \;+\;\lambda_{njlk}^{PCSD}+\lambda_{nilk}^{PCOD}+\lambda_{nijk}^{PSOD}+\lambda_{nijl}^{CSOD}+\lambda_{in}^{OD}\\ \;+\;\varphi_{P}\varphi_{C}\varphi_{S}\;\psi_{in}^{OD},\\ \;just\;if\;{\varphi_{P}}_{2}<1,\;{\varphi_{C}}_{2}<1\;and\;{\varphi_{S}}_{2}<1 \end{array}$$ (14)

Multiple layers model estimation proceeded hierarchically, with independence models serving as a diagnostic baseline and CnSF and Unidiff models providing increasingly flexible structure to detect patterned variation rather than random heterogeneity. For a deeper review of the techniques see also Boado et al. (2019), Vallet (2020), and Ricardi-Morgavi (2022).

### Goodness-of-fit evaluation

Model fit was evaluated using multiple criteria, as likelihood ratio chi-square statistic (*L*^2^), degrees of freedom, Bayesian information criterion (BIC), and the index of dissimilarity. Lower values of *L*^2^ and BIC indicate better fit while accounting for model complexity and parsimony. The index of dissimilarity provides a summary measure of misclassification and is expressed as a percentage for interpretability.

In the case of the four-way (PCOD and PSOD) and five-way (PCSOD) models, a value of 0.05 was assigned to all cells with zero counts. This adjustment represents a negligible amount that does not affect the substantive results but prevents estimation problems associated with empty cells in the calculation algorithm. As a alternative, Firth's method (1993), recommended by Breen (2004), proposes assigning a constant equal to the number of model parameters divided by twice the number of cells in the contingency table. In this case, these values correspond to 0.20 and 0.19, respectively. Following Jorrat (2014), the results obtained using this alternative correction do not differ substantially from those derived using the more conventional imputation of 0.05.

### Software and computational environment

All models were estimated using LEM statistical software (Vermunt, 1997), capable of handling high-dimensional mobility tables and specialized log-linear modeling routines. Estimates were replicated and checked for convergence stability and parameter interpretability before model comparison.

This study uses anonymized secondary survey data. No personally identifiable information was accessed, and no intervention was performed. All procedures complied with ethical guidelines for the use of public comparative survey data. The data used for the analysis can be downloaded from the following link: LEM data files.

## Results

Patterns of intergenerational class mobility exhibit both shared structural features and identifiable contextual variation across countries, cohorts, and gender. Across all specifications, the association between parental class and respondents' class of destination remained statistically significant, indicating a persistent link between origin and destination in the observed societies. However, the strength of this association varied depending on country context and, to a lesser extent, birth cohort and gender.

### Overall patterns of association

Across the full set of models, baseline independence models demonstrated the weakest fit, as reflected in high likelihood ratio values and higher dissimilarity percentages. These results indicate that parental class and respondents' class destinations are not independent once the marginal distributions of country, cohort, and gender are controlled. In contrast, constant social fluidity (CnSF) models substantially improved model fit across all analytical configurations, suggesting that the structure of class mobility—rather than the marginal distributions alone—plays a central role in generating observed patterns (Table A1 in Appendix).

### Cross-national differences

**Figure 2:**
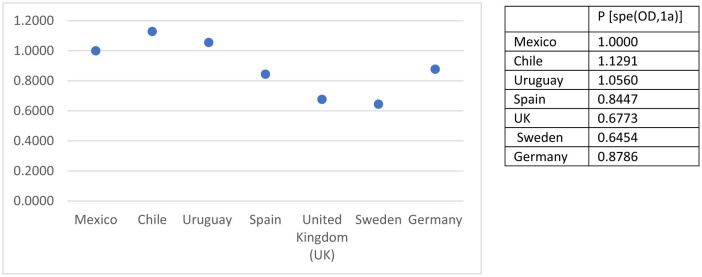
Unidiff parameters of O–D association through countries. P, countries.

Comparisons across countries show structured heterogeneity rather than random or unpatterned variation. In the uniform difference (Unidiff) model the criteria for scaling in Mexico as the reference category (1.000) is theoretical, that is, it is of interest to know the fluidity pattern from the deviations of the Latin American universalist welfare regimes (Chile and Uruguay) and the European typology of advanced economies with respect to a regressive and dual regime (Mexico). The strongest association between origin and destination was observed in Chile (1.1291) and Uruguay (1.0560), while the weakest associations were observed in the United Kingdom (0.6773) and Sweden (0.6454). Spain (0.8447), and Germany (0.8786) occupied an intermediate position (Figure 2). These estimates indicate that countries differ in the extent to which parental class influences the class destinations of their children, with the set of Latin American countries showing higher intergenerational persistence and the northern European cases showing comparatively weaker associations. These results are derived from the uniform difference parameter.

### Variation across countries and birth cohorts

**Figure 3:**
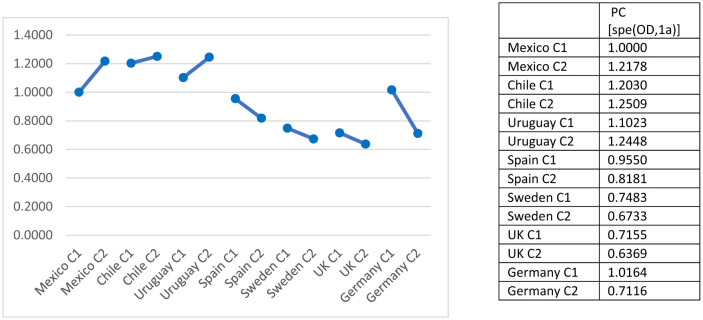
Unidiff parameters of O–D association through countries and cohorts. All together combined visualization. C = cohorts, C1 = cohort 1930–1975, C2 = cohort 1976–1990.

The joint specification including country and cohort revealed modest, but patterned, temporal variation (Figure 3). Taking Mexico first cohort (C1) as baseline, uniform difference estimates increased for the younger cohort in Mexico, Chile, and Uruguay—indicating stronger intergenerational class association among individuals born between 1976 and 1990. In contrast, Spain, Sweden, and the United Kingdom show decreasing parameter values across cohorts, suggesting a relative weakening of class inheritance in the younger cohort, but the association has also been weaker in the older cohort in all European countries compared to this same cohort in Mexico as well as in Chile and Uruguay. Germany showed a mixed pattern, with an increase in inheritance among the older cohort—surpassing Mexico and related to the lasting effects of the Second World War, which stalled the occupational trajectories of those born between 1930 and 1950, and the interlude of the Cold War until the fall of the Berlin Wall—and a decline among the younger one.^1^ These estimates suggest that temporal change is not universal but contingent on national context.

### Gendered dimensions of mobility

**Figure 4:**
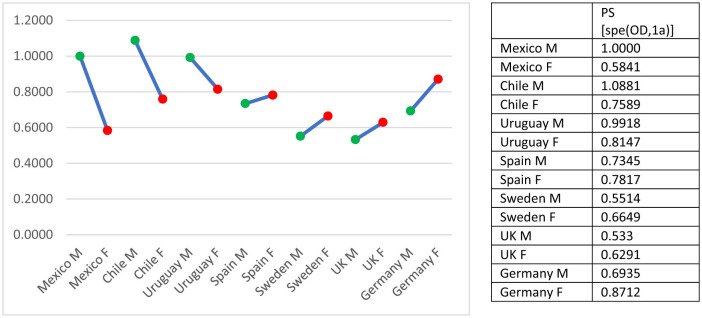
Unidiff parameters of O–D association through countries and gender. All together combined visualization. S, gender; M, male; F, Female.

Gender differences also emerged as structured rather than random. Among men, uniform difference estimates were relatively high in Mexico (1.000), Chile (1.0881), and Uruguay (0.9918), while Sweden (0.5514), and the United Kingdom (0.5330) showed markedly lower values (Figure 4). Among women, observed variation was narrower, although still notable. Sweden, Spain, and the United Kingdom displayed weaker associations for women than men, while Germany showed the reverse pattern, with higher association among women. These gendered patterns suggest that changes in educational expansion and labor market participation have influenced mobility differently across institutional contexts.

### Combined contexts: country, cohort, and gender

**Figure 5:**
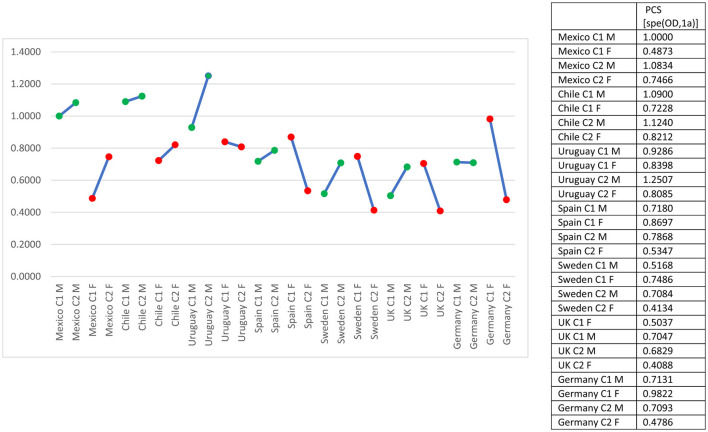
Unidiff parameters of O–D association through countries, cohorts, and gender. All together combined visualization. S, gender; M, male; F, Female.

The most detailed specification—jointly modeling country, cohort, and gender—revealed multi-axis heterogeneity. For example, the strongest associations occurred among Chilean and Uruguayan men in the younger cohort, while the weakest were observed among women in Sweden and the United Kingdom. Mexico showed a widening gender gap across cohorts, with stronger persistence among men and lower associations among women in the younger cohort (Figure 5). These joint patterns confirm that the relationship between origin and destination is shaped by interacting national, historical, and gendered contexts rather than a single uniform mechanism.

**Figure 6:**
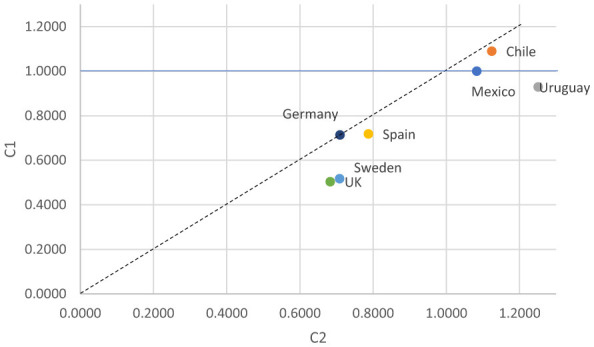
Changes in Unidiff parameters of O–D association across countries and cohorts among men.

The male scatterplot (Figure 6) indicates a remarkably consistent pattern across all countries, every case lies below the 45° diagonal, meaning that the Unidiff parameters for the younger cohort (C2) are higher than those of the older cohort (C1). It implies that class-origin effects have strengthened over time for men in every country considered, with no evidence of increasing fluidity in the younger generation.

A possible age-related concern is that cohort estimates may partly capture differences in occupational maturity, especially where younger workers have not yet completed their mobility trajectories. However, the overall cross-national consistency of the pattern suggests that the result is not reducible to a single country-specific age composition artifact, although this limitation should be acknowledged cautiously.

Despite this shared trend toward greater rigidity, countries differ in their relative levels of O–D association. Uruguay exhibits the strongest increases, positioning it as the most rigid case in C2. Chile and Mexico, while also below the diagonal, show a more moderate rise, representing an intermediate level of persistence. By contrast, European countries—Spain, Sweden, and the United Kingdom—cluster at lower absolute levels of both C1 and C2, but they nonetheless follow the same directional pattern—a clear decrease of fluidity among younger men.

This implies that, contrary to expectations derived from modernization theory, the barriers associated with class origin have become more pronounced in the context of the technological restructuring and flexibilization of the labor market during the pro-market (neoliberal) macroeconomic policies in Latin American and postindustrial age in European nations.

Taken together, the plot reveals two distinct regional patterns: (1) Latin American countries displaying rising rigidity and strong persistence of class disadvantage, and (2) European countries—except for Germany—maintaining fluidity (weak class-origin effects over time) although it is diminishing.

**Figure 7:**
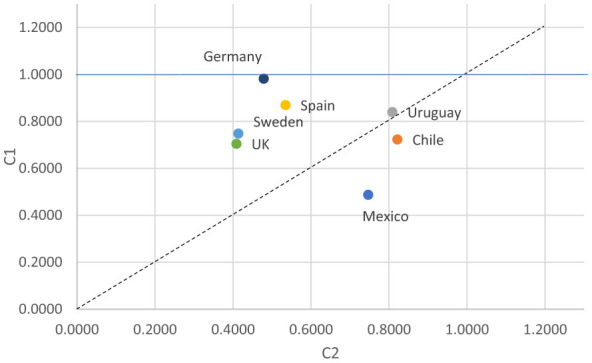
Changes in Unidiff parameters of O–D association across countries and cohorts among women.

The scatterplot for women (Figure 7) reveals substantial cross-national variation in the evolution of intergenerational class associations. European countries—Germany, Spain, Sweden, and the UK—cluster above the diagonal, indicating that younger female cohorts experience weaker intergenerational constraints than their predecessors. Although the magnitude varies, all four cases suggest that institutional changes associated with expanding welfare states, labor-market integration of women, and educational equalization have contributed to a declining influence of class origin. Sweden and the UK show relatively lower O–D association in both cohorts, while Germany and Spain begin from lower levels and move in the direction of increasing fluidity.

In contrast, Chile and Mexico lie below the diagonal, signaling greater rigidity in the younger cohort of women. For these two Latin American countries, class-origin effects have strengthened over time. Chile, positioned closer to the diagonal, shows a modest increase in rigidity, whereas Mexico exhibits a more pronounced shift, reflecting a stronger intergenerational reproduction of class among younger women. Uruguay lies very close to the diagonal, indicating relative stability in women's O–D association across cohorts rather than a clear movement toward either rigidity or fluidity.

The female pattern highlights the divergent regional dynamics of mobility. Europe shows a consistent trend toward weaker class-origin effects for women, while parts of Latin America—especially Mexico and Chile—experience a reinforcement of intergenerational inequalities among younger cohorts, suggesting that structural heterogeneity, segmented labor markets, and persistent inequalities in educational and occupational opportunities continue to shape women's mobility.

The Mexican outlier may partly reflect age-composition effects if younger women are observed at earlier and less consolidated stages of their occupational trajectories, but its magnitude also suggests a substantive pattern of strengthened class reproduction. By contrast, Uruguay appears closer to stability, so the stronger increase noted for men there should be interpreted as a gender-specific pattern rather than a general cohort effect.

## Conclusions

The results provide evidence of both continuity and structured variation in the association between individuals' social class of origin and their class of destination. Across all model configurations, the association remained statistically significant, indicating that social origins continue to shape class outcomes in each of the seven countries. This finding aligns with longstanding observations in mobility research that inequalities in life chances persist even in the presence of educational expansion, labor market restructuring, or welfare interventions. At the same time, the results reveal that the strength of this association varies systematically across countries, cohorts, and gender, indicating that mobility regimes are neither universally stable nor uniformly shifting.

### Substantive interpretation: national variation in mobility regimes

Cross-national variation in uniform difference parameters suggests that countries differ in the extent to which class advantages are transmitted intergenerationally. The higher parameter values observed in Mexico, Chile, and Uruguay indicate stronger persistence of parental class in shaping adult outcomes, whereas weaker associations in Sweden and the United Kingdom reflect comparatively more fluid mobility structures. Spain and Germany occupy intermediate positions, with neither strong convergence toward the most fluid cases nor alignment with the highest persistence cases. These patterns underscore the importance of understanding mobility not as a universal phenomenon but as a contextual process embedded in institutional configurations, economic structures, and welfare arrangements.

### Cohort differences and historical dynamics

Temporal patterns observed across the two birth cohorts reveal that mobility regimes are not static. The evidence suggests diverging cohort trajectories—increases in class persistence across the younger cohorts in Mexico, Chile, and Uruguay contrast with reductions in the strength of origin–destination associations in Spain, Sweden, and the United Kingdom. These differences may reflect contrasting historical developments, including the pace and nature of educational expansion, labor market restructuring, and shifts in state intervention. The mixed pattern observed in Germany suggests that mobility dynamics may be shaped by historical events that interact with the timing of cohort exposure, such as economic integration, reunification and welfare reforms.

### Gendered patterns of mobility

Gender differences indicate that mobility regimes are not neutral with respect to sex. In several countries, the association between parental class and class destination is weaker among women, particularly in younger cohorts. This may reflect expanded educational access and increased participation in formal labor markets. However, in the Mexican younger cohort and German older one, stronger associations among women suggest a different relationship between gender roles and stratification processes, possibly linked to labor market segmentation, occupational norms, or cultural expectations around family and caregiving. The variation observed across countries highlights the need to consider gender as an independent axis of stratification rather than a secondary or population adjustment variable.

### Methodological contribution and interpretation

The modeling sequence demonstrates the utility of progressing from conditional independence models to constant social fluidity and uniform difference models. Constant social fluidity models substantially improved fit relative to independence models, showing that the structure of mobility is not solely a function of marginal distributions. At the same time, the improvements seen in uniform difference models make clear that variation is not random but follows patterned multiplicative scaling. By applying a harmonized modeling strategy across seven countries, two cohorts, and gender, the analysis demonstrates how log-linear frameworks can detect structured heterogeneity in mobility regimes while maintaining comparability.

Taken together, the results show that intergenerational class mobility patterns are shaped by the interaction of institutional context, historical timing, and gendered social structures. The persistence of origin–destination associations across all societies suggests that mobility remains constrained, yet the degree and form of constraint vary across settings. These findings emphasize the value of examining multiple dimensions of variation under loglinear modeling rather than assuming homogeneity across populations or uniform convergence over time under commonly applied statistic techniques as linear, logit or probit regressions.

### Contribution and limitations

This study makes several contributions to the comparative analysis of intergenerational class mobility. First, it provides a harmonized framework for examining social mobility across seven countries using a consistent class schema and analytical approach. While prior research has focused either on single-country analyses or on selected macro-regional comparisons, this design integrates contexts with diverse welfare models, institutional trajectories, and sociohistorical conditions. The inclusion of two birth cohorts and gender adds further analytical depth, enabling the identification of structured variation across demographic layers rather than relying on pooled or undifferentiated estimates.

Second, the study advances methodological practice by applying a sequential log-linear modeling framework that evaluates both stability and structured heterogeneity in mobility regimes. The use of conditional independence, constant social fluidity, and uniform difference models demonstrates how variation in mobility patterns can be assessed systematically without assuming either universal convergence or unrestricted contextual divergence. This application illustrates the flexibility of log-linear modeling for comparative stratification research and highlights the interpretive value of uniform scaling parameters.

Third, the joint examination of country, cohort, and gender demonstrates the utility of multidimensional mobility analysis. The findings show that variation does not occur uniformly across contexts, nor does it follow a single direction of change. Instead, fluidity patterns appear contingent on the interaction of institutional arrangements, historical timing, and gendered social roles. This multidimensional approach can support future research seeking to understand how mobility regimes evolve in response to changing social structures.

Despite these contributions, several limitations should be acknowledged. First, although harmonization was applied consistently, the analysis relies on secondary survey data, which may reflect variation in sampling frames, occupational coding procedures, or measurement precision across countries. Second, the two birth cohort categories, while analytically useful, aggregate within-cohort variability and do not capture finer temporal shifts. Third, class schema harmonization necessarily simplifies national occupational hierarchies and may mask meaningful institutional distinctions. Finally, the models capture associations rather than causal mechanisms, and the results should therefore be interpreted as descriptive comparisons rather than evidence of causal processes.

A further limitation concerns the potential overlap between cohort, age, and period effects. Although the cohort design aims to capture broad generational shifts in mobility regimes, differences in occupational maturity across age ranges may partly affect the observed associations. In addition, the German case should be interpreted with caution, since postwar mortality shocks and subsequent labor migration may introduce period-specific effect that are not fully comparable to the other national cases.

Future research may address these limitations by incorporating additional cohorts, longitudinal data, or multilevel extensions that explicitly model nested social structures. Expansion to additional countries or welfare contexts, as well as integration with educational or income mobility metrics, may further clarify how mobility regimes evolve across time and interact with broader processes of social stratification.

The results indicate that social class of origin continues to influence class outcomes in all contexts analyzed, although the strength of association varies across countries, birth cohorts, and gender. Countries in Latin America exhibited stronger intergenerational persistence, while Sweden and the United Kingdom demonstrated comparatively greater fluidity. Temporal and gendered differences were present but heterogeneous across national settings. These findings underscore the importance of comparative analytical frameworks that consider the interaction of institutional, historical, and demographic dimensions. The results contribute to ongoing debates on social openness and mobility by demonstrating that mobility regimes are neither fixed nor universally convergent but dynamic and contextually structured.

From the vantage point of the Unidiff estimates obtained in this study, these institutional trajectories help explain why the Latin American cases—Mexico, Chile, and Uruguay—display higher scaling parameters and thus stronger origin–destination persistence, whereas Sweden and the United Kingdom, followed by Germany and Spain, exhibit comparatively weaker class inheritance. In the Latin American context, the combination of corporatist segmentation, incomplete universalization, and later neoliberal restructuring appears to have produced durable barriers to mobility, particularly for working-class and rural-origin individuals whose trajectories remained conditioned by unequal access to education and formal employment.

Conversely, in the European cases—especially Sweden and the United Kingdom—the earlier maturation of welfare institutions, stronger alignment between education and occupational upgrading, and broader gender inclusion partially loosened the link between class origins and destinations, yielding higher relative mobility even amid later flexibilization pressures. The intermediate patterns observed for Spain and Germany align with their hybrid regime trajectories: significant welfare expansion and educational democratization for the older cohort, followed by reforms that introduced uncertainty and polarization for the younger cohort.

Importantly, the gendered patterns evident in the younger cohort reflect these same institutional differences, as expanding female labor participation intersects with welfare regimes that either incorporate or stratify new entrants. Thus, rather than supporting convergence or uniform cohort change, the results point to structured heterogeneity in mobility regimes, consistent with the expectation that social fluidity is shaped not only by modernization but by historically contingent institutional pathways and policy architectures.

## Data Availability

The datasets presented in this study can be found in online repositories. The names of the repository/repositories and accession number(s) can be found in the article/Supplementary material.

## Footnotes

1 Period effects are likely to explain the pattern observed in Germany because of the increased mortality of young and middle-age males during the Second World War. The German labor force after this was comprised a significant proportion of migrants from Turkey, Portugal, Spain, Italy and, until 1961, from Eastern Europe.

## Appendix

**Table A1:** Model adjustment measures.

Loglinear models	*L* ^2^	gl	BIC	Index of dissimilarity
Set 1. for O, D, P y C models				
Baseline [PCO PCD OD]	728.446	208	–1292.63	6.3%
CnSF countries [PCO PCD POD]	263.769	112	–824.50	3.6%
Unidiff countries [PCO PCD spe(OD,1a,P,b)]	689.683	202	–1273.09	5.9%
CnSF cohorts [PCO PCD COD]	664.550	192	–1201.06	5.9%
Unidiff cohorts [PCO PCD spe(OD,1a,C,b)]	726.319	207	–1285.04	6.2%
CnSF countries and cohorts [PCO PCD POD COD]	207.026	96	–725.78	3.2%
Unidiff countries and cohorts [PCO PCD spe(OD,1a,PC,b)]	677.497	195	–1217.26	5.8%
Set 2 for O, D, P y S models				
Baseline [PSO PSD OD]	766.362	208	–1254.71	6.8%
CnSF countries [PSO PSD POD]	292.331	112	–795.94	4.6%
Unidiff countries [PSO PSD spe(OD,1a,P,b)]	731.310	202	–1231.46	6.6%
CnSF gender [PSO PSD SOD]	665.316	192	–1200.29	6.2%
Unidiff gender [PSO PSD spe(OD,1a,S,b)]	748.101	207	–1263.26	6.7%
CnSF countries and gender [PSO PSD POD SOD]	191.812	96	–740.99	3.5%
Unidiff countries and gender [PSO PSD spe(OD,1a,PS,b)]	679.982	195	–1214.78	5.9%
Set 3 for O, D, P, C, S models				
Baseline [PCSO PCSD OD]	1182.027	432	–3016.05	8.3%
CnSF countries, cohorts and gender [PCSO PCSD PCOD PSOD CSOD]	152.229	96	–780.68	2.6%
Unidiff countries, cohorts and gender [PCSO PCSD spe(OD,1a,PCS,b)]	1068.664	405	–2867.03	7.4%

P, country; C, cohort; S, gender.
