# Supplementary material for: Comparing patterns of intergenerational class mobility using log-linear models: evidence from seven countries, two cohorts, and gendered stratification
Source: Front Sociol. 2026 May 1;11:1757240. doi: 10.3389/fsoc.2026.1757240 (PMC13177305; doi:10.3389/fsoc.2026.1757240)
Supplement: Supplementary file 1 [file Data_Sheet_1.pdf]

LEM: log-linear and event history analysis with missing data.
Developed by Jeroen Vermunt (c), Tilburg University, The Netherlands.
Version 1.0 (September 18, 1997).

*** INPUT ***

```
 man 4
  dim 7 2 5 5
  lab P C O D
  *  mod {PCO PCD OD}
  *  mod {PCO PCD POD}
  *  mod {PCO PCD spe(OD,1a,P,b)}
  *  mod {PCO PCD COD}
  *  mod {PCO PCD spe(OD,1a,C,b)}
  *  mod {PCO PCD POD COD}
    mod {PCO PCD spe(OD,1a,PC,b)}
 add .05
 nse

  dat[101	11	19	17	44
 62	25	15	28	70
 180	76	185	70	116
 85	60	67	62	131
 285	279	390	265	1066
 96	57	19	14	5
 21	75	14	9	39
 115	152	88	51	103
 21	72	21	69	78
 143	195	132	141	542
 75	15	25	1	31
 30	27	11	20	28
 43	31	46	40	26
 60	46	45	77	119
 85	104	103	137	448
 69	21	14	10	6
 19	36	2	18	23
 46	33	17	17	34
 67	53	27	77	47
 66	101	30	84	241
 96	32	24	9	16
 91	43	27	15	30
 95	35	52	16	37
 34	30	43	38	81
 37	48	58	50	120
 44	21	4	11	6
 28	29	6	9	6
 26	21	10	16	22
```

8	29	8	20	34
11	30	19	38	66
70	54	16	13	33
23	17	7	8	14
44	43	48	37	61
29	45	14	33	45
36	60	29	51	155
54	34	4	12	17
13	14	1	5	9
10	22	8	7	12
11	26	1	13	15
18	41	5	27	32
181	61	15	20	32
11	13	1	4	6
59	38	28	17	25
110	69	13	48	31
46	56	10	29	36
52	39	0	10	13
5	9	0	1	1
5	4	3	5	4
19	22	0	12	19
11	27	1	5	10
166	58	14	20	34
8	14	1	4	6
55	36	26	18	20
107	69	14	49	31
42	55	12	28	32
57	40	0	11	16
5	10	0	1	1
6	4	3	5	4
20	22	0	12	18
12	28	1	5	11
258	85	29	35	44
49	42	0	16	19
18	0	37	9	2
212	191	19	128	112
95	112	8	96	142
71	43	1	21	13
13	13	0	8	6
3	0	8	0	0
56	60	1	40	26
25	29	0	17	29

]

*Order of the countries: Mexico, Chile, Uruguay, Spain, Sweden, UK and Germany.
*Order of the cohorts: old 1930-1975; youth 1976-1990.

*** STATISTICS ***

Number of iterations = 148
Converge criterion = 0.0000009245

X-squared = 819.4361 (0.0000)
L-squared = 677.4967 (0.0000)
Cressie-Read = 729.5879 (0.0000)
Dissimilarity index = 0.0581
Degrees of freedom = 195
Log-likelihood = -85965.13387
Number of parameters = 154 (+1)
Sample size = 16592.5
BIC(L-squared) = -1217.2610
AIC(L-squared) = 287.4967
BIC(log-likelihood) = 173426.6405
AIC(log-likelihood) = 172238.2677

WARNING: no information is provided on identification of parameters

*** FREQUENCIES ***

P C O D	observed	estimated	std. res.
1 1 1 1	101.050	94.674	0.655
1 1 1 2	11.050	23.387	-2.551
1 1 1 3	19.050	30.035	-2.004
1 1 1 4	17.050	12.276	1.363
1 1 1 5	44.050	31.878	2.156
1 1 2 1	62.050	61.249	0.102
1 1 2 2	25.050	39.462	-2.294
1 1 2 3	15.050	23.639	-1.766
1 1 2 4	28.050	21.351	1.450
1 1 2 5	70.050	54.550	2.099
1 1 3 1	180.050	167.236	0.991
1 1 3 2	76.050	76.833	-0.089
1 1 3 3	185.050	183.426	0.120
1 1 3 4	70.050	64.845	0.646
1 1 3 5	116.050	134.911	-1.624
1 1 4 1	85.050	86.032	-0.106
1 1 4 2	60.050	56.635	0.454
1 1 4 3	67.050	63.585	0.434

1 1 4 4	62.050	67.949	-0.716
1 1 4 5	131.050	131.048	0.000
1 1 5 1	285.050	304.060	-1.090
1 1 5 2	279.050	254.933	1.510
1 1 5 3	390.050	375.564	0.747
1 1 5 4	265.050	275.829	-0.649
1 1 5 5	1066.050	1074.863	-0.269
1 2 1 1	96.050	90.094	0.627
1 2 1 2	57.050	45.936	1.640
1 2 1 3	19.050	18.182	0.203
1 2 1 4	14.050	11.623	0.712
1 2 1 5	5.050	25.415	-4.040
1 2 2 1	21.050	37.258	-2.655
1 2 2 2	75.050	61.054	1.791
1 2 2 3	14.050	9.547	1.458
1 2 2 4	9.050	16.029	-1.743
1 2 2 5	39.050	34.362	0.800
1 2 3 1	115.050	118.237	-0.293
1 2 3 2	152.050	128.349	2.092
1 2 3 3	88.050	108.094	-1.928
1 2 3 4	51.050	57.905	-0.901
1 2 3 5	103.050	96.665	0.649
1 2 4 1	21.050	42.237	-3.260
1 2 4 2	72.050	71.051	0.118
1 2 4 3	21.050	23.876	-0.578
1 2 4 4	69.050	49.198	2.830
1 2 4 5	78.050	74.887	0.365
1 2 5 1	143.050	108.423	3.326
1 2 5 2	195.050	244.860	-3.183
1 2 5 3	132.050	114.551	1.635
1 2 5 4	141.050	149.496	-0.691
1 2 5 5	542.050	535.921	0.265
2 1 1 1	75.050	73.864	0.138
2 1 1 2	15.050	19.953	-1.098
2 1 1 3	25.050	18.728	1.461
2 1 1 4	1.050	11.693	-3.112
2 1 1 5	31.050	23.011	1.676
2 1 2 1	30.050	31.410	-0.243
2 1 2 2	27.050	26.885	0.032
2 1 2 3	11.050	10.082	0.305
2 1 2 4	20.050	16.340	0.918
2 1 2 5	28.050	31.533	-0.620
2 1 3 1	43.050	44.557	-0.226
2 1 3 2	31.050	25.392	1.123
2 1 3 3	46.050	50.244	-0.592
2 1 3 4	40.050	26.346	2.670
2 1 3 5	26.050	39.712	-2.168
2 1 4 1	60.050	58.997	0.137

2 1 4 2	46.050	51.822	-0.802
2 1 4 3	45.050	41.376	0.571
2 1 4 4	77.050	82.095	-0.557
2 1 4 5	119.050	112.960	0.573
2 1 5 1	85.050	84.422	0.068
2 1 5 2	104.050	99.198	0.487
2 1 5 3	103.050	109.820	-0.646
2 1 5 4	137.050	138.776	-0.146
2 1 5 5	448.050	445.034	0.143
2 2 1 1	69.050	69.327	-0.033
2 2 1 2	21.050	22.163	-0.236
2 2 1 3	14.050	7.403	2.443
2 2 1 4	10.050	8.705	0.456
2 2 1 5	6.050	12.652	-1.856
2 2 2 1	19.050	30.271	-2.039
2 2 2 2	36.050	32.102	0.697
2 2 2 3	2.050	4.131	-1.024
2 2 2 4	18.050	13.096	1.369
2 2 2 5	23.050	18.651	1.019
2 2 3 1	46.050	45.486	0.084
2 2 3 2	33.050	31.599	0.258
2 2 3 3	17.050	22.924	-1.227
2 2 3 4	17.050	22.479	-1.145
2 2 3 5	34.050	24.762	1.867
2 2 4 1	67.050	56.440	1.412
2 2 4 2	53.050	61.487	-1.076
2 2 4 3	27.050	17.360	2.326
2 2 4 4	77.050	67.917	1.108
2 2 4 5	47.050	68.046	-2.545
2 2 5 1	66.050	65.726	0.040
2 2 5 2	101.050	96.900	0.422
2 2 5 3	30.050	38.431	-1.352
2 2 5 4	84.050	94.054	-1.031
2 2 5 5	241.050	227.140	0.923
3 1 1 1	96.050	100.752	-0.468
3 1 1 2	32.050	23.312	1.810
3 1 1 3	24.050	24.407	-0.072
3 1 1 4	9.050	9.229	-0.059
3 1 1 5	16.050	19.550	-0.792
3 1 2 1	91.050	73.509	2.046
3 1 2 2	43.050	48.932	-0.841
3 1 2 3	27.050	22.102	1.052
3 1 2 4	15.050	20.031	-1.113
3 1 2 5	30.050	41.676	-1.801
3 1 3 1	95.050	72.966	2.585
3 1 3 2	35.050	33.457	0.275
3 1 3 3	52.050	69.377	-2.080
3 1 3 4	16.050	22.357	-1.334

3 1 3 5	37.050	37.092	-0.007
3 1 4 1	34.050	56.667	-3.004
3 1 4 2	30.050	38.627	-1.380
3 1 4 3	43.050	34.870	1.385
3 1 4 4	38.050	38.038	0.002
3 1 4 5	81.050	58.048	3.019
3 1 5 1	37.050	49.356	-1.752
3 1 5 2	48.050	43.922	0.623
3 1 5 3	58.050	53.494	0.623
3 1 5 4	50.050	38.595	1.844
3 1 5 5	120.050	127.884	-0.693
3 2 1 1	44.050	42.710	0.205
3 2 1 2	21.050	19.581	0.332
3 2 1 3	4.050	6.522	-0.968
3 2 1 4	11.050	7.563	1.268
3 2 1 5	6.050	9.874	-1.217
3 2 2 1	28.050	19.135	2.038
3 2 2 2	29.050	28.933	0.022
3 2 2 3	6.050	3.729	1.202
3 2 2 4	9.050	11.605	-0.750
3 2 2 5	6.050	14.848	-2.283
3 2 3 1	26.050	23.310	0.567
3 2 3 2	21.050	23.137	-0.434
3 2 3 3	10.050	16.672	-1.622
3 2 3 4	16.050	16.139	-0.022
3 2 3 5	22.050	15.991	1.515
3 2 4 1	8.050	16.053	-1.997
3 2 4 2	29.050	24.931	0.825
3 2 4 3	8.050	7.024	0.387
3 2 4 4	20.050	26.947	-1.329
3 2 4 5	34.050	24.295	1.979
3 2 5 1	11.050	16.042	-1.246
3 2 5 2	30.050	33.668	-0.624
3 2 5 3	19.050	13.302	1.576
3 2 5 4	38.050	31.996	1.070
3 2 5 5	66.050	69.241	-0.384
4 1 1 1	70.050	74.235	-0.486
4 1 1 2	54.050	42.278	1.810
4 1 1 3	16.050	18.972	-0.671
4 1 1 4	13.050	16.709	-0.895
4 1 1 5	33.050	34.056	-0.172
4 1 2 1	23.050	15.488	1.921
4 1 2 2	17.050	22.035	-1.062
4 1 2 3	7.050	4.773	1.042
4 1 2 4	8.050	8.964	-0.305
4 1 2 5	14.050	17.989	-0.929
4 1 3 1	44.050	51.117	-0.988
4 1 3 2	43.050	52.653	-1.323

4 1 3 3	48.050	42.712	0.817
4 1 3 4	37.050	32.751	0.751
4 1 3 5	61.050	54.017	0.957
4 1 4 1	29.050	26.692	0.456
4 1 4 2	45.050	38.763	1.010
4 1 4 3	14.050	15.298	-0.319
4 1 4 4	33.050	33.738	-0.118
4 1 4 5	45.050	51.759	-0.933
4 1 5 1	36.050	34.718	0.226
4 1 5 2	60.050	63.520	-0.435
4 1 5 3	29.050	32.495	-0.604
4 1 5 4	51.050	50.088	0.136
4 1 5 5	155.050	150.428	0.377
4 2 1 1	54.050	49.497	0.647
4 2 1 2	34.050	38.667	-0.743
4 2 1 3	4.050	5.151	-0.485
4 2 1 4	12.050	12.426	-0.107
4 2 1 5	17.050	15.508	0.391
4 2 2 1	13.050	10.325	0.848
4 2 2 2	14.050	17.672	-0.862
4 2 2 3	1.050	1.262	-0.188
4 2 2 4	5.050	5.822	-0.320
4 2 2 5	9.050	7.170	0.702
4 2 3 1	10.050	15.428	-1.369
4 2 3 2	22.050	20.023	0.453
4 2 3 3	8.050	4.430	1.720
4 2 3 4	7.050	9.490	-0.792
4 2 3 5	12.050	9.879	0.691
4 2 4 1	11.050	12.920	-0.520
4 2 4 2	26.050	22.505	0.747
4 2 4 3	1.050	2.686	-0.998
4 2 4 4	13.050	14.223	-0.311
4 2 4 5	15.050	13.916	0.304
4 2 5 1	18.050	18.080	-0.007
4 2 5 2	41.050	38.383	0.431
4 2 5 3	5.050	5.721	-0.281
4 2 5 4	27.050	22.290	1.008
4 2 5 5	32.050	38.776	-1.080
5 1 1 1	181.050	178.381	0.200
5 1 1 2	61.050	66.050	-0.615
5 1 1 3	15.050	17.432	-0.571
5 1 1 4	20.050	22.279	-0.472
5 1 1 5	32.050	25.108	1.385
5 1 2 1	11.050	14.535	-0.914
5 1 2 2	13.050	11.028	0.609
5 1 2 3	1.050	1.645	-0.464
5 1 2 4	4.050	3.805	0.126
5 1 2 5	6.050	4.236	0.881

5 1 3 1	59.050	69.965	-1.305
5 1 3 2	38.050	41.216	-0.493
5 1 3 3	28.050	17.299	2.585
5 1 3 4	17.050	19.834	-0.625
5 1 3 5	25.050	18.936	1.405
5 1 4 1	110.050	94.403	1.610
5 1 4 2	69.050	72.787	-0.438
5 1 4 3	13.050	17.372	-1.037
5 1 4 4	48.050	45.575	0.367
5 1 4 5	31.050	41.113	-1.569
5 1 5 1	46.050	49.966	-0.554
5 1 5 2	56.050	46.169	1.454
5 1 5 3	10.050	13.503	-0.940
5 1 5 4	29.050	26.757	0.443
5 1 5 5	36.050	40.856	-0.752
5 2 1 1	52.050	50.773	0.179
5 2 1 2	39.050	38.976	0.012
5 2 1 3	0.050	1.623	-1.235
5 2 1 4	10.050	9.441	0.198
5 2 1 5	13.050	13.438	-0.106
5 2 2 1	5.050	4.820	0.105
5 2 2 2	9.050	7.055	0.751
5 2 2 3	0.050	0.176	-0.300
5 2 2 4	1.050	1.744	-0.526
5 2 2 5	1.050	2.455	-0.897
5 2 3 1	5.050	6.844	-0.686
5 2 3 2	4.050	7.979	-1.391
5 2 3 3	3.050	0.504	3.585
5 2 3 4	5.050	2.661	1.465
5 2 3 5	4.050	3.262	0.436
5 2 4 1	19.050	18.524	0.122
5 2 4 2	22.050	27.510	-1.041
5 2 4 3	0.050	1.046	-0.974
5 2 4 4	12.050	11.626	0.124
5 2 4 5	19.050	13.544	1.496
5 2 5 1	11.050	11.289	-0.071
5 2 5 2	27.050	19.731	1.648
5 2 5 3	1.050	0.901	0.157
5 2 5 4	5.050	7.778	-0.978
5 2 5 5	10.050	14.550	-1.180
6 1 1 1	166.050	163.032	0.236
6 1 1 2	58.050	64.647	-0.821
6 1 1 3	14.050	17.568	-0.839
6 1 1 4	20.050	22.822	-0.580
6 1 1 5	34.050	24.180	2.007
6 1 2 1	8.050	13.338	-1.448
6 1 2 2	14.050	10.502	1.095
6 1 2 3	1.050	1.654	-0.469

6 1 2 4	4.050	3.789	0.134
6 1 2 5	6.050	3.968	1.045
6 1 3 1	55.050	63.017	-1.004
6 1 3 2	36.050	38.955	-0.465
6 1 3 3	26.050	16.497	2.352
6 1 3 4	18.050	19.317	-0.288
6 1 3 5	20.050	17.465	0.619
6 1 4 1	107.050	91.805	1.591
6 1 4 2	69.050	73.408	-0.509
6 1 4 3	14.050	18.119	-0.956
6 1 4 4	49.050	46.821	0.326
6 1 4 5	31.050	40.097	-1.429
6 1 5 1	42.050	47.059	-0.730
6 1 5 2	55.050	44.738	1.542
6 1 5 3	12.050	13.412	-0.372
6 1 5 4	28.050	26.501	0.301
6 1 5 5	32.050	37.540	-0.896
6 2 1 1	57.050	55.667	0.185
6 2 1 2	40.050	41.493	-0.224
6 2 1 3	0.050	1.678	-1.257
6 2 1 4	11.050	10.308	0.231
6 2 1 5	16.050	15.104	0.243
6 2 2 1	5.050	5.294	-0.106
6 2 2 2	10.050	7.266	1.033
6 2 2 3	0.050	0.181	-0.308
6 2 2 4	1.050	1.840	-0.583
6 2 2 5	1.050	2.669	-0.991
6 2 3 1	6.050	7.371	-0.487
6 2 3 2	4.050	8.157	-1.438
6 2 3 3	3.050	0.490	3.658
6 2 3 4	5.050	2.742	1.394
6 2 3 5	4.050	3.489	0.300
6 2 4 1	20.050	19.328	0.164
6 2 4 2	22.050	26.896	-0.934
6 2 4 3	0.050	0.999	-0.949
6 2 4 4	12.050	11.312	0.219
6 2 4 5	18.050	13.715	1.171
6 2 5 1	12.050	12.590	-0.152
6 2 5 2	28.050	20.437	1.684
6 2 5 3	1.050	0.902	0.156
6 2 5 4	5.050	8.048	-1.057
6 2 5 5	11.050	15.272	-1.080
7 1 1 1	258.050	271.845	-0.837
7 1 1 2	85.050	90.283	-0.551
7 1 1 3	29.050	20.234	1.960
7 1 1 4	35.050	32.576	0.433
7 1 1 5	44.050	36.311	1.284
7 1 2 1	49.050	47.512	0.223

7 1 2 2	42.050	41.808	0.037
7 1 2 3	0.050	4.316	-2.053
7 1 2 4	16.050	15.557	0.125
7 1 2 5	19.050	17.057	0.483
7 1 3 1	18.050	25.717	-1.512
7 1 3 2	0.050	16.048	-3.993
7 1 3 3	37.050	6.754	11.657
7 1 3 4	9.050	9.383	-0.109
7 1 3 5	2.050	8.349	-2.180
7 1 4 1	212.050	192.146	1.436
7 1 4 2	191.050	172.819	1.387
7 1 4 3	19.050	33.785	-2.535
7 1 4 4	128.050	144.477	-1.367
7 1 4 5	112.050	119.023	-0.639
7 1 5 1	95.050	95.030	0.002
7 1 5 2	112.050	109.292	0.264
7 1 5 3	8.050	28.161	-3.790
7 1 5 4	96.050	82.256	1.521
7 1 5 5	142.050	138.511	0.301
7 2 1 1	71.050	74.963	-0.452
7 2 1 2	43.050	40.491	0.402
7 2 1 3	1.050	2.987	-1.121
7 2 1 4	21.050	16.431	1.140
7 2 1 5	13.050	14.378	-0.350
7 2 2 1	13.050	13.688	-0.172
7 2 2 2	13.050	14.626	-0.412
7 2 2 3	0.050	0.627	-0.729
7 2 2 4	8.050	6.064	0.806
7 2 2 5	6.050	5.246	0.351
7 2 3 1	3.050	4.059	-0.501
7 2 3 2	0.050	3.410	-1.819
7 2 3 3	8.050	0.391	12.248
7 2 3 4	0.050	1.940	-1.357
7 2 3 5	0.050	1.450	-1.163
7 2 4 1	56.050	52.176	0.536
7 2 4 2	60.050	56.612	0.457
7 2 4 3	1.050	3.795	-1.409
7 2 4 4	40.050	41.371	-0.205
7 2 4 5	26.050	29.295	-0.600
7 2 5 1	25.050	23.364	0.349
7 2 5 2	29.050	30.111	-0.193
7 2 5 3	0.050	2.449	-1.533
7 2 5 4	17.050	20.444	-0.751
7 2 5 5	29.050	23.881	1.058

*** LOG-LINEAR PARAMETERS ***

* TABLE PCOD [or P(PCOD)] *

effect	beta	exp(beta)
main	3.1435	23.1857
P		
1	1.0990	3.0012
2	0.4774	1.6118
3	0.1244	1.1324
4	-0.1720	0.8420
5	-0.6788	0.5072
6	-0.6742	0.5096
7	-0.1757	0.8389
C		
1	0.4887	1.6301
2	-0.4887	0.6134
O		
1	0.0381	1.0389
2	-0.8285	0.4367
3	-0.2159	0.8058
4	0.3583	1.4309
5	0.6479	1.9116
D		
1	0.5490	1.7316
2	0.4265	1.5319
3	-0.9451	0.3886
4	-0.2038	0.8156
5	0.1734	1.1894
PC		
1 1	-0.3167	0.7285
1 2	0.3167	1.3726
2 1	-0.3231	0.7239
2 2	0.3231	1.3814
3 1	-0.0753	0.9274
3 2	0.0753	1.0783
4 1	-0.0126	0.9875
4 2	0.0126	1.0127
5 1	0.2558	1.2915
5 2	-0.2558	0.7743
6 1	0.2192	1.2450
6 2	-0.2192	0.8032
7 1	0.2528	1.2876
7 2	-0.2528	0.7766
PO		
1 1	-0.8815	0.4142
1 2	0.0170	1.0171
1 3	0.6423	1.9009
1 4	-0.4879	0.6139

1 5	0.7101	2.0341
2 1	-0.6729	0.5102
2 2	0.1165	1.1236
2 3	0.0575	1.0592
2 4	0.0536	1.0551
2 5	0.4452	1.5608
3 1	-0.3991	0.6709
3 2	0.6398	1.8961
3 3	0.2897	1.3361
3 4	-0.2894	0.7487
3 5	-0.2410	0.7858
4 1	0.1699	1.1852
4 2	0.0218	1.0221
4 3	0.3336	1.3960
4 4	-0.4215	0.6561
4 5	-0.1039	0.9013
5 1	0.6449	1.9058
5 2	-0.4765	0.6209
5 3	-0.0266	0.9738
5 4	0.2334	1.2629
5 5	-0.3752	0.6871
6 1	0.6678	1.9500
6 2	-0.4722	0.6236
6 3	-0.0463	0.9547
6 4	0.2271	1.2549
6 5	-0.3763	0.6864
7 1	0.4709	1.6014
7 2	0.1536	1.1660
7 3	-1.2503	0.2864
7 4	0.6846	1.9831
7 5	-0.0588	0.9429
CO		
1 1	-0.1395	0.8698
1 2	-0.0910	0.9130
1 3	0.1554	1.1682
1 4	0.0340	1.0346
1 5	0.0411	1.0419
2 1	0.1395	1.1497
2 2	0.0910	1.0953
2 3	-0.1554	0.8560
2 4	-0.0340	0.9665
2 5	-0.0411	0.9597
PD		
1 1	-0.2638	0.7681
1 2	-0.3386	0.7128
1 3	0.6637	1.9420
1 4	-0.2618	0.7696
1 5	0.2005	1.2220

2 1	-0.1955	0.8224
2 2	-0.3697	0.6910
2 3	0.3877	1.4736
2 4	0.0305	1.0310
2 5	0.1469	1.1583
3 1	-0.1627	0.8498
3 2	-0.2744	0.7600
3 3	0.5376	1.7119
3 4	-0.1007	0.9042
3 5	0.0003	1.0003
4 1	-0.2966	0.7433
4 2	0.0914	1.0958
4 3	0.0297	1.0302
4 4	0.0544	1.0559
4 5	0.1210	1.1286
5 1	0.3197	1.3766
5 2	0.3508	1.4202
5 3	-0.5529	0.5753
5 4	0.0210	1.0212
5 5	-0.1384	0.8707
6 1	0.3193	1.3762
6 2	0.3438	1.4103
6 3	-0.5587	0.5719
6 4	0.0352	1.0358
6 5	-0.1396	0.8697
7 1	0.2797	1.3228
7 2	0.1966	1.2173
7 3	-0.5071	0.6022
7 4	0.2214	1.2479
7 5	-0.1907	0.8264
CD		
1 1	-0.0024	0.9976
1 2	-0.2594	0.7715
1 3	0.4139	1.5128
1 4	-0.1163	0.8902
1 5	-0.0357	0.9649
2 1	0.0024	1.0024
2 2	0.2594	1.2962
2 3	-0.4139	0.6610
2 4	0.1163	1.1234
2 5	0.0357	1.0364
PCO		
1 1 1	-0.0167	0.9835
1 1 2	0.0907	1.0950
1 1 3	-0.2465	0.7816
1 1 4	0.0287	1.0291
1 1 5	0.1437	1.1545
1 2 1	0.0167	1.0168

1 2 2	-0.0907	0.9133
1 2 3	0.2465	1.2795
1 2 4	-0.0287	0.9717
1 2 5	-0.1437	0.8661
2 1 1	0.1519	1.1641
2 1 2	0.0753	1.0782
2 1 3	-0.2034	0.8160
2 1 4	-0.0558	0.9458
2 1 5	0.0319	1.0324
2 2 1	-0.1519	0.8591
2 2 2	-0.0753	0.9275
2 2 3	0.2034	1.2255
2 2 4	0.0558	1.0573
2 2 5	-0.0319	0.9686
3 1 1	0.0496	1.0509
3 1 2	0.2006	1.2221
3 1 3	-0.1584	0.8535
3 1 4	0.0044	1.0044
3 1 5	-0.0962	0.9083
3 2 1	-0.0496	0.9516
3 2 2	-0.2006	0.8182
3 2 3	0.1584	1.1717
3 2 4	-0.0044	0.9956
3 2 5	0.0962	1.1009
4 1 1	-0.0484	0.9527
4 1 2	-0.0542	0.9473
4 1 3	0.1054	1.1111
4 1 4	0.0086	1.0086
4 1 5	-0.0113	0.9888
4 2 1	0.0484	1.0496
4 2 2	0.0542	1.0557
4 2 3	-0.1054	0.9000
4 2 4	-0.0086	0.9915
4 2 5	0.0113	1.0114
5 1 1	-0.0408	0.9600
5 1 2	-0.1422	0.8674
5 1 3	0.2270	1.2549
5 1 4	0.0103	1.0103
5 1 5	-0.0543	0.9471
5 2 1	0.0408	1.0416
5 2 2	0.1422	1.1528
5 2 3	-0.2270	0.7969
5 2 4	-0.0103	0.9898
5 2 5	0.0543	1.0558
6 1 1	-0.0551	0.9463
6 1 2	-0.1544	0.8570
6 1 3	0.2157	1.2407
6 1 4	0.0535	1.0550

6 1 5	-0.0597	0.9421
6 2 1	0.0551	1.0567
6 2 2	0.1544	1.1669
6 2 3	-0.2157	0.8060
6 2 4	-0.0535	0.9479
6 2 5	0.0597	1.0615
7 1 1	-0.0405	0.9603
7 1 2	-0.0159	0.9842
7 1 3	0.0602	1.0621
7 1 4	-0.0496	0.9516
7 1 5	0.0459	1.0469
7 2 1	0.0405	1.0414
7 2 2	0.0159	1.0160
7 2 3	-0.0602	0.9416
7 2 4	0.0496	1.0509
7 2 5	-0.0459	0.9552
PCD		
1 1 1	0.0941	1.0987
1 1 2	-0.0936	0.9106
1 1 3	-0.1754	0.8391
1 1 4	0.0834	1.0870
1 1 5	0.0915	1.0959
1 2 1	-0.0941	0.9102
1 2 2	0.0936	1.0981
1 2 3	0.1754	1.1918
1 2 4	-0.0834	0.9200
1 2 5	-0.0915	0.9125
2 1 1	-0.1257	0.8819
2 1 2	0.0290	1.0294
2 1 3	-0.1271	0.8806
2 1 4	0.0762	1.0791
2 1 5	0.1477	1.1591
2 2 1	0.1257	1.1339
2 2 2	-0.0290	0.9714
2 2 3	0.1271	1.1356
2 2 4	-0.0762	0.9267
2 2 5	-0.1477	0.8627
3 1 1	0.1622	1.1761
3 1 2	0.0234	1.0236
3 1 3	-0.0754	0.9274
3 1 4	-0.1367	0.8723
3 1 5	0.0265	1.0269
3 2 1	-0.1622	0.8503
3 2 2	-0.0234	0.9769
3 2 3	0.0754	1.0783
3 2 4	0.1367	1.1465
3 2 5	-0.0265	0.9739
4 1 1	-0.1349	0.8738

4 1 2	0.0158	1.0159
4 1 3	-0.0523	0.9491
4 1 4	0.0043	1.0043
4 1 5	0.1671	1.1819
4 2 1	0.1349	1.1445
4 2 2	-0.0158	0.9843
4 2 3	0.0523	1.0537
4 2 4	-0.0043	0.9957
4 2 5	-0.1671	0.8461
5 1 1	0.0381	1.0388
5 1 2	-0.0411	0.9597
5 1 3	0.2078	1.2310
5 1 4	-0.0032	0.9968
5 1 5	-0.2016	0.8175
5 2 1	-0.0381	0.9626
5 2 2	0.0411	1.0420
5 2 3	-0.2078	0.8123
5 2 4	0.0032	1.0032
5 2 5	0.2016	1.2233
6 1 1	-0.0033	0.9967
6 1 2	-0.0321	0.9684
6 1 3	0.2458	1.2787
6 1 4	0.0167	1.0168
6 1 5	-0.2271	0.7968
6 2 1	0.0033	1.0033
6 2 2	0.0321	1.0326
6 2 3	-0.2458	0.7821
6 2 4	-0.0167	0.9835
6 2 5	0.2271	1.2550
7 1 1	-0.0305	0.9700
7 1 2	0.0986	1.1036
7 1 3	-0.0234	0.9768
7 1 4	-0.0406	0.9602
7 1 5	-0.0041	0.9959
7 2 1	0.0305	1.0309
7 2 2	-0.0986	0.9061
7 2 3	0.0234	1.0237
7 2 4	0.0406	1.0414
7 2 5	0.0041	1.0041

PC [spe(OD,1a)]

1	1.0000
2	1.2178
3	1.2030
4	1.2509
5	1.1023
6	1.2448
7	0.9550
8	0.8181

9	0.7483
10	0.6733
11	0.7155
12	0.6369
13	1.0164
14	0.7116

spe(OD,1a) [PC]

1	0.7587	2.1354
2	0.0024	1.0024
3	0.0304	1.0308
4	-0.4086	0.6645
5	0.1354	1.1450
6	0.3378	1.4018
7	-0.3969	0.6724
8	-0.0429	0.9580
9	-0.0074	0.9927
10	-0.1431	0.8666
11	0.5048	1.6567
12	-0.0793	0.9238
13	-0.2698	0.7636
14	-0.0459	0.9552
15	-0.1523	0.8587
16	0.3698	1.4474

*** (CONDITIONAL) PROBABILITIES ***

* P(PCOD) *

1 1 1 1	0.0057
1 1 1 2	0.0014
1 1 1 3	0.0018
1 1 1 4	0.0007
1 1 1 5	0.0019
1 1 2 1	0.0037
1 1 2 2	0.0024
1 1 2 3	0.0014
1 1 2 4	0.0013
1 1 2 5	0.0033
1 1 3 1	0.0101
1 1 3 2	0.0046
1 1 3 3	0.0111
1 1 3 4	0.0039
1 1 3 5	0.0081
1 1 4 1	0.0052
1 1 4 2	0.0034
1 1 4 3	0.0038

1 1 4 4	0.0041
1 1 4 5	0.0079
1 1 5 1	0.0183
1 1 5 2	0.0154
1 1 5 3	0.0226
1 1 5 4	0.0166
1 1 5 5	0.0648
1 2 1 1	0.0054
1 2 1 2	0.0028
1 2 1 3	0.0011
1 2 1 4	0.0007
1 2 1 5	0.0015
1 2 2 1	0.0022
1 2 2 2	0.0037
1 2 2 3	0.0006
1 2 2 4	0.0010
1 2 2 5	0.0021
1 2 3 1	0.0071
1 2 3 2	0.0077
1 2 3 3	0.0065
1 2 3 4	0.0035
1 2 3 5	0.0058
1 2 4 1	0.0025
1 2 4 2	0.0043
1 2 4 3	0.0014
1 2 4 4	0.0030
1 2 4 5	0.0045
1 2 5 1	0.0065
1 2 5 2	0.0148
1 2 5 3	0.0069
1 2 5 4	0.0090
1 2 5 5	0.0323
2 1 1 1	0.0045
2 1 1 2	0.0012
2 1 1 3	0.0011
2 1 1 4	0.0007
2 1 1 5	0.0014
2 1 2 1	0.0019
2 1 2 2	0.0016
2 1 2 3	0.0006
2 1 2 4	0.0010
2 1 2 5	0.0019
2 1 3 1	0.0027
2 1 3 2	0.0015
2 1 3 3	0.0030
2 1 3 4	0.0016
2 1 3 5	0.0024
2 1 4 1	0.0036

2 1 4 2	0.0031
2 1 4 3	0.0025
2 1 4 4	0.0049
2 1 4 5	0.0068
2 1 5 1	0.0051
2 1 5 2	0.0060
2 1 5 3	0.0066
2 1 5 4	0.0084
2 1 5 5	0.0268
2 2 1 1	0.0042
2 2 1 2	0.0013
2 2 1 3	0.0004
2 2 1 4	0.0005
2 2 1 5	0.0008
2 2 2 1	0.0018
2 2 2 2	0.0019
2 2 2 3	0.0002
2 2 2 4	0.0008
2 2 2 5	0.0011
2 2 3 1	0.0027
2 2 3 2	0.0019
2 2 3 3	0.0014
2 2 3 4	0.0014
2 2 3 5	0.0015
2 2 4 1	0.0034
2 2 4 2	0.0037
2 2 4 3	0.0010
2 2 4 4	0.0041
2 2 4 5	0.0041
2 2 5 1	0.0040
2 2 5 2	0.0058
2 2 5 3	0.0023
2 2 5 4	0.0057
2 2 5 5	0.0137
3 1 1 1	0.0061
3 1 1 2	0.0014
3 1 1 3	0.0015
3 1 1 4	0.0006
3 1 1 5	0.0012
3 1 2 1	0.0044
3 1 2 2	0.0029
3 1 2 3	0.0013
3 1 2 4	0.0012
3 1 2 5	0.0025
3 1 3 1	0.0044
3 1 3 2	0.0020
3 1 3 3	0.0042
3 1 3 4	0.0013

3 1 3 5	0.0022
3 1 4 1	0.0034
3 1 4 2	0.0023
3 1 4 3	0.0021
3 1 4 4	0.0023
3 1 4 5	0.0035
3 1 5 1	0.0030
3 1 5 2	0.0026
3 1 5 3	0.0032
3 1 5 4	0.0023
3 1 5 5	0.0077
3 2 1 1	0.0026
3 2 1 2	0.0012
3 2 1 3	0.0004
3 2 1 4	0.0005
3 2 1 5	0.0006
3 2 2 1	0.0012
3 2 2 2	0.0017
3 2 2 3	0.0002
3 2 2 4	0.0007
3 2 2 5	0.0009
3 2 3 1	0.0014
3 2 3 2	0.0014
3 2 3 3	0.0010
3 2 3 4	0.0010
3 2 3 5	0.0010
3 2 4 1	0.0010
3 2 4 2	0.0015
3 2 4 3	0.0004
3 2 4 4	0.0016
3 2 4 5	0.0015
3 2 5 1	0.0010
3 2 5 2	0.0020
3 2 5 3	0.0008
3 2 5 4	0.0019
3 2 5 5	0.0042
4 1 1 1	0.0045
4 1 1 2	0.0025
4 1 1 3	0.0011
4 1 1 4	0.0010
4 1 1 5	0.0021
4 1 2 1	0.0009
4 1 2 2	0.0013
4 1 2 3	0.0003
4 1 2 4	0.0005
4 1 2 5	0.0011
4 1 3 1	0.0031
4 1 3 2	0.0032

4 1 3 3	0.0026
4 1 3 4	0.0020
4 1 3 5	0.0033
4 1 4 1	0.0016
4 1 4 2	0.0023
4 1 4 3	0.0009
4 1 4 4	0.0020
4 1 4 5	0.0031
4 1 5 1	0.0021
4 1 5 2	0.0038
4 1 5 3	0.0020
4 1 5 4	0.0030
4 1 5 5	0.0091
4 2 1 1	0.0030
4 2 1 2	0.0023
4 2 1 3	0.0003
4 2 1 4	0.0007
4 2 1 5	0.0009
4 2 2 1	0.0006
4 2 2 2	0.0011
4 2 2 3	0.0001
4 2 2 4	0.0004
4 2 2 5	0.0004
4 2 3 1	0.0009
4 2 3 2	0.0012
4 2 3 3	0.0003
4 2 3 4	0.0006
4 2 3 5	0.0006
4 2 4 1	0.0008
4 2 4 2	0.0014
4 2 4 3	0.0002
4 2 4 4	0.0009
4 2 4 5	0.0008
4 2 5 1	0.0011
4 2 5 2	0.0023
4 2 5 3	0.0003
4 2 5 4	0.0013
4 2 5 5	0.0023
5 1 1 1	0.0108
5 1 1 2	0.0040
5 1 1 3	0.0011
5 1 1 4	0.0013
5 1 1 5	0.0015
5 1 2 1	0.0009
5 1 2 2	0.0007
5 1 2 3	0.0001
5 1 2 4	0.0002
5 1 2 5	0.0003

5 1 3 1	0.0042
5 1 3 2	0.0025
5 1 3 3	0.0010
5 1 3 4	0.0012
5 1 3 5	0.0011
5 1 4 1	0.0057
5 1 4 2	0.0044
5 1 4 3	0.0010
5 1 4 4	0.0027
5 1 4 5	0.0025
5 1 5 1	0.0030
5 1 5 2	0.0028
5 1 5 3	0.0008
5 1 5 4	0.0016
5 1 5 5	0.0025
5 2 1 1	0.0031
5 2 1 2	0.0023
5 2 1 3	0.0001
5 2 1 4	0.0006
5 2 1 5	0.0008
5 2 2 1	0.0003
5 2 2 2	0.0004
5 2 2 3	0.0000
5 2 2 4	0.0001
5 2 2 5	0.0001
5 2 3 1	0.0004
5 2 3 2	0.0005
5 2 3 3	0.0000
5 2 3 4	0.0002
5 2 3 5	0.0002
5 2 4 1	0.0011
5 2 4 2	0.0017
5 2 4 3	0.0001
5 2 4 4	0.0007
5 2 4 5	0.0008
5 2 5 1	0.0007
5 2 5 2	0.0012
5 2 5 3	0.0001
5 2 5 4	0.0005
5 2 5 5	0.0009
6 1 1 1	0.0098
6 1 1 2	0.0039
6 1 1 3	0.0011
6 1 1 4	0.0014
6 1 1 5	0.0015
6 1 2 1	0.0008
6 1 2 2	0.0006
6 1 2 3	0.0001

6 1 2 4	0.0002
6 1 2 5	0.0002
6 1 3 1	0.0038
6 1 3 2	0.0023
6 1 3 3	0.0010
6 1 3 4	0.0012
6 1 3 5	0.0011
6 1 4 1	0.0055
6 1 4 2	0.0044
6 1 4 3	0.0011
6 1 4 4	0.0028
6 1 4 5	0.0024
6 1 5 1	0.0028
6 1 5 2	0.0027
6 1 5 3	0.0008
6 1 5 4	0.0016
6 1 5 5	0.0023
6 2 1 1	0.0034
6 2 1 2	0.0025
6 2 1 3	0.0001
6 2 1 4	0.0006
6 2 1 5	0.0009
6 2 2 1	0.0003
6 2 2 2	0.0004
6 2 2 3	0.0000
6 2 2 4	0.0001
6 2 2 5	0.0002
6 2 3 1	0.0004
6 2 3 2	0.0005
6 2 3 3	0.0000
6 2 3 4	0.0002
6 2 3 5	0.0002
6 2 4 1	0.0012
6 2 4 2	0.0016
6 2 4 3	0.0001
6 2 4 4	0.0007
6 2 4 5	0.0008
6 2 5 1	0.0008
6 2 5 2	0.0012
6 2 5 3	0.0001
6 2 5 4	0.0005
6 2 5 5	0.0009
7 1 1 1	0.0164
7 1 1 2	0.0054
7 1 1 3	0.0012
7 1 1 4	0.0020
7 1 1 5	0.0022
7 1 2 1	0.0029

7 1 2 2	0.0025
7 1 2 3	0.0003
7 1 2 4	0.0009
7 1 2 5	0.0010
7 1 3 1	0.0015
7 1 3 2	0.0010
7 1 3 3	0.0004
7 1 3 4	0.0006
7 1 3 5	0.0005
7 1 4 1	0.0116
7 1 4 2	0.0104
7 1 4 3	0.0020
7 1 4 4	0.0087
7 1 4 5	0.0072
7 1 5 1	0.0057
7 1 5 2	0.0066
7 1 5 3	0.0017
7 1 5 4	0.0050
7 1 5 5	0.0083
7 2 1 1	0.0045
7 2 1 2	0.0024
7 2 1 3	0.0002
7 2 1 4	0.0010
7 2 1 5	0.0009
7 2 2 1	0.0008
7 2 2 2	0.0009
7 2 2 3	0.0000
7 2 2 4	0.0004
7 2 2 5	0.0003
7 2 3 1	0.0002
7 2 3 2	0.0002
7 2 3 3	0.0000
7 2 3 4	0.0001
7 2 3 5	0.0001
7 2 4 1	0.0031
7 2 4 2	0.0034
7 2 4 3	0.0002
7 2 4 4	0.0025
7 2 4 5	0.0018
7 2 5 1	0.0014
7 2 5 2	0.0018
7 2 5 3	0.0001
7 2 5 4	0.0012
7 2 5 5	0.0014

For any clarification or extra data, do not hesitate to contact me. César Augusto Ricardi Morgavi, Department of Social and Legal Science, CUCEA, University of Guadalajara.
cesar.ricardi@cucea.udg.mx
personal email: sociologicalthinktankblog@gmail.com

Cite this data as: Ricardi-Morgavi, C. A. (2026). Comparing Patterns of Intergenerational Class Mobility Using Log-Linear Models: Evidence from Seven Countries, Two Cohorts, and Gendered Stratification. Frontiers special issue.
