# Supplementary material for: Comparing patterns of intergenerational class mobility using log-linear models: evidence from seven countries, two cohorts, and gendered stratification
Source: Front Sociol. 2026 May 1;11:1757240. doi: 10.3389/fsoc.2026.1757240 (PMC13177305; doi:10.3389/fsoc.2026.1757240)
Supplement: Supplementary file 2 [file Data_Sheet_2.pdf]

LEM: log-linear and event history analysis with missing data.
Developed by Jeroen Vermunt (c), Tilburg University, The Netherlands.
Version 1.0 (September 18, 1997).

*** INPUT ***

```
man 4
dim 7 2 5 5
lab P S O D
*  mod {PSO PSD OD}
*  mod {PSO PSD POD}
*  mod {PSO PSD spe(OD,1a,P,b)}
*  mod {PSO PSD SOD}
*  mod {PSO PSD spe(OD,1a,S,b)}
*  mod {PSO PSD POD SOD}
  mod {PSO PSD spe(OD,1a,PS,b)}
add .05
nse
dat[131      24      11      28      21
58      36      18      30      51
166     82      181     85      163
68      51      51      117     141
223     204     293     304     1156
66      44      27      3       28
25      64      11      7       58
129     146     92      36      56
38      81      37      14      68
205     270     229     102     452
97      20      14      11      28
35      33      10      31      24
49      20      48      43      44
78      48      46      136     97
83      82      72      180     453
47      16      25      0       9
14      30      3       7       27
40      44      15      14      16
49      51      26      18      69
68      123     61      41      236
69      16      16      11      11
50      40      19      17      16
62      19      42      18      27
18      22      31      43      52
22      28      49      60      85
71      37      12      9       11
69      32      14      7       20
59      37      20      14      32
24      37      20      15      63
```

26	50	28	28	101
63	26	13	19	36
19	11	5	9	16
31	22	45	31	47
30	38	9	32	32
29	27	17	55	111
61	62	7	6	14
17	20	3	4	7
23	43	11	13	26
10	33	6	14	28
25	74	17	23	76
108	27	11	27	30
6	7	0	4	5
27	6	23	19	19
69	16	7	53	27
31	10	5	28	32
125	73	4	3	15
10	15	1	1	2
37	36	8	3	10
60	75	6	7	23
26	73	6	6	14
110	28	10	28	33
5	8	0	4	5
26	6	21	20	15
70	17	6	54	25
30	10	5	27	30
113	70	4	3	17
8	16	1	1	2
35	34	8	3	9
57	74	8	7	24
24	73	8	6	13
174	31	21	39	34
34	17	0	19	14
17	0	24	6	2
152	41	10	142	71
71	25	2	86	80
155	97	9	17	23
28	38	0	5	11
4	0	21	3	0
116	210	10	26	67
49	116	6	27	91]

*Order of the countries: Mexico, Chile, Uruguay, Spain, Sweden, UK and Germany.
*Order of gender: Male, female.

*** STATISTICS ***

Number of iterations = 161
Converge criterion = 0.0000009488

X-squared = 779.0783 (0.0000)
L-squared = 679.9820 (0.0000)
Cressie-Read = 721.9479 (0.0000)
Dissimilarity index = 0.0593
Degrees of freedom = 195
Log-likelihood = -86408.81858
Number of parameters = 154 (+1)
Sample size = 16592.5
BIC(L-squared) = -1214.7757
AIC(L-squared) = 289.9820
BIC(log-likelihood) = 174314.0099
AIC(log-likelihood) = 173125.6372

WARNING: no information is provided on identification of parameters

*** FREQUENCIES ***

P S O D	observed	estimated	std. res.
1 1 1 1	131.050	117.761	1.225
1 1 1 2	24.050	25.255	-0.240
1 1 1 3	11.050	23.124	-2.511
1 1 1 4	28.050	16.245	2.929
1 1 1 5	21.050	32.864	-2.061
1 1 2 1	58.050	59.280	-0.160
1 1 2 2	36.050	39.682	-0.577
1 1 2 3	18.050	16.511	0.379
1 1 2 4	30.050	26.768	0.634
1 1 2 5	51.050	51.008	0.006
1 1 3 1	166.050	173.439	-0.561
1 1 3 2	82.050	76.063	0.687
1 1 3 3	181.050	189.192	-0.592
1 1 3 4	85.050	90.137	-0.536
1 1 3 5	163.050	148.420	1.201
1 1 4 1	68.050	80.272	-1.364
1 1 4 2	51.050	55.147	-0.552
1 1 4 3	51.050	51.263	-0.030
1 1 4 4	117.050	98.969	1.817
1 1 4 5	141.050	142.598	-0.130
1 1 5 1	223.050	215.498	0.514
1 1 5 2	204.050	201.103	0.208
1 1 5 3	293.050	274.160	1.141
1 1 5 4	304.050	332.130	-1.541

1 1 5 5	1156.050	1157.359	-0.038
1 2 1 1	66.050	64.987	0.132
1 2 1 2	44.050	45.060	-0.151
1 2 1 3	27.050	23.481	0.737
1 2 1 4	3.050	7.734	-1.684
1 2 1 5	28.050	26.987	0.205
1 2 2 1	25.050	43.149	-2.755
1 2 2 2	64.050	58.145	0.774
1 2 2 3	11.050	19.120	-1.845
1 2 2 4	7.050	10.261	-1.002
1 2 2 5	58.050	34.576	3.992
1 2 3 1	129.050	112.209	1.590
1 2 3 2	146.050	118.129	2.569
1 2 3 3	92.050	110.317	-1.739
1 2 3 4	36.050	28.967	1.316
1 2 3 5	56.050	89.627	-3.547
1 2 4 1	38.050	50.282	-1.725
1 2 4 2	81.050	68.792	1.478
1 2 4 3	37.050	36.166	0.147
1 2 4 4	14.050	21.493	-1.605
1 2 4 5	68.050	61.517	0.833
1 2 5 1	205.050	192.623	0.895
1 2 5 2	270.050	315.124	-2.539
1 2 5 3	229.050	207.167	1.520
1 2 5 4	102.050	93.794	0.852
1 2 5 5	452.050	449.542	0.118
2 1 1 1	97.050	98.090	-0.105
2 1 1 2	20.050	19.967	0.019
2 1 1 3	14.050	13.681	0.100
2 1 1 4	11.050	16.864	-1.416
2 1 1 5	28.050	21.648	1.376
2 1 2 1	35.050	40.604	-0.872
2 1 2 2	33.050	28.508	0.851
2 1 2 3	10.050	8.283	0.614
2 1 2 4	31.050	25.356	1.131
2 1 2 5	24.050	30.499	-1.168
2 1 3 1	49.050	53.495	-0.608
2 1 3 2	20.050	23.710	-0.752
2 1 3 3	48.050	48.176	-0.018
2 1 3 4	43.050	38.925	0.661
2 1 3 5	44.050	39.943	0.650
2 1 4 1	78.050	70.600	0.887
2 1 4 2	48.050	50.988	-0.411
2 1 4 3	46.050	35.515	1.768
2 1 4 4	136.050	131.472	0.399
2 1 4 5	97.050	116.675	-1.817
2 1 5 1	83.050	79.461	0.403
2 1 5 2	82.050	80.077	0.220

2 1 5 3	72.050	84.595	-1.364
2 1 5 4	180.050	188.632	-0.625
2 1 5 5	453.050	437.485	0.744
2 2 1 1	47.050	44.312	0.411
2 2 1 2	16.050	24.131	-1.645
2 2 1 3	25.050	9.518	5.035
2 2 1 4	0.050	4.044	-1.986
2 2 1 5	9.050	15.246	-1.587
2 2 2 1	14.050	22.554	-1.791
2 2 2 2	30.050	29.106	0.175
2 2 2 3	3.050	6.314	-1.299
2 2 2 4	7.050	5.057	0.887
2 2 2 5	27.050	18.219	2.069
2 2 3 1	40.050	34.209	0.999
2 2 3 2	44.050	32.038	2.122
2 2 3 3	15.050	26.953	-2.293
2 2 3 4	14.050	8.531	1.889
2 2 3 5	16.050	27.519	-2.186
2 2 4 1	49.050	45.175	0.577
2 2 4 2	51.050	59.454	-1.090
2 2 4 3	26.050	23.721	0.478
2 2 4 4	18.050	21.685	-0.781
2 2 4 5	69.050	63.215	0.734
2 2 5 1	68.050	72.001	-0.466
2 2 5 2	123.050	119.522	0.323
2 2 5 3	61.050	63.745	-0.337
2 2 5 4	41.050	40.933	0.018
2 2 5 5	236.050	233.050	0.197
3 1 1 1	69.050	68.615	0.053
3 1 1 2	16.050	16.166	-0.029
3 1 1 3	16.050	16.202	-0.038
3 1 1 4	11.050	10.305	0.232
3 1 1 5	11.050	11.964	-0.264
3 1 2 1	50.050	46.186	0.569
3 1 2 2	40.050	33.611	1.111
3 1 2 3	19.050	15.419	0.925
3 1 2 4	17.050	22.459	-1.141
3 1 2 5	16.050	24.575	-1.720
3 1 3 1	62.050	43.611	2.792
3 1 3 2	19.050	20.873	-0.399
3 1 3 3	42.050	56.312	-1.900
3 1 3 4	18.050	24.376	-1.281
3 1 3 5	27.050	23.078	0.827
3 1 4 1	18.050	33.838	-2.714
3 1 4 2	22.050	25.267	-0.640
3 1 4 3	31.050	25.706	1.054
3 1 4 4	43.050	44.516	-0.220
3 1 4 5	52.050	36.924	2.489

3 1 5 1	22.050	29.001	-1.291
3 1 5 2	28.050	29.333	-0.237
3 1 5 3	49.050	43.612	0.823
3 1 5 4	60.050	47.595	1.805
3 1 5 5	85.050	94.709	-0.993
3 2 1 1	71.050	74.948	-0.450
3 2 1 2	37.050	28.484	1.605
3 2 1 3	12.050	11.496	0.163
3 2 1 4	9.050	6.497	1.002
3 2 1 5	11.050	18.825	-1.792
3 2 2 1	69.050	47.114	3.196
3 2 2 2	32.050	45.220	-1.959
3 2 2 3	14.050	9.605	1.434
3 2 2 4	7.050	10.721	-1.121
3 2 2 5	20.050	29.590	-1.754
3 2 3 1	59.050	51.022	1.124
3 2 3 2	37.050	34.711	0.397
3 2 3 3	20.050	31.598	-2.054
3 2 3 4	14.050	13.017	0.286
3 2 3 5	32.050	31.901	0.026
3 2 4 1	24.050	39.524	-2.461
3 2 4 2	37.050	38.745	-0.272
3 2 4 3	20.050	15.832	1.060
3 2 4 4	15.050	20.370	-1.179
3 2 4 5	63.050	44.780	2.730
3 2 5 1	26.050	36.642	-1.750
3 2 5 2	50.050	46.089	0.583
3 2 5 3	28.050	25.719	0.460
3 2 5 4	28.050	22.646	1.136
3 2 5 5	101.050	102.154	-0.109
4 1 1 1	63.050	65.514	-0.304
4 1 1 2	26.050	26.710	-0.128
4 1 1 3	13.050	14.948	-0.491
4 1 1 4	19.050	19.647	-0.135
4 1 1 5	36.050	30.431	1.019
4 1 2 1	19.050	15.017	1.041
4 1 2 2	11.050	14.115	-0.816
4 1 2 3	5.050	4.428	0.295
4 1 2 4	9.050	10.751	-0.519
4 1 2 5	16.050	15.938	0.028
4 1 3 1	31.050	40.583	-1.496
4 1 3 2	22.050	27.968	-1.119
4 1 3 3	45.050	32.589	2.183
4 1 3 4	31.050	32.211	-0.205
4 1 3 5	47.050	42.897	0.634
4 1 4 1	30.050	24.342	1.157
4 1 4 2	38.050	23.319	3.051
4 1 4 3	9.050	13.196	-1.141

4 1 4 4	32.050	36.419	-0.724
4 1 4 5	32.050	43.974	-1.798
4 1 5 1	29.050	26.794	0.436
4 1 5 2	27.050	32.137	-0.897
4 1 5 3	17.050	24.087	-1.434
4 1 5 4	55.050	47.222	1.139
4 1 5 5	111.050	109.010	0.195
4 2 1 1	61.050	58.784	0.296
4 2 1 2	62.050	55.230	0.918
4 2 1 3	7.050	8.179	-0.395
4 2 1 4	6.050	8.701	-0.899
4 2 1 5	14.050	19.355	-1.206
4 2 2 1	17.050	11.064	1.799
4 2 2 2	20.050	25.264	-1.037
4 2 2 3	3.050	2.022	0.723
4 2 2 4	4.050	4.131	-0.040
4 2 2 5	7.050	8.769	-0.581
4 2 3 1	23.050	26.555	-0.680
4 2 3 2	43.050	43.595	-0.083
4 2 3 3	11.050	14.079	-0.807
4 2 3 4	13.050	11.063	0.597
4 2 3 5	26.050	20.957	1.113
4 2 4 1	10.050	15.488	-1.382
4 2 4 2	33.050	36.087	-0.506
4 2 4 3	6.050	5.408	0.276
4 2 4 4	14.050	12.659	0.391
4 2 4 5	28.050	21.609	1.386
4 2 5 1	25.050	24.358	0.140
4 2 5 2	74.050	72.074	0.233
4 2 5 3	17.050	14.561	0.652
4 2 5 4	23.050	23.696	-0.133
4 2 5 5	76.050	80.561	-0.503
5 1 1 1	108.050	112.835	-0.450
5 1 1 2	27.050	21.323	1.240
5 1 1 3	11.050	13.031	-0.549
5 1 1 4	27.050	29.962	-0.532
5 1 1 5	30.050	26.099	0.773
5 1 2 1	6.050	9.140	-1.022
5 1 2 2	7.050	3.234	2.122
5 1 2 3	0.050	1.280	-1.087
5 1 2 4	4.050	4.665	-0.285
5 1 2 5	5.050	3.932	0.564
5 1 3 1	27.050	36.850	-1.614
5 1 3 2	6.050	10.328	-1.331
5 1 3 3	23.050	10.947	3.658
5 1 3 4	19.050	20.321	-0.282
5 1 3 5	19.050	15.804	0.817
5 1 4 1	69.050	55.402	1.834

5 1 4 2	16.050	19.884	-0.860
5 1 4 3	7.050	12.254	-1.487
5 1 4 4	53.050	49.177	0.552
5 1 4 5	27.050	35.532	-1.423
5 1 5 1	31.050	27.023	0.775
5 1 5 2	10.050	11.482	-0.422
5 1 5 3	5.050	8.738	-1.248
5 1 5 4	28.050	27.125	0.178
5 1 5 5	32.050	31.883	0.030
5 2 1 1	125.050	119.765	0.483
5 2 1 2	73.050	78.718	-0.639
5 2 1 3	4.050	6.175	-0.855
5 2 1 4	3.050	3.779	-0.375
5 2 1 5	15.050	11.812	0.942
5 2 2 1	10.050	10.673	-0.191
5 2 2 2	15.050	14.922	0.033
5 2 2 3	1.050	0.694	0.427
5 2 2 4	1.050	0.739	0.361
5 2 2 5	2.050	2.221	-0.115
5 2 3 1	37.050	37.695	-0.105
5 2 3 2	36.050	39.814	-0.597
5 2 3 3	8.050	6.059	0.809
5 2 3 4	3.050	2.866	0.108
5 2 3 5	10.050	7.815	0.799
5 2 4 1	60.050	56.962	0.409
5 2 4 2	75.050	81.019	-0.663
5 2 4 3	6.050	6.420	-0.146
5 2 4 4	7.050	7.681	-0.228
5 2 4 5	23.050	19.168	0.887
5 2 5 1	26.050	33.154	-1.234
5 2 5 2	73.050	57.776	2.009
5 2 5 3	6.050	5.902	0.061
5 2 5 4	6.050	5.184	0.380
5 2 5 5	14.050	23.234	-1.905
6 1 1 1	110.050	115.118	-0.472
6 1 1 2	28.050	23.146	1.019
6 1 1 3	10.050	12.506	-0.694
6 1 1 4	28.050	32.109	-0.716
6 1 1 5	33.050	26.371	1.301
6 1 2 1	5.050	9.114	-1.346
6 1 2 2	8.050	3.360	2.559
6 1 2 3	0.050	1.193	-1.046
6 1 2 4	4.050	4.781	-0.334
6 1 2 5	5.050	3.803	0.639
6 1 3 1	26.050	34.656	-1.462
6 1 3 2	6.050	10.199	-1.299
6 1 3 3	21.050	9.384	3.808
6 1 3 4	20.050	19.591	0.104

6 1 3 5	15.050	14.420	0.166
6 1 4 1	70.050	55.799	1.908
6 1 4 2	17.050	20.857	-0.834
6 1 4 3	6.050	11.361	-1.576
6 1 4 4	54.050	49.975	0.576
6 1 4 5	25.050	34.258	-1.573
6 1 5 1	30.050	26.563	0.677
6 1 5 2	10.050	11.688	-0.479
6 1 5 3	5.050	7.807	-0.987
6 1 5 4	27.050	26.794	0.050
6 1 5 5	30.050	29.398	0.120
6 2 1 1	113.050	107.242	0.561
6 2 1 2	70.050	76.746	-0.764
6 2 1 3	4.050	7.201	-1.174
6 2 1 4	3.050	3.841	-0.404
6 2 1 5	17.050	12.219	1.382
6 2 2 1	8.050	9.908	-0.590
6 2 2 2	16.050	14.479	0.413
6 2 2 3	1.050	0.828	0.244
6 2 2 4	1.050	0.747	0.351
6 2 2 5	2.050	2.288	-0.157
6 2 3 1	35.050	33.934	0.192
6 2 3 2	34.050	38.036	-0.646
6 2 3 3	8.050	6.679	0.530
6 2 3 4	3.050	2.793	0.154
6 2 3 5	9.050	7.808	0.444
6 2 4 1	57.050	54.370	0.364
6 2 4 2	74.050	80.757	-0.746
6 2 4 3	8.050	7.649	0.145
6 2 4 4	7.050	7.693	-0.232
6 2 4 5	24.050	19.780	0.960
6 2 5 1	24.050	31.796	-1.374
6 2 5 2	73.050	57.232	2.091
6 2 5 3	8.050	6.893	0.441
6 2 5 4	6.050	5.175	0.384
6 2 5 5	13.050	23.154	-2.100
7 1 1 1	174.050	188.228	-1.033
7 1 1 2	31.050	27.161	0.746
7 1 1 3	21.050	12.644	2.364
7 1 1 4	39.050	42.425	-0.518
7 1 1 5	34.050	28.792	0.980
7 1 2 1	34.050	37.444	-0.555
7 1 2 2	17.050	11.895	1.495
7 1 2 3	0.050	3.205	-1.762
7 1 2 4	19.050	19.203	-0.035
7 1 2 5	14.050	12.503	0.438
7 1 3 1	17.050	20.909	-0.844
7 1 3 2	0.050	4.954	-2.203

7 1 3 3	24.050	4.611	9.052
7 1 3 4	6.050	11.821	-1.678
7 1 3 5	2.050	6.955	-1.860
7 1 4 1	152.050	136.182	1.360
7 1 4 2	41.050	44.047	-0.452
7 1 4 3	10.050	20.721	-2.344
7 1 4 4	142.050	140.135	0.162
7 1 4 5	71.050	75.165	-0.475
7 1 5 1	71.050	65.488	0.687
7 1 5 2	25.050	26.193	-0.223
7 1 5 3	2.050	16.069	-3.497
7 1 5 4	86.050	78.666	0.833
7 1 5 5	80.050	77.835	0.251
7 2 1 1	155.050	159.718	-0.369
7 2 1 2	97.050	102.035	-0.494
7 2 1 3	9.050	9.332	-0.092
7 2 1 4	17.050	8.955	2.705
7 2 1 5	23.050	21.210	0.400
7 2 2 1	28.050	24.845	0.643
7 2 2 2	38.050	42.738	-0.717
7 2 2 3	0.050	1.968	-1.367
7 2 2 4	5.050	3.909	0.577
7 2 2 5	11.050	8.790	0.762
7 2 3 1	4.050	9.479	-1.763
7 2 3 2	0.050	11.285	-3.344
7 2 3 3	21.050	2.463	11.844
7 2 3 4	3.050	1.686	1.051
7 2 3 5	0.050	3.337	-1.799
7 2 4 1	116.050	108.276	0.747
7 2 4 2	210.050	190.505	1.416
7 2 4 3	10.050	17.655	-1.810
7 2 4 4	26.050	40.833	-2.313
7 2 4 5	67.050	71.981	-0.581
7 2 5 1	49.050	49.932	-0.125
7 2 5 2	116.050	114.687	0.127
7 2 5 3	6.050	14.832	-2.280
7 2 5 4	27.050	22.867	0.875
7 2 5 5	91.050	86.932	0.442

*** LOG-LINEAR PARAMETERS ***

* TABLE PSOD [or P(PSOD)] *

effect	beta	exp(beta)
main	3.2261	25.1821
P		

1	1.0055	2.7332
2	0.3502	1.4193
3	0.1223	1.1301
4	-0.1701	0.8436
5	-0.6078	0.5445
6	-0.6108	0.5429
7	-0.0893	0.9146
S		
1	0.1558	1.1686
2	-0.1558	0.8557
O		
1	-0.0277	0.9727
2	-0.8749	0.4169
3	-0.1183	0.8884
4	0.3619	1.4361
5	0.6590	1.9328
D		
1	0.6019	1.8256
2	0.2951	1.3433
3	-0.6539	0.5200
4	-0.4175	0.6587
5	0.1744	1.1905
PS		
1 1	0.0315	1.0320
1 2	-0.0315	0.9689
2 1	0.1158	1.1228
2 2	-0.1158	0.8906
3 1	-0.1233	0.8840
3 2	0.1233	1.1313
4 1	0.0317	1.0322
4 2	-0.0317	0.9688
5 1	-0.0018	0.9982
5 2	0.0018	1.0018
6 1	-0.0191	0.9811
6 2	0.0191	1.0193
7 1	-0.0349	0.9657
7 2	0.0349	1.0355
PO		
1 1	-0.8141	0.4430
1 2	0.0869	1.0908
1 3	0.5156	1.6746
1 4	-0.5124	0.5990
1 5	0.7241	2.0628
2 1	-0.6041	0.5466
2 2	0.1657	1.1803
2 3	-0.0459	0.9552
2 4	0.0300	1.0304
2 5	0.4542	1.5750

3 1	-0.3682	0.6920
3 2	0.7347	2.0849
3 3	0.1837	1.2017
3 4	-0.2789	0.7566
3 5	-0.2714	0.7623
4 1	0.1595	1.1729
4 2	0.0103	1.0104
4 3	0.3517	1.4215
4 4	-0.4254	0.6535
4 5	-0.0961	0.9084
5 1	0.5879	1.8002
5 2	-0.5691	0.5660
5 3	0.1331	1.1423
5 4	0.2462	1.2792
5 5	-0.3981	0.6716
6 1	0.6117	1.8437
6 2	-0.5593	0.5716
6 3	0.0924	1.0968
6 4	0.2609	1.2981
6 5	-0.4058	0.6664
7 1	0.4272	1.5330
7 2	0.1307	1.1396
7 3	-1.2306	0.2921
7 4	0.6796	1.9732
7 5	-0.0069	0.9931
SO		
1 1	-0.0101	0.9900
1 2	0.0008	1.0008
1 3	0.0496	1.0509
1 4	0.0245	1.0248
1 5	-0.0648	0.9372
2 1	0.0101	1.0101
2 2	-0.0008	0.9992
2 3	-0.0496	0.9516
2 4	-0.0245	0.9758
2 5	0.0648	1.0670
PD		
1 1	-0.2732	0.7609
1 2	-0.2086	0.8118
1 3	0.4693	1.5989
1 4	-0.1815	0.8340
1 5	0.1940	1.2141
2 1	-0.2354	0.7903
2 2	-0.2000	0.8187
2 3	0.2100	1.2337
2 4	0.0201	1.0203
2 5	0.2053	1.2279
3 1	-0.1398	0.8695

3 2	-0.2296	0.7949
3 3	0.3795	1.4616
3 4	-0.0069	0.9931
3 5	-0.0033	0.9968
4 1	-0.3815	0.6829
4 2	0.1250	1.1332
4 3	-0.0767	0.9262
4 4	0.1579	1.1711
4 5	0.1752	1.1915
5 1	0.3981	1.4890
5 2	0.2068	1.2298
5 3	-0.2872	0.7503
5 4	-0.1096	0.8962
5 5	-0.2082	0.8121
6 1	0.3578	1.4302
6 2	0.2158	1.2408
6 3	-0.2538	0.7758
6 4	-0.1005	0.9044
6 5	-0.2193	0.8031
7 1	0.2740	1.3152
7 2	0.0905	1.0947
7 3	-0.4411	0.6433
7 4	0.2204	1.2466
7 5	-0.1438	0.8661
SD		
1 1	-0.0660	0.9361
1 2	-0.5683	0.5665
1 3	0.0637	1.0657
1 4	0.5277	1.6950
1 5	0.0429	1.0439
2 1	0.0660	1.0682
2 2	0.5683	1.7652
2 3	-0.0637	0.9383
2 4	-0.5277	0.5900
2 5	-0.0429	0.9580
PSO		
1 1 1	-0.0833	0.9200
1 1 2	-0.0745	0.9282
1 1 3	-0.0195	0.9807
1 1 4	0.0845	1.0882
1 1 5	0.0928	1.0973
1 2 1	0.0833	1.0869
1 2 2	0.0745	1.0774
1 2 3	0.0195	1.0197
1 2 4	-0.0845	0.9189
1 2 5	-0.0928	0.9113
2 1 1	0.0131	1.0132
2 1 2	0.0242	1.0245

2 1 3	-0.0595	0.9423
2 1 4	0.0151	1.0152
2 1 5	0.0071	1.0071
2 2 1	-0.0131	0.9870
2 2 2	-0.0242	0.9761
2 2 3	0.0595	1.0613
2 2 4	-0.0151	0.9850
2 2 5	-0.0071	0.9929
3 1 1	-0.0528	0.9486
3 1 2	0.0378	1.0385
3 1 3	-0.0605	0.9413
3 1 4	-0.0078	0.9922
3 1 5	0.0833	1.0869
3 2 1	0.0528	1.0542
3 2 2	-0.0378	0.9629
3 2 3	0.0605	1.0623
3 2 4	0.0078	1.0079
3 2 5	-0.0833	0.9201
4 1 1	-0.0523	0.9491
4 1 2	0.0178	1.0179
4 1 3	0.0233	1.0236
4 1 4	0.0555	1.0571
4 1 5	-0.0444	0.9566
4 2 1	0.0523	1.0536
4 2 2	-0.0178	0.9824
4 2 3	-0.0233	0.9769
4 2 4	-0.0555	0.9460
4 2 5	0.0444	1.0454
5 1 1	0.0805	1.0838
5 1 2	-0.0207	0.9795
5 1 3	-0.0154	0.9848
5 1 4	-0.0097	0.9904
5 1 5	-0.0348	0.9658
5 2 1	-0.0805	0.9226
5 2 2	0.0207	1.0209
5 2 3	0.0154	1.0155
5 2 4	0.0097	1.0097
5 2 5	0.0348	1.0354
6 1 1	0.1050	1.1107
6 1 2	-0.0190	0.9811
6 1 3	-0.0257	0.9746
6 1 4	-0.0124	0.9877
6 1 5	-0.0479	0.9532
6 2 1	-0.1050	0.9003
6 2 2	0.0190	1.0192
6 2 3	0.0257	1.0260
6 2 4	0.0124	1.0124
6 2 5	0.0479	1.0491

7 1 1	-0.0103	0.9897
7 1 2	0.0345	1.0351
7 1 3	0.1572	1.1702
7 1 4	-0.1253	0.8823
7 1 5	-0.0561	0.9454
7 2 1	0.0103	1.0104
7 2 2	-0.0345	0.9660
7 2 3	-0.1572	0.8546
7 2 4	0.1253	1.1334
7 2 5	0.0561	1.0577
PSD		
1 1 1	0.0714	1.0740
1 1 2	0.1738	1.1898
1 1 3	-0.1504	0.8604
1 1 4	-0.1523	0.8588
1 1 5	0.0574	1.0591
1 2 1	-0.0714	0.9311
1 2 2	-0.1738	0.8405
1 2 3	0.1504	1.1622
1 2 4	0.1523	1.1645
1 2 5	-0.0574	0.9442
2 1 1	0.0319	1.0324
2 1 2	0.1902	1.2094
2 1 3	-0.1451	0.8649
2 1 4	-0.0105	0.9896
2 1 5	-0.0664	0.9357
2 2 1	-0.0319	0.9686
2 2 2	-0.1902	0.8268
2 2 3	0.1451	1.1562
2 2 4	0.0105	1.0105
2 2 5	0.0664	1.0687
3 1 1	-0.0319	0.9686
3 1 2	0.3107	1.3644
3 1 3	0.1446	1.1556
3 1 4	-0.2249	0.7986
3 1 5	-0.1985	0.8199
3 2 1	0.0319	1.0324
3 2 2	-0.3107	0.7329
3 2 3	-0.1446	0.8654
3 2 4	0.2249	1.2522
3 2 5	0.1985	1.2196
4 1 1	0.0170	1.0172
4 1 2	0.0811	1.0845
4 1 3	0.1110	1.1174
4 1 4	-0.2566	0.7737
4 1 5	0.0475	1.0486
4 2 1	-0.0170	0.9831
4 2 2	-0.0811	0.9221

4 2 3	-0.1110	0.8950
4 2 4	0.2566	1.2925
4 2 5	-0.0475	0.9536
5 1 1	-0.1349	0.8738
5 1 2	-0.3062	0.7362
5 1 3	0.0813	1.0847
5 1 4	0.2566	1.2925
5 1 5	0.1033	1.1088
5 2 1	0.1349	1.1444
5 2 2	0.3062	1.3583
5 2 3	-0.0813	0.9219
5 2 4	-0.2566	0.7737
5 2 5	-0.1033	0.9019
6 1 1	-0.0852	0.9183
6 1 2	-0.2602	0.7709
6 1 3	-0.0227	0.9776
6 1 4	0.2799	1.3230
6 1 5	0.0882	1.0923
6 2 1	0.0852	1.0890
6 2 2	0.2602	1.2972
6 2 3	0.0227	1.0230
6 2 4	-0.2799	0.7558
6 2 5	-0.0882	0.9155
7 1 1	0.1317	1.1407
7 1 2	-0.1893	0.8275
7 1 3	-0.0187	0.9815
7 1 4	0.1077	1.1137
7 1 5	-0.0314	0.9691
7 2 1	-0.1317	0.8766
7 2 2	0.1893	1.2084
7 2 3	0.0187	1.0189
7 2 4	-0.1077	0.8979
7 2 5	0.0314	1.0319

PS [spe(OD,1a)]

1	1.0000
2	0.5838
3	1.0878
4	0.7580
5	0.9908
6	0.8139
7	0.7338
8	0.7801
9	0.5510
10	0.6631
11	0.5326
12	0.6272
13	0.6933
14	0.8702

spe(OD,1a) [PS]

1	0.9508	2.5879
2	0.0532	1.0546
3	-0.0716	0.9309
4	-0.4723	0.6235
5	0.1909	1.2104
6	0.4315	1.5396
7	-0.4820	0.6176
8	-0.0464	0.9546
9	-0.0246	0.9757
10	-0.2069	0.8131
11	0.6677	1.9498
12	-0.1214	0.8857
13	-0.3261	0.7217
14	-0.0596	0.9422
15	-0.1692	0.8443
16	0.4410	1.5542

*** (CONDITIONAL) PROBABILITIES ***

* P(PSOD) *

1 1 1 1	0.0071
1 1 1 2	0.0015
1 1 1 3	0.0014
1 1 1 4	0.0010
1 1 1 5	0.0020
1 1 2 1	0.0036
1 1 2 2	0.0024
1 1 2 3	0.0010
1 1 2 4	0.0016
1 1 2 5	0.0031
1 1 3 1	0.0105
1 1 3 2	0.0046
1 1 3 3	0.0114
1 1 3 4	0.0054
1 1 3 5	0.0089
1 1 4 1	0.0048
1 1 4 2	0.0033
1 1 4 3	0.0031
1 1 4 4	0.0060
1 1 4 5	0.0086
1 1 5 1	0.0130
1 1 5 2	0.0121
1 1 5 3	0.0165
1 1 5 4	0.0200

1 1 5 5	0.0698
1 2 1 1	0.0039
1 2 1 2	0.0027
1 2 1 3	0.0014
1 2 1 4	0.0005
1 2 1 5	0.0016
1 2 2 1	0.0026
1 2 2 2	0.0035
1 2 2 3	0.0012
1 2 2 4	0.0006
1 2 2 5	0.0021
1 2 3 1	0.0068
1 2 3 2	0.0071
1 2 3 3	0.0066
1 2 3 4	0.0017
1 2 3 5	0.0054
1 2 4 1	0.0030
1 2 4 2	0.0041
1 2 4 3	0.0022
1 2 4 4	0.0013
1 2 4 5	0.0037
1 2 5 1	0.0116
1 2 5 2	0.0190
1 2 5 3	0.0125
1 2 5 4	0.0057
1 2 5 5	0.0271
2 1 1 1	0.0059
2 1 1 2	0.0012
2 1 1 3	0.0008
2 1 1 4	0.0010
2 1 1 5	0.0013
2 1 2 1	0.0024
2 1 2 2	0.0017
2 1 2 3	0.0005
2 1 2 4	0.0015
2 1 2 5	0.0018
2 1 3 1	0.0032
2 1 3 2	0.0014
2 1 3 3	0.0029
2 1 3 4	0.0023
2 1 3 5	0.0024
2 1 4 1	0.0043
2 1 4 2	0.0031
2 1 4 3	0.0021
2 1 4 4	0.0079
2 1 4 5	0.0070
2 1 5 1	0.0048
2 1 5 2	0.0048

2 1 5 3	0.0051
2 1 5 4	0.0114
2 1 5 5	0.0264
2 2 1 1	0.0027
2 2 1 2	0.0015
2 2 1 3	0.0006
2 2 1 4	0.0002
2 2 1 5	0.0009
2 2 2 1	0.0014
2 2 2 2	0.0018
2 2 2 3	0.0004
2 2 2 4	0.0003
2 2 2 5	0.0011
2 2 3 1	0.0021
2 2 3 2	0.0019
2 2 3 3	0.0016
2 2 3 4	0.0005
2 2 3 5	0.0017
2 2 4 1	0.0027
2 2 4 2	0.0036
2 2 4 3	0.0014
2 2 4 4	0.0013
2 2 4 5	0.0038
2 2 5 1	0.0043
2 2 5 2	0.0072
2 2 5 3	0.0038
2 2 5 4	0.0025
2 2 5 5	0.0140
3 1 1 1	0.0041
3 1 1 2	0.0010
3 1 1 3	0.0010
3 1 1 4	0.0006
3 1 1 5	0.0007
3 1 2 1	0.0028
3 1 2 2	0.0020
3 1 2 3	0.0009
3 1 2 4	0.0014
3 1 2 5	0.0015
3 1 3 1	0.0026
3 1 3 2	0.0013
3 1 3 3	0.0034
3 1 3 4	0.0015
3 1 3 5	0.0014
3 1 4 1	0.0020
3 1 4 2	0.0015
3 1 4 3	0.0015
3 1 4 4	0.0027
3 1 4 5	0.0022

3 1 5 1	0.0017
3 1 5 2	0.0018
3 1 5 3	0.0026
3 1 5 4	0.0029
3 1 5 5	0.0057
3 2 1 1	0.0045
3 2 1 2	0.0017
3 2 1 3	0.0007
3 2 1 4	0.0004
3 2 1 5	0.0011
3 2 2 1	0.0028
3 2 2 2	0.0027
3 2 2 3	0.0006
3 2 2 4	0.0006
3 2 2 5	0.0018
3 2 3 1	0.0031
3 2 3 2	0.0021
3 2 3 3	0.0019
3 2 3 4	0.0008
3 2 3 5	0.0019
3 2 4 1	0.0024
3 2 4 2	0.0023
3 2 4 3	0.0010
3 2 4 4	0.0012
3 2 4 5	0.0027
3 2 5 1	0.0022
3 2 5 2	0.0028
3 2 5 3	0.0016
3 2 5 4	0.0014
3 2 5 5	0.0062
4 1 1 1	0.0039
4 1 1 2	0.0016
4 1 1 3	0.0009
4 1 1 4	0.0012
4 1 1 5	0.0018
4 1 2 1	0.0009
4 1 2 2	0.0009
4 1 2 3	0.0003
4 1 2 4	0.0006
4 1 2 5	0.0010
4 1 3 1	0.0024
4 1 3 2	0.0017
4 1 3 3	0.0020
4 1 3 4	0.0019
4 1 3 5	0.0026
4 1 4 1	0.0015
4 1 4 2	0.0014
4 1 4 3	0.0008

4 1 4 4	0.0022
4 1 4 5	0.0027
4 1 5 1	0.0016
4 1 5 2	0.0019
4 1 5 3	0.0015
4 1 5 4	0.0028
4 1 5 5	0.0066
4 2 1 1	0.0035
4 2 1 2	0.0033
4 2 1 3	0.0005
4 2 1 4	0.0005
4 2 1 5	0.0012
4 2 2 1	0.0007
4 2 2 2	0.0015
4 2 2 3	0.0001
4 2 2 4	0.0002
4 2 2 5	0.0005
4 2 3 1	0.0016
4 2 3 2	0.0026
4 2 3 3	0.0008
4 2 3 4	0.0007
4 2 3 5	0.0013
4 2 4 1	0.0009
4 2 4 2	0.0022
4 2 4 3	0.0003
4 2 4 4	0.0008
4 2 4 5	0.0013
4 2 5 1	0.0015
4 2 5 2	0.0043
4 2 5 3	0.0009
4 2 5 4	0.0014
4 2 5 5	0.0049
5 1 1 1	0.0068
5 1 1 2	0.0013
5 1 1 3	0.0008
5 1 1 4	0.0018
5 1 1 5	0.0016
5 1 2 1	0.0006
5 1 2 2	0.0002
5 1 2 3	0.0001
5 1 2 4	0.0003
5 1 2 5	0.0002
5 1 3 1	0.0022
5 1 3 2	0.0006
5 1 3 3	0.0007
5 1 3 4	0.0012
5 1 3 5	0.0010
5 1 4 1	0.0033

5 1 4 2	0.0012
5 1 4 3	0.0007
5 1 4 4	0.0030
5 1 4 5	0.0021
5 1 5 1	0.0016
5 1 5 2	0.0007
5 1 5 3	0.0005
5 1 5 4	0.0016
5 1 5 5	0.0019
5 2 1 1	0.0072
5 2 1 2	0.0047
5 2 1 3	0.0004
5 2 1 4	0.0002
5 2 1 5	0.0007
5 2 2 1	0.0006
5 2 2 2	0.0009
5 2 2 3	0.0000
5 2 2 4	0.0000
5 2 2 5	0.0001
5 2 3 1	0.0023
5 2 3 2	0.0024
5 2 3 3	0.0004
5 2 3 4	0.0002
5 2 3 5	0.0005
5 2 4 1	0.0034
5 2 4 2	0.0049
5 2 4 3	0.0004
5 2 4 4	0.0005
5 2 4 5	0.0012
5 2 5 1	0.0020
5 2 5 2	0.0035
5 2 5 3	0.0004
5 2 5 4	0.0003
5 2 5 5	0.0014
6 1 1 1	0.0069
6 1 1 2	0.0014
6 1 1 3	0.0008
6 1 1 4	0.0019
6 1 1 5	0.0016
6 1 2 1	0.0005
6 1 2 2	0.0002
6 1 2 3	0.0001
6 1 2 4	0.0003
6 1 2 5	0.0002
6 1 3 1	0.0021
6 1 3 2	0.0006
6 1 3 3	0.0006
6 1 3 4	0.0012

6 1 3 5	0.0009
6 1 4 1	0.0034
6 1 4 2	0.0013
6 1 4 3	0.0007
6 1 4 4	0.0030
6 1 4 5	0.0021
6 1 5 1	0.0016
6 1 5 2	0.0007
6 1 5 3	0.0005
6 1 5 4	0.0016
6 1 5 5	0.0018
6 2 1 1	0.0065
6 2 1 2	0.0046
6 2 1 3	0.0004
6 2 1 4	0.0002
6 2 1 5	0.0007
6 2 2 1	0.0006
6 2 2 2	0.0009
6 2 2 3	0.0000
6 2 2 4	0.0000
6 2 2 5	0.0001
6 2 3 1	0.0020
6 2 3 2	0.0023
6 2 3 3	0.0004
6 2 3 4	0.0002
6 2 3 5	0.0005
6 2 4 1	0.0033
6 2 4 2	0.0049
6 2 4 3	0.0005
6 2 4 4	0.0005
6 2 4 5	0.0012
6 2 5 1	0.0019
6 2 5 2	0.0034
6 2 5 3	0.0004
6 2 5 4	0.0003
6 2 5 5	0.0014
7 1 1 1	0.0113
7 1 1 2	0.0016
7 1 1 3	0.0008
7 1 1 4	0.0026
7 1 1 5	0.0017
7 1 2 1	0.0023
7 1 2 2	0.0007
7 1 2 3	0.0002
7 1 2 4	0.0012
7 1 2 5	0.0008
7 1 3 1	0.0013
7 1 3 2	0.0003

7 1 3 3	0.0003
7 1 3 4	0.0007
7 1 3 5	0.0004
7 1 4 1	0.0082
7 1 4 2	0.0027
7 1 4 3	0.0012
7 1 4 4	0.0084
7 1 4 5	0.0045
7 1 5 1	0.0039
7 1 5 2	0.0016
7 1 5 3	0.0010
7 1 5 4	0.0047
7 1 5 5	0.0047
7 2 1 1	0.0096
7 2 1 2	0.0061
7 2 1 3	0.0006
7 2 1 4	0.0005
7 2 1 5	0.0013
7 2 2 1	0.0015
7 2 2 2	0.0026
7 2 2 3	0.0001
7 2 2 4	0.0002
7 2 2 5	0.0005
7 2 3 1	0.0006
7 2 3 2	0.0007
7 2 3 3	0.0001
7 2 3 4	0.0001
7 2 3 5	0.0002
7 2 4 1	0.0065
7 2 4 2	0.0115
7 2 4 3	0.0011
7 2 4 4	0.0025
7 2 4 5	0.0043
7 2 5 1	0.0030
7 2 5 2	0.0069
7 2 5 3	0.0009
7 2 5 4	0.0014
7 2 5 5	0.0052

For any clarification or extra data, do not hesitate to contact me. César Augusto Ricardi Morgavi, Department of Social and Legal Science, CUCEA, University of Guadalajara.
cesar.ricardi@cucea.udg.mx
personal email: sociologicalthinktankblog@gmail.com

Cite this data as: Ricardi-Morgavi, C. A. (2026). Comparing Patterns of Intergenerational Class Mobility Using Log-Linear Models: Evidence from Seven Countries, Two Cohorts, and Gendered Stratification. Frontiers special issue.
