# Supplementary material for: Comparing patterns of intergenerational class mobility using log-linear models: evidence from seven countries, two cohorts, and gendered stratification
Source: Front Sociol. 2026 May 1;11:1757240. doi: 10.3389/fsoc.2026.1757240 (PMC13177305; doi:10.3389/fsoc.2026.1757240)
Supplement: Supplementary file 3 [file Data_Sheet_3.pdf]

LEM: log-linear and event history analysis with missing data.
Developed by Jeroen Vermunt (c), Tilburg University, The Netherlands.
Version 1.0 (September 18, 1997).

*** INPUT ***

```
man 5
dim 7 2 2 5 5
lab P C S O D
* mod {PCSO PCSD OD}
* mod {PCSO PCSD PCOD PSOD CSOD}
 mod {PCSO PCSD spe(OD,1a,PCS,b)}
add .05
nse

dat[71 2       7       15      17
40      13      7       22      31
114     40      120     48      86
51      16      34      60      90
149     111     232     183     749
30      9       12      2       27
22      12      8       6       39
66      36      65      22      30
34      44      33      2       41
136     168     158     82      317
60      22      4       13      4
18      23      11      8       20
52      42      61      37      77
17      35      17      57      51
74      93      61      121     407
36      35      15      1       1
3       52      3       1       19
63      110     27      14      26
4       37      4       12      27
69      102     71      20      135
58      11      12      1       23
22      14      8       14      16
15      15      37      27      15
37      26      23      62      67
52      51      56      107     293
17      4       13      0       8
8       13      3       6       12
28      16      9       13      11
23      20      22      15      52
33      53      47      30      155
39      9       2       10      5
13      19      2       17      8
```

34	5	11	16	29
41	22	23	74	30
31	31	16	73	160
30	12	12	0	1
6	17	0	1	15
12	28	6	1	5
26	31	4	3	17
35	70	14	11	81
46	9	13	5	7
38	23	16	12	14
53	11	33	9	17
16	12	28	28	34
19	18	35	33	52
50	23	11	4	9
53	20	11	3	16
42	24	19	7	20
18	18	15	10	47
18	30	23	17	68
23	7	3	6	4
12	17	3	5	2
9	8	9	9	10
2	10	3	15	18
3	10	14	27	33
21	14	1	5	2
16	12	3	4	4
17	13	1	7	12
6	19	5	5	16
8	20	5	11	33
40	16	11	9	24
14	6	5	7	9
26	16	39	27	40
23	23	8	22	22
24	19	14	35	86
30	38	5	4	9
9	11	2	1	5
18	27	9	10	21
6	22	6	11	23
12	41	15	16	69
23	10	2	10	12
5	5	0	2	7
5	6	6	4	7
7	15	1	10	10
5	8	3	20	25
31	24	2	2	5
8	9	1	3	2
5	16	2	3	5
4	11	0	3	5
13	33	2	7	7

78	12	11	18	23
3	5	0	3	4
24	5	22	16	16
62	6	7	43	18
27	4	5	24	25
103	49	4	2	9
8	8	1	1	2
35	33	6	1	9
48	63	6	5	13
19	52	5	5	11
30	15	0	9	7
3	2	0	1	1
3	1	1	3	3
7	10	0	10	9
4	6	0	4	7
22	24	0	1	6
2	7	0	0	0
2	3	2	2	1
12	12	0	2	10
7	21	1	1	3
77	12	10	18	23
2	6	0	3	4
23	5	20	17	12
63	6	6	44	16
26	4	5	23	22
89	46	4	2	11
6	8	1	1	2
32	31	6	1	8
44	63	8	5	15
16	51	7	5	10
33	16	0	10	10
3	2	0	1	1
3	1	1	3	3
7	11	0	10	9
4	6	0	4	8
24	24	0	1	6
2	8	0	0	0
3	3	2	2	1
13	11	0	2	9
8	22	1	1	3
139	17	20	23	28
30	12	0	13	9
14	0	19	6	2
126	27	9	108	54
62	17	2	76	63
119	68	9	12	16
19	30	0	3	10
4	0	18	3	0

86	164	10	20	58
33	95	6	20	79
35	14	1	16	6
4	5	0	6	5
3	0	5	0	0
26	14	1	34	17
9	8	0	10	17
36	29	0	5	7
9	8	0	2	1
0	0	3	0	0
30	46	0	6	9
16	21	0	7	12

]

*Order of the countries: Mexico, Chile, Uruguay, Spain, Sweden, UK and Germany.
*Order of the cohorts: old 1930-1975; youth 1976-1990.
*Order of gender: Male, female.

*** STATISTICS ***

Number of iterations = 187
Converge criterion = 0.0000009672

X-squared = 1254.3869 (0.0000)
L-squared = 1068.6640 (0.0000)
Cressie-Read = 1128.9889 (0.0000)
Dissimilarity index = 0.0742
Degrees of freedom = 405
Log-likelihood = -96413.14194
Number of parameters = 294 (+1)
Sample size = 16610.0
BIC(L-squared) = -2867.0288
AIC(L-squared) = 258.6640
BIC(log-likelihood) = 195683.3054
AIC(log-likelihood) = 193414.2839

WARNING: no information is provided on identification of parameters

*** FREQUENCIES ***

P C S O D	observed	estimated	std. res.
1 1 1 1 1	71.050	64.834	0.772
1 1 1 1 2	2.050	8.863	-2.288
1 1 1 1 3	7.050	14.594	-1.975
1 1 1 1 4	15.050	7.719	2.639

1 1 1 1 5	17.050	16.241	0.201
1 1 1 2 1	40.050	38.641	0.227
1 1 1 2 2	13.050	17.193	-0.999
1 1 1 2 3	7.050	11.902	-1.406
1 1 1 2 4	22.050	14.709	1.914
1 1 1 2 5	31.050	30.805	0.044
1 1 1 3 1	114.050	111.128	0.277
1 1 1 3 2	40.050	32.572	1.310
1 1 1 3 3	120.050	127.515	-0.661
1 1 1 3 4	48.050	48.558	-0.073
1 1 1 3 5	86.050	88.477	-0.258
1 1 1 4 1	51.050	52.382	-0.184
1 1 1 4 2	16.050	24.311	-1.675
1 1 1 4 3	34.050	35.795	-0.292
1 1 1 4 4	60.050	53.965	0.828
1 1 1 4 5	90.050	84.798	0.570
1 1 1 5 1	149.050	158.266	-0.733
1 1 1 5 2	111.050	99.312	1.178
1 1 1 5 3	232.050	210.444	1.489
1 1 1 5 4	183.050	203.299	-1.420
1 1 1 5 5	749.050	752.929	-0.141
1 1 2 1 1	30.050	30.027	0.004
1 1 2 1 2	9.050	15.560	-1.650
1 1 2 1 3	12.050	14.220	-0.576
1 1 2 1 4	2.050	4.656	-1.208
1 1 2 1 5	27.050	15.787	2.835
1 1 2 2 1	22.050	23.772	-0.353
1 1 2 2 2	12.050	21.895	-2.104
1 1 2 2 3	8.050	13.117	-1.399
1 1 2 2 4	6.050	6.494	-0.174
1 1 2 2 5	39.050	21.971	3.644
1 1 2 3 1	66.050	54.611	1.548
1 1 2 3 2	36.050	41.041	-0.779
1 1 2 3 3	65.050	57.200	1.038
1 1 2 3 4	22.050	15.956	1.526
1 1 2 3 5	30.050	50.442	-2.871
1 1 2 4 1	34.050	34.256	-0.035
1 1 2 4 2	44.050	32.207	2.087
1 1 2 4 3	33.050	27.871	0.981
1 1 2 4 4	2.050	15.202	-3.373
1 1 2 4 5	41.050	44.714	-0.548
1 1 2 5 1	136.050	145.584	-0.790
1 1 2 5 2	168.050	158.547	0.755
1 1 2 5 3	158.050	163.841	-0.452
1 1 2 5 4	82.050	71.942	1.192
1 1 2 5 5	317.050	321.336	-0.239
1 2 1 1 1	60.050	53.435	0.905
1 2 1 1 2	22.050	17.030	1.216

1 2 1 1 3	4.050	8.812	-1.604
1 2 1 1 4	13.050	8.825	1.422
1 2 1 1 5	4.050	15.148	-2.851
1 2 1 2 1	18.050	20.308	-0.501
1 2 1 2 2	23.050	23.246	-0.041
1 2 1 2 3	11.050	4.704	2.926
1 2 1 2 4	8.050	11.815	-1.095
1 2 1 2 5	20.050	20.178	-0.028
1 2 1 3 1	52.050	61.770	-1.237
1 2 1 3 2	42.050	44.983	-0.437
1 2 1 3 3	61.050	59.478	0.204
1 2 1 3 4	37.050	41.730	-0.724
1 2 1 3 5	77.050	61.288	2.013
1 2 1 4 1	17.050	26.862	-1.893
1 2 1 4 2	35.050	32.185	0.505
1 2 1 4 3	17.050	14.752	0.598
1 2 1 4 4	57.050	45.957	1.636
1 2 1 4 5	51.050	57.494	-0.850
1 2 1 5 1	74.050	58.875	1.978
1 2 1 5 2	93.050	97.805	-0.481
1 2 1 5 3	61.050	66.505	-0.669
1 2 1 5 4	121.050	127.923	-0.608
1 2 1 5 5	407.050	405.142	0.095
1 2 2 1 1	36.050	35.078	0.164
1 2 2 1 2	35.050	31.473	0.638
1 2 2 1 3	15.050	8.916	2.054
1 2 2 1 4	1.050	2.801	-1.046
1 2 2 1 5	1.050	9.981	-2.827
1 2 2 2 1	3.050	17.978	-3.521
1 2 2 2 2	52.050	38.935	2.102
1 2 2 2 3	3.050	5.775	-1.134
1 2 2 2 4	1.050	3.419	-1.281
1 2 2 2 5	19.050	12.142	1.983
1 2 2 3 1	63.050	55.500	1.013
1 2 2 3 2	110.050	88.007	2.350
1 2 2 3 3	27.050	47.597	-2.978
1 2 2 3 4	14.050	11.701	0.687
1 2 2 3 5	26.050	37.445	-1.862
1 2 2 4 1	4.050	15.708	-2.942
1 2 2 4 2	37.050	35.107	0.328
1 2 2 4 3	4.050	9.149	-1.686
1 2 2 4 4	12.050	6.283	2.301
1 2 2 4 5	27.050	18.003	2.132
1 2 2 5 1	69.050	50.985	2.530
1 2 2 5 2	102.050	142.728	-3.405
1 2 2 5 3	71.050	48.812	3.183
1 2 2 5 4	20.050	24.045	-0.815
1 2 2 5 5	135.050	130.679	0.382

2 1 1 1 1	58.050	57.244	0.107
2 1 1 1 2	11.050	11.879	-0.241
2 1 1 1 3	12.050	11.598	0.133
2 1 1 1 4	1.050	9.948	-2.821
2 1 1 1 5	23.050	14.581	2.218
2 1 1 2 1	22.050	20.899	0.252
2 1 1 2 2	14.050	15.698	-0.416
2 1 1 2 3	8.050	5.960	0.856
2 1 1 2 4	14.050	12.893	0.322
2 1 1 2 5	16.050	18.800	-0.634
2 1 1 3 1	15.050	25.480	-2.066
2 1 1 3 2	15.050	12.142	0.834
2 1 1 3 3	37.050	30.469	1.192
2 1 1 3 4	27.050	18.270	2.054
2 1 1 3 5	15.050	22.889	-1.639
2 1 1 4 1	37.050	34.499	0.434
2 1 1 4 2	26.050	27.132	-0.208
2 1 1 4 3	23.050	23.449	-0.082
2 1 1 4 4	62.050	63.001	-0.120
2 1 1 4 5	67.050	67.169	-0.015
2 1 1 5 1	52.050	46.128	0.872
2 1 1 5 2	51.050	50.399	0.092
2 1 1 5 3	56.050	64.774	-1.084
2 1 1 5 4	107.050	107.138	-0.009
2 1 1 5 5	293.050	290.811	0.131
2 1 2 1 1	17.050	17.662	-0.146
2 1 2 1 2	4.050	7.546	-1.273
2 1 2 1 3	13.050	5.902	2.942
2 1 2 1 4	0.050	2.709	-1.616
2 1 2 1 5	8.050	8.430	-0.131
2 1 2 2 1	8.050	10.891	-0.861
2 1 2 2 2	13.050	10.921	0.644
2 1 2 2 3	3.050	4.565	-0.709
2 1 2 2 4	6.050	3.870	1.108
2 1 2 2 5	12.050	12.002	0.014
2 1 2 3 1	28.050	17.883	2.404
2 1 2 3 2	16.050	13.261	0.766
2 1 2 3 3	9.050	19.395	-2.349
2 1 2 3 4	13.050	7.021	2.275
2 1 2 3 5	11.050	19.689	-1.947
2 1 2 4 1	23.050	24.728	-0.338
2 1 2 4 2	20.050	25.562	-1.090
2 1 2 4 3	22.050	18.439	0.841
2 1 2 4 4	15.050	18.048	-0.706
2 1 2 4 5	52.050	45.473	0.975
2 1 2 5 1	33.050	38.085	-0.816
2 1 2 5 2	53.050	48.960	0.585
2 1 2 5 3	47.050	45.948	0.163

2 1 2 5 4	30.050	32.601	-0.447
2 1 2 5 5	155.050	152.655	0.194
2 2 1 1 1	39.050	40.928	-0.294
2 2 1 1 2	9.050	6.970	0.788
2 2 1 1 3	2.050	3.613	-0.822
2 2 1 1 4	10.050	6.962	1.170
2 2 1 1 5	5.050	6.778	-0.664
2 2 1 2 1	13.050	19.725	-1.503
2 2 1 2 2	19.050	12.657	1.797
2 2 1 2 3	2.050	2.477	-0.271
2 2 1 2 4	17.050	12.392	1.323
2 2 1 2 5	8.050	12.000	-1.140
2 2 1 3 1	34.050	28.924	0.953
2 2 1 3 2	5.050	11.609	-1.925
2 2 1 3 3	11.050	15.928	-1.222
2 2 1 3 4	16.050	21.218	-1.122
2 2 1 3 5	29.050	17.571	2.738
2 2 1 4 1	41.050	36.219	0.803
2 2 1 4 2	22.050	24.367	-0.469
2 2 1 4 3	23.050	11.139	3.569
2 2 1 4 4	74.050	69.673	0.524
2 2 1 4 5	30.050	48.852	-2.690
2 2 1 5 1	31.050	32.455	-0.247
2 2 1 5 2	31.050	30.648	0.073
2 2 1 5 3	16.050	21.093	-1.098
2 2 1 5 4	73.050	80.005	-0.778
2 2 1 5 5	160.050	147.049	1.072
2 2 2 1 1	30.050	28.011	0.385
2 2 2 1 2	12.050	17.000	-1.200
2 2 2 1 3	12.050	3.396	4.696
2 2 2 1 4	0.050	0.990	-0.945
2 2 2 1 5	1.050	5.853	-1.985
2 2 2 2 1	6.050	11.582	-1.625
2 2 2 2 2	17.050	18.527	-0.343
2 2 2 2 3	0.050	1.816	-1.311
2 2 2 2 4	1.050	1.064	-0.013
2 2 2 2 5	15.050	6.262	3.512
2 2 2 3 1	12.050	16.126	-1.015
2 2 2 3 2	28.050	18.309	2.277
2 2 2 3 3	6.050	7.448	-0.512
2 2 2 3 4	1.050	1.659	-0.473
2 2 2 3 5	5.050	8.709	-1.240
2 2 2 4 1	26.050	19.658	1.442
2 2 2 4 2	31.050	32.554	-0.264
2 2 2 4 3	4.050	5.932	-0.773
2 2 2 4 4	3.050	4.090	-0.514
2 2 2 4 5	17.050	19.015	-0.451
2 2 2 5 1	35.050	33.873	0.202

2 2 2 5 2	70.050	71.861	-0.214
2 2 2 5 3	14.050	17.658	-0.859
2 2 2 5 4	11.050	8.447	0.896
2 2 2 5 5	81.050	79.411	0.184
3 1 1 1 1	46.050	46.430	-0.056
3 1 1 1 2	9.050	8.126	0.324
3 1 1 1 3	13.050	12.600	0.127
3 1 1 1 4	5.050	5.753	-0.293
3 1 1 1 5	7.050	7.341	-0.107
3 1 1 2 1	38.050	38.035	0.002
3 1 1 2 2	23.050	19.917	0.702
3 1 1 2 3	16.050	13.812	0.602
3 1 1 2 4	12.050	13.868	-0.488
3 1 1 2 5	14.050	17.618	-0.850
3 1 1 3 1	53.050	35.582	2.928
3 1 1 3 2	11.050	12.645	-0.448
3 1 1 3 3	33.050	43.816	-1.626
3 1 1 3 4	9.050	14.746	-1.483
3 1 1 3 5	17.050	16.462	0.145
3 1 1 4 1	16.050	28.710	-2.363
3 1 1 4 2	12.050	15.634	-0.906
3 1 1 4 3	28.050	21.849	1.327
3 1 1 4 4	28.050	26.385	0.324
3 1 1 4 5	34.050	25.672	1.654
3 1 1 5 1	19.050	23.493	-0.917
3 1 1 5 2	18.050	16.928	0.273
3 1 1 5 3	35.050	33.173	0.326
3 1 1 5 4	33.050	26.499	1.273
3 1 1 5 5	52.050	57.158	-0.676
3 1 2 1 1	50.050	54.677	-0.626
3 1 2 1 2	23.050	16.054	1.746
3 1 2 1 3	11.050	10.085	0.304
3 1 2 1 4	4.050	3.633	0.219
3 1 2 1 5	9.050	12.802	-1.049
3 1 2 2 1	53.050	36.530	2.733
3 1 2 2 2	20.050	28.898	-1.646
3 1 2 2 3	11.050	8.768	0.771
3 1 2 2 4	3.050	6.442	-1.336
3 1 2 2 5	16.050	22.612	-1.380
3 1 2 3 1	42.050	36.236	0.966
3 1 2 3 2	24.050	20.188	0.859
3 1 2 3 3	19.050	26.246	-1.405
3 1 2 3 4	7.050	7.175	-0.047
3 1 2 3 5	20.050	22.406	-0.498
3 1 2 4 1	18.050	28.358	-1.936
3 1 2 4 2	18.050	23.241	-1.077
3 1 2 4 3	15.050	13.291	0.483
3 1 2 4 4	10.050	11.539	-0.438

3 1 2 4 5	47.050	31.821	2.700
3 1 2 5 1	18.050	25.448	-1.467
3 1 2 5 2	30.050	26.869	0.614
3 1 2 5 3	23.050	20.861	0.479
3 1 2 5 4	17.050	12.462	1.300
3 1 2 5 5	68.050	70.609	-0.305
3 2 1 1 1	23.050	21.866	0.253
3 2 1 1 2	7.050	8.164	-0.390
3 2 1 1 3	3.050	4.207	-0.564
3 2 1 1 4	6.050	4.830	0.555
3 2 1 1 5	4.050	4.183	-0.065
3 2 1 2 1	12.050	8.391	1.263
3 2 1 2 2	17.050	13.709	0.902
3 2 1 2 3	3.050	2.390	0.427
3 2 1 2 4	5.050	7.932	-1.023
3 2 1 2 5	2.050	6.829	-1.829
3 2 1 3 1	9.050	8.410	0.221
3 2 1 3 2	8.050	8.152	-0.036
3 2 1 3 3	9.050	12.408	-0.953
3 2 1 3 4	9.050	9.447	-0.129
3 2 1 3 5	10.050	6.833	1.231
3 2 1 4 1	2.050	5.514	-1.475
3 2 1 4 2	10.050	9.495	0.180
3 2 1 4 3	3.050	4.254	-0.584
3 2 1 4 4	15.050	18.105	-0.718
3 2 1 4 5	18.050	10.882	2.173
3 2 1 5 1	3.050	5.069	-0.897
3 2 1 5 2	10.050	12.730	-0.751
3 2 1 5 3	14.050	8.992	1.687
3 2 1 5 4	27.050	21.935	1.092
3 2 1 5 5	33.050	38.524	-0.882
3 2 2 1 1	21.050	21.083	-0.007
3 2 2 1 2	14.050	11.559	0.733
3 2 2 1 3	1.050	1.989	-0.666
3 2 2 1 4	5.050	2.996	1.187
3 2 2 1 5	2.050	5.623	-1.507
3 2 2 2 1	16.050	10.936	1.546
3 2 2 2 2	12.050	15.569	-0.892
3 2 2 2 3	3.050	1.330	1.492
3 2 2 2 4	4.050	3.978	0.036
3 2 2 2 5	4.050	7.437	-1.242
3 2 2 3 1	17.050	14.549	0.656
3 2 2 3 2	13.050	14.779	-0.450
3 2 2 3 3	1.050	5.122	-1.799
3 2 2 3 4	7.050	5.916	0.466
3 2 2 3 5	12.050	9.883	0.689
3 2 2 4 1	6.050	10.849	-1.457
3 2 2 4 2	19.050	15.981	0.768

3 2 2 4 3	5.050	2.512	1.601
3 2 2 4 4	5.050	8.826	-1.271
3 2 2 4 5	16.050	13.082	0.821
3 2 2 5 1	8.050	10.832	-0.845
3 2 2 5 2	20.050	20.362	-0.069
3 2 2 5 3	5.050	4.297	0.363
3 2 2 5 4	11.050	10.534	0.159
3 2 2 5 5	33.050	31.225	0.327
4 1 1 1 1	40.050	42.515	-0.378
4 1 1 1 2	16.050	14.561	0.390
4 1 1 1 3	11.050	11.559	-0.150
4 1 1 1 4	9.050	11.928	-0.833
4 1 1 1 5	24.050	19.687	0.983
4 1 1 2 1	14.050	10.716	1.018
4 1 1 2 2	6.050	8.565	-0.859
4 1 1 2 3	5.050	3.649	0.733
4 1 1 2 4	7.050	6.927	0.047
4 1 1 2 5	9.050	11.394	-0.694
4 1 1 3 1	26.050	34.934	-1.503
4 1 1 3 2	16.050	20.689	-1.020
4 1 1 3 3	39.050	30.587	1.530
4 1 1 3 4	27.050	24.932	0.424
4 1 1 3 5	40.050	37.108	0.483
4 1 1 4 1	23.050	17.810	1.242
4 1 1 4 2	23.050	14.672	2.187
4 1 1 4 3	8.050	10.748	-0.823
4 1 1 4 4	22.050	23.530	-0.305
4 1 1 4 5	22.050	31.490	-1.682
4 1 1 5 1	24.050	21.275	0.602
4 1 1 5 2	19.050	21.764	-0.582
4 1 1 5 3	14.050	20.707	-1.463
4 1 1 5 4	35.050	32.933	0.369
4 1 1 5 5	86.050	81.571	0.496
4 1 2 1 1	30.050	31.952	-0.337
4 1 2 1 2	38.050	29.225	1.632
4 1 2 1 3	5.050	6.294	-0.496
4 1 2 1 4	4.050	5.257	-0.527
4 1 2 1 5	9.050	13.522	-1.216
4 1 2 2 1	9.050	5.210	1.682
4 1 2 2 2	11.050	13.301	-0.617
4 1 2 2 3	2.050	1.348	0.604
4 1 2 2 4	1.050	2.356	-0.851
4 1 2 2 5	5.050	6.035	-0.401
4 1 2 3 1	18.050	16.225	0.453
4 1 2 3 2	27.050	28.808	-0.328
4 1 2 3 3	9.050	13.176	-1.137
4 1 2 3 4	10.050	8.271	0.619
4 1 2 3 5	21.050	18.770	0.526

4 1 2 4 1	6.050	9.242	-1.050
4 1 2 4 2	22.050	24.474	-0.490
4 1 2 4 3	6.050	4.782	0.580
4 1 2 4 4	11.050	9.933	0.354
4 1 2 4 5	23.050	19.819	0.726
4 1 2 5 1	12.050	12.620	-0.160
4 1 2 5 2	41.050	43.442	-0.363
4 1 2 5 3	15.050	11.650	0.996
4 1 2 5 4	16.050	16.433	-0.094
4 1 2 5 5	69.050	69.105	-0.007
4 2 1 1 1	23.050	22.767	0.059
4 2 1 1 2	10.050	12.507	-0.695
4 2 1 1 3	2.050	3.133	-0.612
4 2 1 1 4	10.050	8.554	0.512
4 2 1 1 5	12.050	10.288	0.549
4 2 1 2 1	5.050	4.160	0.437
4 2 1 2 2	5.050	5.783	-0.305
4 2 1 2 3	0.050	0.733	-0.798
4 2 1 2 4	2.050	3.900	-0.937
4 2 1 2 5	7.050	4.674	1.099
4 2 1 3 1	5.050	6.056	-0.409
4 2 1 3 2	6.050	6.063	-0.005
4 2 1 3 3	6.050	3.003	1.759
4 2 1 3 4	4.050	6.330	-0.906
4 2 1 3 5	7.050	6.798	0.097
4 2 1 4 1	7.050	6.378	0.266
4 2 1 4 2	15.050	9.167	1.943
4 2 1 4 3	1.050	2.103	-0.726
4 2 1 4 4	10.050	13.090	-0.840
4 2 1 4 5	10.050	12.512	-0.696
4 2 1 5 1	5.050	5.888	-0.345
4 2 1 5 2	8.050	10.730	-0.818
4 2 1 5 3	3.050	3.278	-0.126
4 2 1 5 4	20.050	14.376	1.496
4 2 1 5 5	25.050	26.978	-0.371
4 2 2 1 1	31.050	26.727	0.836
4 2 2 1 2	24.050	26.747	-0.522
4 2 2 1 3	2.050	1.927	0.088
4 2 2 1 4	2.050	4.005	-0.977
4 2 2 1 5	5.050	4.843	0.094
4 2 2 2 1	8.050	6.491	0.612
4 2 2 2 2	9.050	12.210	-0.904
4 2 2 2 3	1.050	0.554	0.667
4 2 2 2 4	3.050	1.811	0.921
4 2 2 2 5	2.050	2.184	-0.091
4 2 2 3 1	5.050	9.431	-1.427
4 2 2 3 2	16.050	14.191	0.494
4 2 2 3 3	2.050	1.625	0.334

4 2 2 3 4	3.050	2.833	0.129
4 2 2 3 5	5.050	3.171	1.055
4 2 2 4 1	4.050	5.782	-0.720
4 2 2 4 2	11.050	11.124	-0.022
4 2 2 4 3	0.050	0.755	-0.811
4 2 2 4 4	3.050	2.747	0.183
4 2 2 4 5	5.050	2.841	1.310
4 2 2 5 1	13.050	12.819	0.065
4 2 2 5 2	33.050	28.978	0.756
4 2 2 5 3	2.050	2.389	-0.220
4 2 2 5 4	7.050	6.853	0.075
4 2 2 5 5	7.050	11.211	-1.243
5 1 1 1 1	78.050	81.515	-0.384
5 1 1 1 2	12.050	9.150	0.959
5 1 1 1 3	11.050	11.751	-0.204
5 1 1 1 4	18.050	21.870	-0.817
5 1 1 1 5	23.050	17.964	1.200
5 1 1 2 1	3.050	6.729	-1.418
5 1 1 2 2	5.050	1.390	3.104
5 1 1 2 3	0.050	1.141	-1.021
5 1 1 2 4	3.050	3.292	-0.133
5 1 1 2 5	4.050	2.698	0.823
5 1 1 3 1	24.050	34.302	-1.750
5 1 1 3 2	5.050	5.711	-0.277
5 1 1 3 3	22.050	11.475	3.122
5 1 1 3 4	16.050	18.021	-0.464
5 1 1 3 5	16.050	13.741	0.623
5 1 1 4 1	62.050	47.581	2.098
5 1 1 4 2	6.050	10.046	-1.261
5 1 1 4 3	7.050	12.176	-1.469
5 1 1 4 4	43.050	38.941	0.658
5 1 1 4 5	18.050	27.506	-1.803
5 1 1 5 1	27.050	24.122	0.596
5 1 1 5 2	4.050	5.952	-0.780
5 1 1 5 3	5.050	8.708	-1.240
5 1 1 5 4	24.050	22.126	0.409
5 1 1 5 5	25.050	24.342	0.143
5 1 2 1 1	103.050	98.605	0.448
5 1 2 1 2	49.050	54.073	-0.683
5 1 2 1 3	4.050	5.110	-0.469
5 1 2 1 4	2.050	2.369	-0.208
5 1 2 1 5	9.050	7.093	0.735
5 1 2 2 1	8.050	7.727	0.116
5 1 2 2 2	8.050	10.251	-0.688
5 1 2 2 3	1.050	0.506	0.764
5 1 2 2 4	1.050	0.443	0.911
5 1 2 2 5	2.050	1.322	0.633
5 1 2 3 1	35.050	35.390	-0.057

5 1 2 3 2	33.050	34.351	-0.222
5 1 2 3 3	6.050	6.208	-0.063
5 1 2 3 4	1.050	2.251	-0.800
5 1 2 3 5	9.050	6.050	1.220
5 1 2 4 1	48.050	46.639	0.207
5 1 2 4 2	63.050	63.861	-0.101
5 1 2 4 3	6.050	5.550	0.212
5 1 2 4 4	5.050	5.637	-0.247
5 1 2 4 5	13.050	13.563	-0.139
5 1 2 5 1	19.050	24.890	-1.171
5 1 2 5 2	52.050	42.714	1.428
5 1 2 5 3	5.050	4.875	0.079
5 1 2 5 4	5.050	3.549	0.797
5 1 2 5 5	11.050	16.222	-1.284
5 2 1 1 1	30.050	29.964	0.016
5 2 1 1 2	15.050	14.550	0.131
5 2 1 1 3	0.050	0.507	-0.642
5 2 1 1 4	9.050	8.169	0.308
5 2 1 1 5	7.050	8.061	-0.356
5 2 1 2 1	3.050	2.149	0.614
5 2 1 2 2	2.050	2.408	-0.231
5 2 1 2 3	0.050	0.045	0.022
5 2 1 2 4	1.050	1.335	-0.247
5 2 1 2 5	1.050	1.313	-0.229
5 2 1 3 1	3.050	3.535	-0.258
5 2 1 3 2	1.050	2.947	-1.105
5 2 1 3 3	1.050	0.190	1.977
5 2 1 3 4	3.050	2.421	0.404
5 2 1 3 5	3.050	2.158	0.608
5 2 1 4 1	7.050	8.132	-0.379
5 2 1 4 2	10.050	9.387	0.217
5 2 1 4 3	0.050	0.302	-0.459
5 2 1 4 4	10.050	10.225	-0.055
5 2 1 4 5	9.050	8.205	0.295
5 2 1 5 1	4.050	3.470	0.311
5 2 1 5 2	6.050	4.959	0.490
5 2 1 5 3	0.050	0.206	-0.344
5 2 1 5 4	4.050	5.101	-0.465
5 2 1 5 5	7.050	7.513	-0.169
5 2 2 1 1	22.050	21.467	0.126
5 2 2 1 2	24.050	23.435	0.127
5 2 2 1 3	0.050	1.106	-1.004
5 2 2 1 4	1.050	1.749	-0.529
5 2 2 1 5	6.050	5.493	0.238
5 2 2 2 1	2.050	2.735	-0.414
5 2 2 2 2	7.050	4.864	0.991
5 2 2 2 3	0.050	0.161	-0.276
5 2 2 2 4	0.050	0.360	-0.517

5 2 2 2 5	0.050	1.130	-1.016
5 2 2 3 1	2.050	3.254	-0.668
5 2 2 3 2	3.050	4.870	-0.825
5 2 2 3 3	2.050	0.329	3.001
5 2 2 3 4	2.050	0.454	2.369
5 2 2 3 5	1.050	1.343	-0.253
5 2 2 4 1	12.050	9.950	0.666
5 2 2 4 2	12.050	18.004	-1.403
5 2 2 4 3	0.050	0.812	-0.845
5 2 2 4 4	2.050	1.978	0.051
5 2 2 4 5	10.050	5.506	1.936
5 2 2 5 1	7.050	7.843	-0.283
5 2 2 5 2	21.050	16.077	1.240
5 2 2 5 3	1.050	0.843	0.226
5 2 2 5 4	1.050	1.708	-0.504
5 2 2 5 5	3.050	6.778	-1.432
6 1 1 1 1	77.050	80.600	-0.395
6 1 1 1 2	12.050	9.575	0.800
6 1 1 1 3	10.050	10.956	-0.274
6 1 1 1 4	18.050	22.604	-0.958
6 1 1 1 5	23.050	16.514	1.608
6 1 1 2 1	2.050	6.792	-1.820
6 1 1 2 2	6.050	1.462	3.794
6 1 1 2 3	0.050	1.081	-0.992
6 1 1 2 4	3.050	3.421	-0.201
6 1 1 2 5	4.050	2.493	0.986
6 1 1 3 1	23.050	32.324	-1.631
6 1 1 3 2	5.050	5.639	-0.248
6 1 1 3 3	20.050	9.980	3.188
6 1 1 3 4	17.050	17.450	-0.096
6 1 1 3 5	12.050	11.857	0.056
6 1 1 4 1	63.050	48.070	2.161
6 1 1 4 2	6.050	10.569	-1.390
6 1 1 4 3	6.050	11.431	-1.592
6 1 1 4 4	44.050	39.971	0.645
6 1 1 4 5	16.050	25.209	-1.824
6 1 1 5 1	26.050	23.463	0.534
6 1 1 5 2	4.050	6.005	-0.798
6 1 1 5 3	5.050	7.802	-0.985
6 1 1 5 4	23.050	21.803	0.267
6 1 1 5 5	22.050	21.176	0.190
6 1 2 1 1	89.050	83.967	0.555
6 1 2 1 2	46.050	52.179	-0.848
6 1 2 1 3	4.050	6.078	-0.823
6 1 2 1 4	2.050	2.414	-0.235
6 1 2 1 5	11.050	7.612	1.246
6 1 2 2 1	6.050	6.546	-0.194
6 1 2 2 2	8.050	9.344	-0.423

6 1 2 2 3	1.050	0.591	0.597
6 1 2 2 4	1.050	0.427	0.953
6 1 2 2 5	2.050	1.342	0.612
6 1 2 3 1	32.050	30.467	0.287
6 1 2 3 2	31.050	32.406	-0.238
6 1 2 3 3	6.050	6.949	-0.341
6 1 2 3 4	1.050	2.190	-0.770
6 1 2 3 5	8.050	6.239	0.725
6 1 2 4 1	44.050	43.658	0.059
6 1 2 4 2	63.050	64.198	-0.143
6 1 2 4 3	8.050	6.911	0.433
6 1 2 4 4	5.050	5.743	-0.289
6 1 2 4 5	15.050	14.740	0.081
6 1 2 5 1	16.050	22.612	-1.380
6 1 2 5 2	51.050	41.124	1.548
6 1 2 5 3	7.050	5.722	0.555
6 1 2 5 4	5.050	3.475	0.845
6 1 2 5 5	10.050	16.318	-1.552
6 2 1 1 1	33.050	32.867	0.032
6 2 1 1 2	16.050	16.427	-0.093
6 2 1 1 3	0.050	0.543	-0.669
6 2 1 1 4	10.050	9.278	0.253
6 2 1 1 5	10.050	10.134	-0.027
6 2 1 2 1	3.050	2.105	0.651
6 2 1 2 2	2.050	2.356	-0.199
6 2 1 2 3	0.050	0.043	0.033
6 2 1 2 4	1.050	1.314	-0.231
6 2 1 2 5	1.050	1.431	-0.319
6 2 1 3 1	3.050	3.454	-0.217
6 2 1 3 2	1.050	2.906	-1.089
6 2 1 3 3	1.050	0.174	2.103
6 2 1 3 4	3.050	2.370	0.442
6 2 1 3 5	3.050	2.346	0.460
6 2 1 4 1	7.050	8.238	-0.414
6 2 1 4 2	11.050	9.487	0.507
6 2 1 4 3	0.050	0.291	-0.446
6 2 1 4 4	10.050	10.151	-0.032
6 2 1 4 5	9.050	9.084	-0.011
6 2 1 5 1	4.050	3.585	0.245
6 2 1 5 2	6.050	5.074	0.433
6 2 1 5 3	0.050	0.199	-0.334
6 2 1 5 4	4.050	5.137	-0.479
6 2 1 5 5	8.050	8.255	-0.071
6 2 2 1 1	24.050	23.620	0.088
6 2 2 1 2	24.050	23.603	0.092
6 2 2 1 3	0.050	1.096	-0.999
6 2 2 1 4	1.050	1.744	-0.525
6 2 2 1 5	6.050	5.187	0.379

6 2 2 2 1	2.050	3.261	-0.671
6 2 2 2 2	8.050	5.280	1.206
6 2 2 2 3	0.050	0.172	-0.294
6 2 2 2 4	0.050	0.387	-0.542
6 2 2 2 5	0.050	1.150	-1.026
6 2 2 3 1	3.050	3.835	-0.401
6 2 2 3 2	3.050	5.235	-0.955
6 2 2 3 3	2.050	0.346	2.894
6 2 2 3 4	2.050	0.482	2.259
6 2 2 3 5	1.050	1.351	-0.259
6 2 2 4 1	13.050	10.462	0.800
6 2 2 4 2	11.050	17.231	-1.489
6 2 2 4 3	0.050	0.765	-0.817
6 2 2 4 4	2.050	1.866	0.135
6 2 2 4 5	9.050	4.926	1.858
6 2 2 5 1	8.050	9.071	-0.339
6 2 2 5 2	22.050	16.901	1.252
6 2 2 5 3	1.050	0.870	0.193
6 2 2 5 4	1.050	1.771	-0.542
6 2 2 5 5	3.050	6.637	-1.392
7 1 1 1 1	139.050	150.215	-0.911
7 1 1 1 2	17.050	15.771	0.322
7 1 1 1 3	20.050	10.696	2.860
7 1 1 1 4	23.050	30.492	-1.348
7 1 1 1 5	28.050	20.077	1.779
7 1 1 2 1	30.050	30.556	-0.091
7 1 1 2 2	12.050	7.442	1.689
7 1 1 2 3	0.050	2.721	-1.619
7 1 1 2 4	13.050	14.208	-0.307
7 1 1 2 5	9.050	9.324	-0.090
7 1 1 3 1	14.050	18.529	-1.041
7 1 1 3 2	0.050	3.351	-1.803
7 1 1 3 3	19.050	4.215	7.226
7 1 1 3 4	6.050	9.506	-1.121
7 1 1 3 5	2.050	5.649	-1.514
7 1 1 4 1	126.050	113.394	1.189
7 1 1 4 2	27.050	28.462	-0.265
7 1 1 4 3	9.050	17.823	-2.078
7 1 1 4 4	108.050	107.234	0.079
7 1 1 4 5	54.050	57.337	-0.434
7 1 1 5 1	62.050	58.557	0.457
7 1 1 5 2	17.050	18.224	-0.275
7 1 1 5 3	2.050	14.795	-3.314
7 1 1 5 4	76.050	64.811	1.396
7 1 1 5 5	63.050	63.863	-0.102
7 1 2 1 1	119.050	122.714	-0.331
7 1 2 1 2	68.050	71.816	-0.444
7 1 2 1 3	9.050	8.601	0.153

7 1 2 1 4	12.050	5.881	2.544
7 1 2 1 5	16.050	15.239	0.208
7 1 2 2 1	19.050	17.796	0.297
7 1 2 2 2	30.050	33.196	-0.546
7 1 2 2 3	0.050	1.697	-1.264
7 1 2 2 4	3.050	2.671	0.232
7 1 2 2 5	10.050	6.890	1.204
7 1 2 3 1	4.050	8.032	-1.405
7 1 2 3 2	0.050	9.943	-3.137
7 1 2 3 3	18.050	2.788	9.141
7 1 2 3 4	3.050	1.381	1.421
7 1 2 3 5	0.050	3.106	-1.734
7 1 2 4 1	86.050	78.150	0.894
7 1 2 4 2	164.050	151.939	0.983
7 1 2 4 3	10.050	16.302	-1.548
7 1 2 4 4	20.050	31.188	-1.994
7 1 2 4 5	58.050	60.670	-0.336
7 1 2 5 1	33.050	34.558	-0.257
7 1 2 5 2	95.050	90.356	0.494
7 1 2 5 3	6.050	13.862	-2.098
7 1 2 5 4	20.050	17.129	0.706
7 1 2 5 5	79.050	77.345	0.194
7 2 1 1 1	35.050	37.875	-0.459
7 2 1 1 2	14.050	11.680	0.693
7 2 1 1 3	1.050	2.094	-0.721
7 2 1 1 4	16.050	12.268	1.080
7 2 1 1 5	6.050	8.332	-0.791
7 2 1 2 1	4.050	6.705	-1.025
7 2 1 2 2	5.050	4.776	0.125
7 2 1 2 3	0.050	0.463	-0.607
7 2 1 2 4	6.050	4.953	0.493
7 2 1 2 5	5.050	3.353	0.927
7 2 1 3 1	3.050	2.732	0.192
7 2 1 3 2	0.050	1.447	-1.162
7 2 1 3 3	5.050	0.479	6.600
7 2 1 3 4	0.050	2.226	-1.458
7 2 1 3 5	0.050	1.365	-1.126
7 2 1 4 1	26.050	22.077	0.846
7 2 1 4 2	14.050	16.202	-0.535
7 2 1 4 3	1.050	2.682	-0.997
7 2 1 4 4	34.050	33.044	0.175
7 2 1 4 5	17.050	18.245	-0.280
7 2 1 5 1	9.050	7.861	0.424
7 2 1 5 2	8.050	7.145	0.339
7 2 1 5 3	0.050	1.531	-1.197
7 2 1 5 4	10.050	13.759	-1.000
7 2 1 5 5	17.050	13.955	0.829
7 2 2 1 1	36.050	37.724	-0.272

7 2 2 1 2	29.050	28.612	0.082
7 2 2 1 3	0.050	0.905	-0.899
7 2 2 1 4	5.050	4.149	0.443
7 2 2 1 5	7.050	5.861	0.491
7 2 2 2 1	9.050	7.170	0.702
7 2 2 2 2	8.050	9.566	-0.490
7 2 2 2 3	0.050	0.200	-0.335
7 2 2 2 4	2.050	1.375	0.575
7 2 2 2 5	1.050	1.939	-0.638
7 2 2 3 1	0.050	1.240	-1.069
7 2 2 3 2	0.050	1.355	-1.121
7 2 2 3 3	3.050	0.065	11.719
7 2 2 3 4	0.050	0.254	-0.405
7 2 2 3 5	0.050	0.335	-0.493
7 2 2 4 1	30.050	29.525	0.097
7 2 2 4 2	46.050	40.195	0.923
7 2 2 4 3	0.050	1.205	-1.052
7 2 2 4 4	6.050	9.120	-1.017
7 2 2 4 5	9.050	11.204	-0.644
7 2 2 5 1	16.050	15.591	0.116
7 2 2 5 2	21.050	24.521	-0.701
7 2 2 5 3	0.050	0.875	-0.882
7 2 2 5 4	7.050	5.352	0.734
7 2 2 5 5	12.050	9.911	0.680

*** LOG-LINEAR PARAMETERS ***

* TABLE PCSOD [or P(PCSOD)] *

effect	beta	exp(beta)
main	2.3624	10.6166
P		
1	1.1267	3.0855
2	0.4634	1.5895
3	0.1824	1.2001
4	-0.1460	0.8641
5	-0.7236	0.4850
6	-0.7152	0.4891
7	-0.1877	0.8288
C		
1	0.4832	1.6213
2	-0.4832	0.6168
S		
1	0.1423	1.1529
2	-0.1423	0.8674
O		

1	0.0416	1.0424
2	-0.8195	0.4407
3	-0.2171	0.8049
4	0.3497	1.4186
5	0.6453	1.9066
D		
1	0.6128	1.8456
2	0.4050	1.4993
3	-0.8942	0.4089
4	-0.3461	0.7074
5	0.2225	1.2492
PC		
1 1	-0.2951	0.7444
1 2	0.2951	1.3433
2 1	-0.2678	0.7650
2 2	0.2678	1.3071
3 1	-0.0645	0.9376
3 2	0.0645	1.0666
4 1	-0.0009	0.9991
4 2	0.0009	1.0009
5 1	0.2037	1.2260
5 2	-0.2037	0.8157
6 1	0.1805	1.1978
6 2	-0.1805	0.8348
7 1	0.2441	1.2765
7 2	-0.2441	0.7834
PS		
1 1	0.0574	1.0591
1 2	-0.0574	0.9442
2 1	0.1559	1.1687
2 2	-0.1559	0.8557
3 1	-0.1110	0.8949
3 2	0.1110	1.1174
4 1	0.0348	1.0355
4 2	-0.0348	0.9658
5 1	-0.0729	0.9297
5 2	0.0729	1.0756
6 1	-0.0833	0.9201
6 2	0.0833	1.0869
7 1	0.0190	1.0192
7 2	-0.0190	0.9812
CS		
1 1	0.0130	1.0131
1 2	-0.0130	0.9870
2 1	-0.0130	0.9870
2 2	0.0130	1.0131
PO		
1 1	-0.8620	0.4223

1 2	0.0349	1.0355
1 3	0.6360	1.8888
1 4	-0.5089	0.6012
1 5	0.7000	2.0137
2 1	-0.6832	0.5050
2 2	0.1184	1.1257
2 3	0.0623	1.0643
2 4	0.0416	1.0425
2 5	0.4609	1.5855
3 1	-0.3978	0.6718
3 2	0.6389	1.8945
3 3	0.2877	1.3334
3 4	-0.2841	0.7527
3 5	-0.2447	0.7829
4 1	0.1791	1.1961
4 2	0.0182	1.0184
4 3	0.3330	1.3951
4 4	-0.4328	0.6487
4 5	-0.0975	0.9071
5 1	0.6324	1.8821
5 2	-0.4853	0.6155
5 3	-0.0088	0.9913
5 4	0.2430	1.2751
5 5	-0.3814	0.6829
6 1	0.6566	1.9283
6 2	-0.4820	0.6175
6 3	-0.0268	0.9736
6 4	0.2373	1.2678
6 5	-0.3852	0.6803
7 1	0.4749	1.6078
7 2	0.1569	1.1699
7 3	-1.2834	0.2771
7 4	0.7038	2.0215
7 5	-0.0522	0.9491
CO		
1 1	-0.1505	0.8602
1 2	-0.1016	0.9034
1 3	0.1544	1.1669
1 4	0.0466	1.0477
1 5	0.0511	1.0524
2 1	0.1505	1.1625
2 2	0.1016	1.1069
2 3	-0.1544	0.8569
2 4	-0.0466	0.9545
2 5	-0.0511	0.9502
SO		
1 1	-0.0066	0.9934
1 2	-0.0112	0.9888

1 3	0.0628	1.0648
1 4	0.0373	1.0380
1 5	-0.0823	0.9210
2 1	0.0066	1.0066
2 2	0.0112	1.0113
2 3	-0.0628	0.9391
2 4	-0.0373	0.9634
2 5	0.0823	1.0857
PD		
1 1	-0.2883	0.7495
1 2	-0.2776	0.7576
1 3	0.6670	1.9483
1 4	-0.2486	0.7799
1 5	0.1475	1.1589
2 1	-0.1946	0.8231
2 2	-0.2789	0.7566
2 3	0.4122	1.5101
2 4	-0.1051	0.9002
2 5	0.1665	1.1811
3 1	-0.2131	0.8081
3 2	-0.2466	0.7815
3 3	0.4671	1.5953
3 4	0.0201	1.0203
3 5	-0.0274	0.9729
4 1	-0.3314	0.7179
4 2	0.1187	1.1261
4 3	-0.0069	0.9931
4 4	0.1575	1.1706
4 5	0.0621	1.0640
5 1	0.3746	1.4545
5 2	0.2865	1.3318
5 3	-0.5275	0.5901
5 4	-0.0438	0.9571
5 5	-0.0898	0.9141
6 1	0.3667	1.4429
6 2	0.2886	1.3346
6 3	-0.5196	0.5948
6 4	-0.0423	0.9586
6 5	-0.0934	0.9108
7 1	0.2861	1.3313
7 2	0.1092	1.1154
7 3	-0.4922	0.6113
7 4	0.2621	1.2997
7 5	-0.1653	0.8477
CD		
1 1	0.0036	1.0036
1 2	-0.3051	0.7371
1 3	0.4237	1.5276

1 4	-0.0972	0.9074
1 5	-0.0251	0.9752
2 1	-0.0036	0.9964
2 2	0.3051	1.3567
2 3	-0.4237	0.6546
2 4	0.0972	1.1020
2 5	0.0251	1.0254

SD

1 1	-0.0908	0.9132
1 2	-0.5229	0.5928
1 3	0.0021	1.0021
1 4	0.5373	1.7113
1 5	0.0743	1.0771
2 1	0.0908	1.0951
2 2	0.5229	1.6868
2 3	-0.0021	0.9979
2 4	-0.5373	0.5843
2 5	-0.0743	0.9284

PCS

1 1 1	-0.0311	0.9694
1 1 2	0.0311	1.0316
1 2 1	0.0311	1.0316
1 2 2	-0.0311	0.9694
2 1 1	-0.0395	0.9613
2 1 2	0.0395	1.0403
2 2 1	0.0395	1.0403
2 2 2	-0.0395	0.9613
3 1 1	-0.0133	0.9868
3 1 2	0.0133	1.0134
3 2 1	0.0133	1.0134
3 2 2	-0.0133	0.9868
4 1 1	0.0297	1.0302
4 1 2	-0.0297	0.9707
4 2 1	-0.0297	0.9707
4 2 2	0.0297	1.0302
5 1 1	0.0678	1.0701
5 1 2	-0.0678	0.9345
5 2 1	-0.0678	0.9345
5 2 2	0.0678	1.0701
6 1 1	0.0587	1.0604
6 1 2	-0.0587	0.9430
6 2 1	-0.0587	0.9430
6 2 2	0.0587	1.0604
7 1 1	-0.0723	0.9303
7 1 2	0.0723	1.0749
7 2 1	0.0723	1.0749
7 2 2	-0.0723	0.9303

PCO

1 1 1	-0.0098	0.9902
1 1 2	0.0970	1.1019
1 1 3	-0.2645	0.7676
1 1 4	0.0365	1.0372
1 1 5	0.1408	1.1512
1 2 1	0.0098	1.0099
1 2 2	-0.0970	0.9075
1 2 3	0.2645	1.3028
1 2 4	-0.0365	0.9642
1 2 5	-0.1408	0.8686
2 1 1	0.1256	1.1338
2 1 2	0.0819	1.0853
2 1 3	-0.1859	0.8304
2 1 4	-0.0370	0.9636
2 1 5	0.0155	1.0156
2 2 1	-0.1256	0.8820
2 2 2	-0.0819	0.9214
2 2 3	0.1859	1.2042
2 2 4	0.0370	1.0377
2 2 5	-0.0155	0.9847
3 1 1	0.0570	1.0586
3 1 2	0.2054	1.2280
3 1 3	-0.1562	0.8554
3 1 4	-0.0064	0.9936
3 1 5	-0.0997	0.9051
3 2 1	-0.0570	0.9446
3 2 2	-0.2054	0.8143
3 2 3	0.1562	1.1691
3 2 4	0.0064	1.0064
3 2 5	0.0997	1.1048
4 1 1	-0.0411	0.9597
4 1 2	-0.0585	0.9432
4 1 3	0.0915	1.0958
4 1 4	0.0196	1.0198
4 1 5	-0.0115	0.9886
4 2 1	0.0411	1.0419
4 2 2	0.0585	1.0602
4 2 3	-0.0915	0.9126
4 2 4	-0.0196	0.9806
4 2 5	0.0115	1.0116
5 1 1	-0.0405	0.9603
5 1 2	-0.1485	0.8620
5 1 3	0.2227	1.2495
5 1 4	0.0045	1.0045
5 1 5	-0.0382	0.9626
5 2 1	0.0405	1.0414
5 2 2	0.1485	1.1601
5 2 3	-0.2227	0.8003

5 2 4	-0.0045	0.9955
5 2 5	0.0382	1.0389
6 1 1	-0.0520	0.9493
6 1 2	-0.1531	0.8581
6 1 3	0.2071	1.2302
6 1 4	0.0447	1.0457
6 1 5	-0.0467	0.9543
6 2 1	0.0520	1.0534
6 2 2	0.1531	1.1654
6 2 3	-0.2071	0.8129
6 2 4	-0.0447	0.9563
6 2 5	0.0467	1.0479
7 1 1	-0.0391	0.9617
7 1 2	-0.0242	0.9761
7 1 3	0.0853	1.0890
7 1 4	-0.0618	0.9401
7 1 5	0.0398	1.0406
7 2 1	0.0391	1.0398
7 2 2	0.0242	1.0245
7 2 3	-0.0853	0.9183
7 2 4	0.0618	1.0638
7 2 5	-0.0398	0.9610
PSO		
1 1 1	-0.0868	0.9169
1 1 2	-0.0659	0.9362
1 1 3	-0.0436	0.9574
1 1 4	0.0820	1.0854
1 1 5	0.1143	1.1211
1 2 1	0.0868	1.0907
1 2 2	0.0659	1.0681
1 2 3	0.0436	1.0445
1 2 4	-0.0820	0.9213
1 2 5	-0.1143	0.8920
2 1 1	-0.0016	0.9984
2 1 2	0.0381	1.0389
2 1 3	-0.0627	0.9392
2 1 4	0.0148	1.0149
2 1 5	0.0114	1.0115
2 2 1	0.0016	1.0016
2 2 2	-0.0381	0.9626
2 2 3	0.0627	1.0648
2 2 4	-0.0148	0.9853
2 2 5	-0.0114	0.9887
3 1 1	-0.0296	0.9708
3 1 2	0.0519	1.0533
3 1 3	-0.0801	0.9230
3 1 4	-0.0391	0.9617
3 1 5	0.0969	1.1018

3 2 1	0.0296	1.0301
3 2 2	-0.0519	0.9494
3 2 3	0.0801	1.0834
3 2 4	0.0391	1.0399
3 2 5	-0.0969	0.9076
4 1 1	-0.0471	0.9540
4 1 2	0.0145	1.0146
4 1 3	-0.0424	0.9585
4 1 4	0.0982	1.1032
4 1 5	-0.0233	0.9770
4 2 1	0.0471	1.0482
4 2 2	-0.0145	0.9856
4 2 3	0.0424	1.0433
4 2 4	-0.0982	0.9064
4 2 5	0.0233	1.0236
5 1 1	0.0881	1.0921
5 1 2	-0.0258	0.9745
5 1 3	0.0111	1.0112
5 1 4	-0.0169	0.9832
5 1 5	-0.0565	0.9451
5 2 1	-0.0881	0.9157
5 2 2	0.0258	1.0262
5 2 3	-0.0111	0.9889
5 2 4	0.0169	1.0170
5 2 5	0.0565	1.0581
6 1 1	0.1212	1.1288
6 1 2	-0.0327	0.9678
6 1 3	-0.0142	0.9859
6 1 4	-0.0075	0.9926
6 1 5	-0.0668	0.9354
6 2 1	-0.1212	0.8859
6 2 2	0.0327	1.0333
6 2 3	0.0142	1.0143
6 2 4	0.0075	1.0075
6 2 5	0.0668	1.0691
7 1 1	-0.0443	0.9567
7 1 2	0.0199	1.0201
7 1 3	0.2319	1.2610
7 1 4	-0.1315	0.8768
7 1 5	-0.0761	0.9268
7 2 1	0.0443	1.0452
7 2 2	-0.0199	0.9803
7 2 3	-0.2319	0.7930
7 2 4	0.1315	1.1405
7 2 5	0.0761	1.0790
CSO		
1 1 1	-0.0016	0.9984
1 1 2	0.0308	1.0313

1 1 3	-0.0174	0.9828
1 1 4	-0.0438	0.9571
1 1 5	0.0320	1.0325
1 2 1	0.0016	1.0016
1 2 2	-0.0308	0.9697
1 2 3	0.0174	1.0175
1 2 4	0.0438	1.0448
1 2 5	-0.0320	0.9685
2 1 1	0.0016	1.0016
2 1 2	-0.0308	0.9697
2 1 3	0.0174	1.0175
2 1 4	0.0438	1.0448
2 1 5	-0.0320	0.9685
2 2 1	-0.0016	0.9984
2 2 2	0.0308	1.0313
2 2 3	-0.0174	0.9828
2 2 4	-0.0438	0.9571
2 2 5	0.0320	1.0325
PCD		
1 1 1	0.0592	1.0610
1 1 2	-0.0613	0.9405
1 1 3	-0.2103	0.8104
1 1 4	0.1241	1.1321
1 1 5	0.0884	1.0924
1 2 1	-0.0592	0.9425
1 2 2	0.0613	1.0633
1 2 3	0.2103	1.2340
1 2 4	-0.1241	0.8833
1 2 5	-0.0884	0.9154
2 1 1	-0.1942	0.8235
2 1 2	0.0452	1.0462
2 1 3	-0.1850	0.8311
2 1 4	0.2325	1.2617
2 1 5	0.1015	1.1069
2 2 1	0.1942	1.2143
2 2 2	-0.0452	0.9558
2 2 3	0.1850	1.2032
2 2 4	-0.2325	0.7926
2 2 5	-0.1015	0.9035
3 1 1	0.1665	1.1812
3 1 2	0.0615	1.0634
3 1 3	-0.0702	0.9322
3 1 4	-0.1692	0.8444
3 1 5	0.0114	1.0115
3 2 1	-0.1665	0.8466
3 2 2	-0.0615	0.9404
3 2 3	0.0702	1.0727
3 2 4	0.1692	1.1843

3 2 5	-0.0114	0.9887
4 1 1	-0.1564	0.8552
4 1 2	0.0741	1.0769
4 1 3	-0.0908	0.9132
4 1 4	-0.0121	0.9879
4 1 5	0.1853	1.2035
4 2 1	0.1564	1.1693
4 2 2	-0.0741	0.9286
4 2 3	0.0908	1.0950
4 2 4	0.0121	1.0122
4 2 5	-0.1853	0.8309
5 1 1	0.0979	1.1029
5 1 2	-0.0980	0.9067
5 1 3	0.2496	1.2836
5 1 4	-0.0603	0.9415
5 1 5	-0.1893	0.8275
5 2 1	-0.0979	0.9067
5 2 2	0.0980	1.1029
5 2 3	-0.2496	0.7791
5 2 4	0.0603	1.0621
5 2 5	0.1893	1.2084
6 1 1	0.0490	1.0502
6 1 2	-0.0929	0.9113
6 1 3	0.2949	1.3430
6 1 4	-0.0468	0.9543
6 1 5	-0.2041	0.8154
6 2 1	-0.0490	0.9522
6 2 2	0.0929	1.0974
6 2 3	-0.2949	0.7446
6 2 4	0.0468	1.0479
6 2 5	0.2041	1.2265
7 1 1	-0.0220	0.9782
7 1 2	0.0715	1.0741
7 1 3	0.0118	1.0118
7 1 4	-0.0681	0.9341
7 1 5	0.0069	1.0069
7 2 1	0.0220	1.0222
7 2 2	-0.0715	0.9310
7 2 3	-0.0118	0.9883
7 2 4	0.0681	1.0705
7 2 5	-0.0069	0.9932
PSD		
1 1 1	0.0813	1.0847
1 1 2	0.1206	1.1282
1 1 3	-0.1006	0.9043
1 1 4	-0.1339	0.8747
1 1 5	0.0325	1.0330
1 2 1	-0.0813	0.9219

1 2 2	-0.1206	0.8864
1 2 3	0.1006	1.1058
1 2 4	0.1339	1.1432
1 2 5	-0.0325	0.9680
2 1 1	0.0312	1.0317
2 1 2	0.1221	1.1298
2 1 3	-0.1044	0.9009
2 1 4	0.0615	1.0634
2 1 5	-0.1104	0.8955
2 2 1	-0.0312	0.9693
2 2 2	-0.1221	0.8851
2 2 3	0.1044	1.1100
2 2 4	-0.0615	0.9404
2 2 5	0.1104	1.1167
3 1 1	-0.0615	0.9403
3 1 2	0.2696	1.3095
3 1 3	0.2484	1.2820
3 1 4	-0.2377	0.7884
3 1 5	-0.2188	0.8035
3 2 1	0.0615	1.0635
3 2 2	-0.2696	0.7637
3 2 3	-0.2484	0.7800
3 2 4	0.2377	1.2683
3 2 5	0.2188	1.2446
4 1 1	-0.0251	0.9752
4 1 2	0.0350	1.0356
4 1 3	0.1484	1.1600
4 1 4	-0.2548	0.7750
4 1 5	0.0966	1.1014
4 2 1	0.0251	1.0254
4 2 2	-0.0350	0.9656
4 2 3	-0.1484	0.8621
4 2 4	0.2548	1.2902
4 2 5	-0.0966	0.9080
5 1 1	-0.0392	0.9615
5 1 2	-0.1915	0.8257
5 1 3	-0.1398	0.8695
5 1 4	0.2599	1.2968
5 1 5	0.1106	1.1169
5 2 1	0.0392	1.0400
5 2 2	0.1915	1.2110
5 2 3	0.1398	1.1500
5 2 4	-0.2599	0.7711
5 2 5	-0.1106	0.8953
6 1 1	-0.0277	0.9727
6 1 2	-0.1653	0.8476
6 1 3	-0.2035	0.8158
6 1 4	0.2745	1.3159

6 1 5	0.1220	1.1297
6 2 1	0.0277	1.0280
6 2 2	0.1653	1.1798
6 2 3	0.2035	1.2257
6 2 4	-0.2745	0.7599
6 2 5	-0.1220	0.8852
7 1 1	0.0410	1.0419
7 1 2	-0.1905	0.8266
7 1 3	0.1515	1.1635
7 1 4	0.0304	1.0309
7 1 5	-0.0325	0.9681
7 2 1	-0.0410	0.9598
7 2 2	0.1905	1.2098
7 2 3	-0.1515	0.8595
7 2 4	-0.0304	0.9700
7 2 5	0.0325	1.0330
CSD		
1 1 1	0.0815	1.0849
1 1 2	-0.0869	0.9168
1 1 3	0.0775	1.0806
1 1 4	-0.0243	0.9760
1 1 5	-0.0479	0.9533
1 2 1	-0.0815	0.9217
1 2 2	0.0869	1.0907
1 2 3	-0.0775	0.9255
1 2 4	0.0243	1.0246
1 2 5	0.0479	1.0490
2 1 1	-0.0815	0.9217
2 1 2	0.0869	1.0907
2 1 3	-0.0775	0.9255
2 1 4	0.0243	1.0246
2 1 5	0.0479	1.0490
2 2 1	0.0815	1.0849
2 2 2	-0.0869	0.9168
2 2 3	0.0775	1.0806
2 2 4	-0.0243	0.9760
2 2 5	-0.0479	0.9533
PCSO		
1 1 1 1	-0.0100	0.9900
1 1 1 2	-0.0050	0.9950
1 1 1 3	0.1121	1.1186
1 1 1 4	-0.0270	0.9734
1 1 1 5	-0.0700	0.9324
1 1 2 1	0.0100	1.0101
1 1 2 2	0.0050	1.0050
1 1 2 3	-0.1121	0.8940
1 1 2 4	0.0270	1.0274
1 1 2 5	0.0700	1.0725

1 2 1 1	0.0100	1.0101
1 2 1 2	0.0050	1.0050
1 2 1 3	-0.1121	0.8940
1 2 1 4	0.0270	1.0274
1 2 1 5	0.0700	1.0725
1 2 2 1	-0.0100	0.9900
1 2 2 2	-0.0050	0.9950
1 2 2 3	0.1121	1.1186
1 2 2 4	-0.0270	0.9734
1 2 2 5	-0.0700	0.9324
2 1 1 1	0.1534	1.1658
2 1 1 2	-0.0360	0.9646
2 1 1 3	-0.0719	0.9306
2 1 1 4	-0.0525	0.9488
2 1 1 5	0.0070	1.0071
2 1 2 1	-0.1534	0.8578
2 1 2 2	0.0360	1.0367
2 1 2 3	0.0719	1.0745
2 1 2 4	0.0525	1.0539
2 1 2 5	-0.0070	0.9930
2 2 1 1	-0.1534	0.8578
2 2 1 2	0.0360	1.0367
2 2 1 3	0.0719	1.0745
2 2 1 4	0.0525	1.0539
2 2 1 5	-0.0070	0.9930
2 2 2 1	0.1534	1.1658
2 2 2 2	-0.0360	0.9646
2 2 2 3	-0.0719	0.9306
2 2 2 4	-0.0525	0.9488
2 2 2 5	0.0070	1.0071
3 1 1 1	-0.0651	0.9370
3 1 1 2	-0.0385	0.9622
3 1 1 3	0.0475	1.0487
3 1 1 4	0.0872	1.0911
3 1 1 5	-0.0311	0.9693
3 1 2 1	0.0651	1.0672
3 1 2 2	0.0385	1.0392
3 1 2 3	-0.0475	0.9536
3 1 2 4	-0.0872	0.9165
3 1 2 5	0.0311	1.0316
3 2 1 1	0.0651	1.0672
3 2 1 2	0.0385	1.0392
3 2 1 3	-0.0475	0.9536
3 2 1 4	-0.0872	0.9165
3 2 1 5	0.0311	1.0316
3 2 2 1	-0.0651	0.9370
3 2 2 2	-0.0385	0.9622
3 2 2 3	0.0475	1.0487

3 2 2 4	0.0872	1.0911
3 2 2 5	-0.0311	0.9693
4 1 1 1	-0.0255	0.9748
4 1 1 2	0.0452	1.0462
4 1 1 3	0.0834	1.0870
4 1 1 4	-0.0836	0.9198
4 1 1 5	-0.0196	0.9806
4 1 2 1	0.0255	1.0258
4 1 2 2	-0.0452	0.9558
4 1 2 3	-0.0834	0.9200
4 1 2 4	0.0836	1.0872
4 1 2 5	0.0196	1.0198
4 2 1 1	0.0255	1.0258
4 2 1 2	-0.0452	0.9558
4 2 1 3	-0.0834	0.9200
4 2 1 4	0.0836	1.0872
4 2 1 5	0.0196	1.0198
4 2 2 1	-0.0255	0.9748
4 2 2 2	0.0452	1.0462
4 2 2 3	0.0834	1.0870
4 2 2 4	-0.0836	0.9198
4 2 2 5	-0.0196	0.9806
5 1 1 1	-0.0284	0.9720
5 1 1 2	-0.0045	0.9955
5 1 1 3	-0.0378	0.9629
5 1 1 4	0.0328	1.0334
5 1 1 5	0.0379	1.0387
5 1 2 1	0.0284	1.0288
5 1 2 2	0.0045	1.0045
5 1 2 3	0.0378	1.0386
5 1 2 4	-0.0328	0.9677
5 1 2 5	-0.0379	0.9628
5 2 1 1	0.0284	1.0288
5 2 1 2	0.0045	1.0045
5 2 1 3	0.0378	1.0386
5 2 1 4	-0.0328	0.9677
5 2 1 5	-0.0379	0.9628
5 2 2 1	-0.0284	0.9720
5 2 2 2	-0.0045	0.9955
5 2 2 3	-0.0378	0.9629
5 2 2 4	0.0328	1.0334
5 2 2 5	0.0379	1.0387
6 1 1 1	-0.0573	0.9443
6 1 1 2	0.0312	1.0317
6 1 1 3	-0.0229	0.9774
6 1 1 4	0.0106	1.0106
6 1 1 5	0.0384	1.0392
6 1 2 1	0.0573	1.0590

6 1 2 2	-0.0312	0.9693
6 1 2 3	0.0229	1.0231
6 1 2 4	-0.0106	0.9895
6 1 2 5	-0.0384	0.9623
6 2 1 1	0.0573	1.0590
6 2 1 2	-0.0312	0.9693
6 2 1 3	0.0229	1.0231
6 2 1 4	-0.0106	0.9895
6 2 1 5	-0.0384	0.9623
6 2 2 1	-0.0573	0.9443
6 2 2 2	0.0312	1.0317
6 2 2 3	-0.0229	0.9774
6 2 2 4	0.0106	1.0106
6 2 2 5	0.0384	1.0392
7 1 1 1	0.0329	1.0335
7 1 1 2	0.0076	1.0076
7 1 1 3	-0.1104	0.8955
7 1 1 4	0.0325	1.0331
7 1 1 5	0.0374	1.0381
7 1 2 1	-0.0329	0.9676
7 1 2 2	-0.0076	0.9924
7 1 2 3	0.1104	1.1167
7 1 2 4	-0.0325	0.9680
7 1 2 5	-0.0374	0.9633
7 2 1 1	-0.0329	0.9676
7 2 1 2	-0.0076	0.9924
7 2 1 3	0.1104	1.1167
7 2 1 4	-0.0325	0.9680
7 2 1 5	-0.0374	0.9633
7 2 2 1	0.0329	1.0335
7 2 2 2	0.0076	1.0076
7 2 2 3	-0.1104	0.8955
7 2 2 4	0.0325	1.0331
7 2 2 5	0.0374	1.0381
PCSD		
1 1 1 1	-0.0062	0.9938
1 1 1 2	0.1290	1.1377
1 1 1 3	-0.0375	0.9632
1 1 1 4	-0.0866	0.9170
1 1 1 5	0.0014	1.0014
1 1 2 1	0.0062	1.0063
1 1 2 2	-0.1290	0.8790
1 1 2 3	0.0375	1.0382
1 1 2 4	0.0866	1.0905
1 1 2 5	-0.0014	0.9986
1 2 1 1	0.0062	1.0063
1 2 1 2	-0.1290	0.8790
1 2 1 3	0.0375	1.0382

1 2 1 4	0.0866	1.0905
1 2 1 5	-0.0014	0.9986
1 2 2 1	-0.0062	0.9938
1 2 2 2	0.1290	1.1377
1 2 2 3	-0.0375	0.9632
1 2 2 4	-0.0866	0.9170
1 2 2 5	0.0014	1.0014
2 1 1 1	-0.0229	0.9773
2 1 1 2	0.2977	1.3467
2 1 1 3	-0.0491	0.9521
2 1 1 4	-0.2561	0.7740
2 1 1 5	0.0305	1.0310
2 1 2 1	0.0229	1.0232
2 1 2 2	-0.2977	0.7425
2 1 2 3	0.0491	1.0504
2 1 2 4	0.2561	1.2919
2 1 2 5	-0.0305	0.9700
2 2 1 1	0.0229	1.0232
2 2 1 2	-0.2977	0.7425
2 2 1 3	0.0491	1.0504
2 2 1 4	0.2561	1.2919
2 2 1 5	-0.0305	0.9700
2 2 2 1	-0.0229	0.9773
2 2 2 2	0.2977	1.3467
2 2 2 3	-0.0491	0.9521
2 2 2 4	-0.2561	0.7740
2 2 2 5	0.0305	1.0310
3 1 1 1	0.0189	1.0191
3 1 1 2	0.0711	1.0737
3 1 1 3	-0.1440	0.8659
3 1 1 4	0.0465	1.0476
3 1 1 5	0.0073	1.0074
3 1 2 1	-0.0189	0.9812
3 1 2 2	-0.0711	0.9313
3 1 2 3	0.1440	1.1548
3 1 2 4	-0.0465	0.9545
3 1 2 5	-0.0073	0.9927
3 2 1 1	-0.0189	0.9812
3 2 1 2	-0.0711	0.9313
3 2 1 3	0.1440	1.1548
3 2 1 4	-0.0465	0.9545
3 2 1 5	-0.0073	0.9927
3 2 2 1	0.0189	1.0191
3 2 2 2	0.0711	1.0737
3 2 2 3	-0.1440	0.8659
3 2 2 4	0.0465	1.0476
3 2 2 5	0.0073	1.0074
4 1 1 1	0.1097	1.1160

4 1 1 2	0.0878	1.0918
4 1 1 3	-0.0647	0.9373
4 1 1 4	-0.0222	0.9781
4 1 1 5	-0.1107	0.8952
4 1 2 1	-0.1097	0.8961
4 1 2 2	-0.0878	0.9159
4 1 2 3	0.0647	1.0669
4 1 2 4	0.0222	1.0224
4 1 2 5	0.1107	1.1170
4 2 1 1	-0.1097	0.8961
4 2 1 2	-0.0878	0.9159
4 2 1 3	0.0647	1.0669
4 2 1 4	0.0222	1.0224
4 2 1 5	0.1107	1.1170
4 2 2 1	0.1097	1.1160
4 2 2 2	0.0878	1.0918
4 2 2 3	-0.0647	0.9373
4 2 2 4	-0.0222	0.9781
4 2 2 5	-0.1107	0.8952
5 1 1 1	-0.1388	0.8704
5 1 1 2	-0.2879	0.7498
5 1 1 3	0.2725	1.3132
5 1 1 4	0.0839	1.0875
5 1 1 5	0.0703	1.0729
5 1 2 1	0.1388	1.1489
5 1 2 2	0.2879	1.3337
5 1 2 3	-0.2725	0.7615
5 1 2 4	-0.0839	0.9195
5 1 2 5	-0.0703	0.9321
5 2 1 1	0.1388	1.1489
5 2 1 2	0.2879	1.3337
5 2 1 3	-0.2725	0.7615
5 2 1 4	-0.0839	0.9195
5 2 1 5	-0.0703	0.9321
5 2 2 1	-0.1388	0.8704
5 2 2 2	-0.2879	0.7498
5 2 2 3	0.2725	1.3132
5 2 2 4	0.0839	1.0875
5 2 2 5	0.0703	1.0729
6 1 1 1	-0.0748	0.9279
6 1 1 2	-0.2583	0.7723
6 1 1 3	0.2302	1.2588
6 1 1 4	0.0987	1.1038
6 1 1 5	0.0043	1.0043
6 1 2 1	0.0748	1.0777
6 1 2 2	0.2583	1.2948
6 1 2 3	-0.2302	0.7944
6 1 2 4	-0.0987	0.9060

6 1 2 5	-0.0043	0.9957
6 2 1 1	0.0748	1.0777
6 2 1 2	0.2583	1.2948
6 2 1 3	-0.2302	0.7944
6 2 1 4	-0.0987	0.9060
6 2 1 5	-0.0043	0.9957
6 2 2 1	-0.0748	0.9279
6 2 2 2	-0.2583	0.7723
6 2 2 3	0.2302	1.2588
6 2 2 4	0.0987	1.1038
6 2 2 5	0.0043	1.0043
7 1 1 1	0.1141	1.1208
7 1 1 2	-0.0393	0.9614
7 1 1 3	-0.2073	0.8128
7 1 1 4	0.1357	1.1454
7 1 1 5	-0.0032	0.9968
7 1 2 1	-0.1141	0.8922
7 1 2 2	0.0393	1.0401
7 1 2 3	0.2073	1.2303
7 1 2 4	-0.1357	0.8731
7 1 2 5	0.0032	1.0032
7 2 1 1	-0.1141	0.8922
7 2 1 2	0.0393	1.0401
7 2 1 3	0.2073	1.2303
7 2 1 4	-0.1357	0.8731
7 2 1 5	0.0032	1.0032
7 2 2 1	0.1141	1.1208
7 2 2 2	-0.0393	0.9614
7 2 2 3	-0.2073	0.8128
7 2 2 4	0.1357	1.1454
7 2 2 5	-0.0032	0.9968

PCS [spe(OD,1a)]

1	1.0000
2	0.4873
3	1.0834
4	0.7466
5	1.0900
6	0.7228
7	1.1240
8	0.8212
9	0.9286
10	0.8398
11	1.2507
12	0.8085
13	0.7180
14	0.8697
15	0.7868
16	0.5347

17	0.5168
18	0.7486
19	0.7084
20	0.4134
21	0.5037
22	0.7047
23	0.6829
24	0.4088
25	0.7131
26	0.9822
27	0.7093
28	0.4786

spe(OD,1a) [PCS]

1	0.9457	2.5745
2	0.0078	1.0078
3	-0.0201	0.9801
4	-0.4542	0.6350
5	0.1829	1.2007
6	0.4252	1.5300
7	-0.4693	0.6254
8	-0.0546	0.9469
9	-0.0240	0.9763
10	-0.1991	0.8195
11	0.6390	1.8946
12	-0.1235	0.8838
13	-0.3257	0.7220
14	-0.0412	0.9596
15	-0.1810	0.8344
16	0.4324	1.5410

*** (CONDITIONAL) PROBABILITIES ***

* P(PCSOD) *

1 1 1 1 1	0.0039
1 1 1 1 2	0.0005
1 1 1 1 3	0.0009
1 1 1 1 4	0.0005
1 1 1 1 5	0.0010
1 1 1 2 1	0.0023
1 1 1 2 2	0.0010
1 1 1 2 3	0.0007
1 1 1 2 4	0.0009
1 1 1 2 5	0.0019
1 1 1 3 1	0.0067
1 1 1 3 2	0.0020

1 1 1 3 3	0.0077
1 1 1 3 4	0.0029
1 1 1 3 5	0.0053
1 1 1 4 1	0.0032
1 1 1 4 2	0.0015
1 1 1 4 3	0.0022
1 1 1 4 4	0.0032
1 1 1 4 5	0.0051
1 1 1 5 1	0.0095
1 1 1 5 2	0.0060
1 1 1 5 3	0.0127
1 1 1 5 4	0.0122
1 1 1 5 5	0.0453
1 1 2 1 1	0.0018
1 1 2 1 2	0.0009
1 1 2 1 3	0.0009
1 1 2 1 4	0.0003
1 1 2 1 5	0.0010
1 1 2 2 1	0.0014
1 1 2 2 2	0.0013
1 1 2 2 3	0.0008
1 1 2 2 4	0.0004
1 1 2 2 5	0.0013
1 1 2 3 1	0.0033
1 1 2 3 2	0.0025
1 1 2 3 3	0.0034
1 1 2 3 4	0.0010
1 1 2 3 5	0.0030
1 1 2 4 1	0.0021
1 1 2 4 2	0.0019
1 1 2 4 3	0.0017
1 1 2 4 4	0.0009
1 1 2 4 5	0.0027
1 1 2 5 1	0.0088
1 1 2 5 2	0.0095
1 1 2 5 3	0.0099
1 1 2 5 4	0.0043
1 1 2 5 5	0.0193
1 2 1 1 1	0.0032
1 2 1 1 2	0.0010
1 2 1 1 3	0.0005
1 2 1 1 4	0.0005
1 2 1 1 5	0.0009
1 2 1 2 1	0.0012
1 2 1 2 2	0.0014
1 2 1 2 3	0.0003
1 2 1 2 4	0.0007
1 2 1 2 5	0.0012

1 2 1 3 1	0.0037
1 2 1 3 2	0.0027
1 2 1 3 3	0.0036
1 2 1 3 4	0.0025
1 2 1 3 5	0.0037
1 2 1 4 1	0.0016
1 2 1 4 2	0.0019
1 2 1 4 3	0.0009
1 2 1 4 4	0.0028
1 2 1 4 5	0.0035
1 2 1 5 1	0.0035
1 2 1 5 2	0.0059
1 2 1 5 3	0.0040
1 2 1 5 4	0.0077
1 2 1 5 5	0.0244
1 2 2 1 1	0.0021
1 2 2 1 2	0.0019
1 2 2 1 3	0.0005
1 2 2 1 4	0.0002
1 2 2 1 5	0.0006
1 2 2 2 1	0.0011
1 2 2 2 2	0.0023
1 2 2 2 3	0.0003
1 2 2 2 4	0.0002
1 2 2 2 5	0.0007
1 2 2 3 1	0.0033
1 2 2 3 2	0.0053
1 2 2 3 3	0.0029
1 2 2 3 4	0.0007
1 2 2 3 5	0.0023
1 2 2 4 1	0.0009
1 2 2 4 2	0.0021
1 2 2 4 3	0.0006
1 2 2 4 4	0.0004
1 2 2 4 5	0.0011
1 2 2 5 1	0.0031
1 2 2 5 2	0.0086
1 2 2 5 3	0.0029
1 2 2 5 4	0.0014
1 2 2 5 5	0.0079
2 1 1 1 1	0.0034
2 1 1 1 2	0.0007
2 1 1 1 3	0.0007
2 1 1 1 4	0.0006
2 1 1 1 5	0.0009
2 1 1 2 1	0.0013
2 1 1 2 2	0.0009
2 1 1 2 3	0.0004

2 1 1 2 4	0.0008
2 1 1 2 5	0.0011
2 1 1 3 1	0.0015
2 1 1 3 2	0.0007
2 1 1 3 3	0.0018
2 1 1 3 4	0.0011
2 1 1 3 5	0.0014
2 1 1 4 1	0.0021
2 1 1 4 2	0.0016
2 1 1 4 3	0.0014
2 1 1 4 4	0.0038
2 1 1 4 5	0.0040
2 1 1 5 1	0.0028
2 1 1 5 2	0.0030
2 1 1 5 3	0.0039
2 1 1 5 4	0.0065
2 1 1 5 5	0.0175
2 1 2 1 1	0.0011
2 1 2 1 2	0.0005
2 1 2 1 3	0.0004
2 1 2 1 4	0.0002
2 1 2 1 5	0.0005
2 1 2 2 1	0.0007
2 1 2 2 2	0.0007
2 1 2 2 3	0.0003
2 1 2 2 4	0.0002
2 1 2 2 5	0.0007
2 1 2 3 1	0.0011
2 1 2 3 2	0.0008
2 1 2 3 3	0.0012
2 1 2 3 4	0.0004
2 1 2 3 5	0.0012
2 1 2 4 1	0.0015
2 1 2 4 2	0.0015
2 1 2 4 3	0.0011
2 1 2 4 4	0.0011
2 1 2 4 5	0.0027
2 1 2 5 1	0.0023
2 1 2 5 2	0.0029
2 1 2 5 3	0.0028
2 1 2 5 4	0.0020
2 1 2 5 5	0.0092
2 2 1 1 1	0.0025
2 2 1 1 2	0.0004
2 2 1 1 3	0.0002
2 2 1 1 4	0.0004
2 2 1 1 5	0.0004
2 2 1 2 1	0.0012

2 2 1 2 2	0.0008
2 2 1 2 3	0.0001
2 2 1 2 4	0.0007
2 2 1 2 5	0.0007
2 2 1 3 1	0.0017
2 2 1 3 2	0.0007
2 2 1 3 3	0.0010
2 2 1 3 4	0.0013
2 2 1 3 5	0.0011
2 2 1 4 1	0.0022
2 2 1 4 2	0.0015
2 2 1 4 3	0.0007
2 2 1 4 4	0.0042
2 2 1 4 5	0.0029
2 2 1 5 1	0.0020
2 2 1 5 2	0.0018
2 2 1 5 3	0.0013
2 2 1 5 4	0.0048
2 2 1 5 5	0.0089
2 2 2 1 1	0.0017
2 2 2 1 2	0.0010
2 2 2 1 3	0.0002
2 2 2 1 4	0.0001
2 2 2 1 5	0.0004
2 2 2 2 1	0.0007
2 2 2 2 2	0.0011
2 2 2 2 3	0.0001
2 2 2 2 4	0.0001
2 2 2 2 5	0.0004
2 2 2 3 1	0.0010
2 2 2 3 2	0.0011
2 2 2 3 3	0.0004
2 2 2 3 4	0.0001
2 2 2 3 5	0.0005
2 2 2 4 1	0.0012
2 2 2 4 2	0.0020
2 2 2 4 3	0.0004
2 2 2 4 4	0.0002
2 2 2 4 5	0.0011
2 2 2 5 1	0.0020
2 2 2 5 2	0.0043
2 2 2 5 3	0.0011
2 2 2 5 4	0.0005
2 2 2 5 5	0.0048
3 1 1 1 1	0.0028
3 1 1 1 2	0.0005
3 1 1 1 3	0.0008
3 1 1 1 4	0.0003

3 1 1 1 5	0.0004
3 1 1 2 1	0.0023
3 1 1 2 2	0.0012
3 1 1 2 3	0.0008
3 1 1 2 4	0.0008
3 1 1 2 5	0.0011
3 1 1 3 1	0.0021
3 1 1 3 2	0.0008
3 1 1 3 3	0.0026
3 1 1 3 4	0.0009
3 1 1 3 5	0.0010
3 1 1 4 1	0.0017
3 1 1 4 2	0.0009
3 1 1 4 3	0.0013
3 1 1 4 4	0.0016
3 1 1 4 5	0.0015
3 1 1 5 1	0.0014
3 1 1 5 2	0.0010
3 1 1 5 3	0.0020
3 1 1 5 4	0.0016
3 1 1 5 5	0.0034
3 1 2 1 1	0.0033
3 1 2 1 2	0.0010
3 1 2 1 3	0.0006
3 1 2 1 4	0.0002
3 1 2 1 5	0.0008
3 1 2 2 1	0.0022
3 1 2 2 2	0.0017
3 1 2 2 3	0.0005
3 1 2 2 4	0.0004
3 1 2 2 5	0.0014
3 1 2 3 1	0.0022
3 1 2 3 2	0.0012
3 1 2 3 3	0.0016
3 1 2 3 4	0.0004
3 1 2 3 5	0.0013
3 1 2 4 1	0.0017
3 1 2 4 2	0.0014
3 1 2 4 3	0.0008
3 1 2 4 4	0.0007
3 1 2 4 5	0.0019
3 1 2 5 1	0.0015
3 1 2 5 2	0.0016
3 1 2 5 3	0.0013
3 1 2 5 4	0.0008
3 1 2 5 5	0.0043
3 2 1 1 1	0.0013
3 2 1 1 2	0.0005

3 2 1 1 3	0.0003
3 2 1 1 4	0.0003
3 2 1 1 5	0.0003
3 2 1 2 1	0.0005
3 2 1 2 2	0.0008
3 2 1 2 3	0.0001
3 2 1 2 4	0.0005
3 2 1 2 5	0.0004
3 2 1 3 1	0.0005
3 2 1 3 2	0.0005
3 2 1 3 3	0.0007
3 2 1 3 4	0.0006
3 2 1 3 5	0.0004
3 2 1 4 1	0.0003
3 2 1 4 2	0.0006
3 2 1 4 3	0.0003
3 2 1 4 4	0.0011
3 2 1 4 5	0.0007
3 2 1 5 1	0.0003
3 2 1 5 2	0.0008
3 2 1 5 3	0.0005
3 2 1 5 4	0.0013
3 2 1 5 5	0.0023
3 2 2 1 1	0.0013
3 2 2 1 2	0.0007
3 2 2 1 3	0.0001
3 2 2 1 4	0.0002
3 2 2 1 5	0.0003
3 2 2 2 1	0.0007
3 2 2 2 2	0.0009
3 2 2 2 3	0.0001
3 2 2 2 4	0.0002
3 2 2 2 5	0.0004
3 2 2 3 1	0.0009
3 2 2 3 2	0.0009
3 2 2 3 3	0.0003
3 2 2 3 4	0.0004
3 2 2 3 5	0.0006
3 2 2 4 1	0.0007
3 2 2 4 2	0.0010
3 2 2 4 3	0.0002
3 2 2 4 4	0.0005
3 2 2 4 5	0.0008
3 2 2 5 1	0.0007
3 2 2 5 2	0.0012
3 2 2 5 3	0.0003
3 2 2 5 4	0.0006
3 2 2 5 5	0.0019

4 1 1 1 1	0.0026
4 1 1 1 2	0.0009
4 1 1 1 3	0.0007
4 1 1 1 4	0.0007
4 1 1 1 5	0.0012
4 1 1 2 1	0.0006
4 1 1 2 2	0.0005
4 1 1 2 3	0.0002
4 1 1 2 4	0.0004
4 1 1 2 5	0.0007
4 1 1 3 1	0.0021
4 1 1 3 2	0.0012
4 1 1 3 3	0.0018
4 1 1 3 4	0.0015
4 1 1 3 5	0.0022
4 1 1 4 1	0.0011
4 1 1 4 2	0.0009
4 1 1 4 3	0.0006
4 1 1 4 4	0.0014
4 1 1 4 5	0.0019
4 1 1 5 1	0.0013
4 1 1 5 2	0.0013
4 1 1 5 3	0.0012
4 1 1 5 4	0.0020
4 1 1 5 5	0.0049
4 1 2 1 1	0.0019
4 1 2 1 2	0.0018
4 1 2 1 3	0.0004
4 1 2 1 4	0.0003
4 1 2 1 5	0.0008
4 1 2 2 1	0.0003
4 1 2 2 2	0.0008
4 1 2 2 3	0.0001
4 1 2 2 4	0.0001
4 1 2 2 5	0.0004
4 1 2 3 1	0.0010
4 1 2 3 2	0.0017
4 1 2 3 3	0.0008
4 1 2 3 4	0.0005
4 1 2 3 5	0.0011
4 1 2 4 1	0.0006
4 1 2 4 2	0.0015
4 1 2 4 3	0.0003
4 1 2 4 4	0.0006
4 1 2 4 5	0.0012
4 1 2 5 1	0.0008
4 1 2 5 2	0.0026
4 1 2 5 3	0.0007

4 1 2 5 4	0.0010
4 1 2 5 5	0.0042
4 2 1 1 1	0.0014
4 2 1 1 2	0.0008
4 2 1 1 3	0.0002
4 2 1 1 4	0.0005
4 2 1 1 5	0.0006
4 2 1 2 1	0.0003
4 2 1 2 2	0.0003
4 2 1 2 3	0.0000
4 2 1 2 4	0.0002
4 2 1 2 5	0.0003
4 2 1 3 1	0.0004
4 2 1 3 2	0.0004
4 2 1 3 3	0.0002
4 2 1 3 4	0.0004
4 2 1 3 5	0.0004
4 2 1 4 1	0.0004
4 2 1 4 2	0.0006
4 2 1 4 3	0.0001
4 2 1 4 4	0.0008
4 2 1 4 5	0.0008
4 2 1 5 1	0.0004
4 2 1 5 2	0.0006
4 2 1 5 3	0.0002
4 2 1 5 4	0.0009
4 2 1 5 5	0.0016
4 2 2 1 1	0.0016
4 2 2 1 2	0.0016
4 2 2 1 3	0.0001
4 2 2 1 4	0.0002
4 2 2 1 5	0.0003
4 2 2 2 1	0.0004
4 2 2 2 2	0.0007
4 2 2 2 3	0.0000
4 2 2 2 4	0.0001
4 2 2 2 5	0.0001
4 2 2 3 1	0.0006
4 2 2 3 2	0.0009
4 2 2 3 3	0.0001
4 2 2 3 4	0.0002
4 2 2 3 5	0.0002
4 2 2 4 1	0.0003
4 2 2 4 2	0.0007
4 2 2 4 3	0.0000
4 2 2 4 4	0.0002
4 2 2 4 5	0.0002
4 2 2 5 1	0.0008

4 2 2 5 2	0.0017
4 2 2 5 3	0.0001
4 2 2 5 4	0.0004
4 2 2 5 5	0.0007
5 1 1 1 1	0.0049
5 1 1 1 2	0.0006
5 1 1 1 3	0.0007
5 1 1 1 4	0.0013
5 1 1 1 5	0.0011
5 1 1 2 1	0.0004
5 1 1 2 2	0.0001
5 1 1 2 3	0.0001
5 1 1 2 4	0.0002
5 1 1 2 5	0.0002
5 1 1 3 1	0.0021
5 1 1 3 2	0.0003
5 1 1 3 3	0.0007
5 1 1 3 4	0.0011
5 1 1 3 5	0.0008
5 1 1 4 1	0.0029
5 1 1 4 2	0.0006
5 1 1 4 3	0.0007
5 1 1 4 4	0.0023
5 1 1 4 5	0.0017
5 1 1 5 1	0.0015
5 1 1 5 2	0.0004
5 1 1 5 3	0.0005
5 1 1 5 4	0.0013
5 1 1 5 5	0.0015
5 1 2 1 1	0.0059
5 1 2 1 2	0.0033
5 1 2 1 3	0.0003
5 1 2 1 4	0.0001
5 1 2 1 5	0.0004
5 1 2 2 1	0.0005
5 1 2 2 2	0.0006
5 1 2 2 3	0.0000
5 1 2 2 4	0.0000
5 1 2 2 5	0.0001
5 1 2 3 1	0.0021
5 1 2 3 2	0.0021
5 1 2 3 3	0.0004
5 1 2 3 4	0.0001
5 1 2 3 5	0.0004
5 1 2 4 1	0.0028
5 1 2 4 2	0.0038
5 1 2 4 3	0.0003
5 1 2 4 4	0.0003

5 1 2 4 5	0.0008
5 1 2 5 1	0.0015
5 1 2 5 2	0.0026
5 1 2 5 3	0.0003
5 1 2 5 4	0.0002
5 1 2 5 5	0.0010
5 2 1 1 1	0.0018
5 2 1 1 2	0.0009
5 2 1 1 3	0.0000
5 2 1 1 4	0.0005
5 2 1 1 5	0.0005
5 2 1 2 1	0.0001
5 2 1 2 2	0.0001
5 2 1 2 3	0.0000
5 2 1 2 4	0.0001
5 2 1 2 5	0.0001
5 2 1 3 1	0.0002
5 2 1 3 2	0.0002
5 2 1 3 3	0.0000
5 2 1 3 4	0.0001
5 2 1 3 5	0.0001
5 2 1 4 1	0.0005
5 2 1 4 2	0.0006
5 2 1 4 3	0.0000
5 2 1 4 4	0.0006
5 2 1 4 5	0.0005
5 2 1 5 1	0.0002
5 2 1 5 2	0.0003
5 2 1 5 3	0.0000
5 2 1 5 4	0.0003
5 2 1 5 5	0.0005
5 2 2 1 1	0.0013
5 2 2 1 2	0.0014
5 2 2 1 3	0.0001
5 2 2 1 4	0.0001
5 2 2 1 5	0.0003
5 2 2 2 1	0.0002
5 2 2 2 2	0.0003
5 2 2 2 3	0.0000
5 2 2 2 4	0.0000
5 2 2 2 5	0.0001
5 2 2 3 1	0.0002
5 2 2 3 2	0.0003
5 2 2 3 3	0.0000
5 2 2 3 4	0.0000
5 2 2 3 5	0.0001
5 2 2 4 1	0.0006
5 2 2 4 2	0.0011

5 2 2 4 3	0.0000
5 2 2 4 4	0.0001
5 2 2 4 5	0.0003
5 2 2 5 1	0.0005
5 2 2 5 2	0.0010
5 2 2 5 3	0.0001
5 2 2 5 4	0.0001
5 2 2 5 5	0.0004
6 1 1 1 1	0.0049
6 1 1 1 2	0.0006
6 1 1 1 3	0.0007
6 1 1 1 4	0.0014
6 1 1 1 5	0.0010
6 1 1 2 1	0.0004
6 1 1 2 2	0.0001
6 1 1 2 3	0.0001
6 1 1 2 4	0.0002
6 1 1 2 5	0.0002
6 1 1 3 1	0.0019
6 1 1 3 2	0.0003
6 1 1 3 3	0.0006
6 1 1 3 4	0.0011
6 1 1 3 5	0.0007
6 1 1 4 1	0.0029
6 1 1 4 2	0.0006
6 1 1 4 3	0.0007
6 1 1 4 4	0.0024
6 1 1 4 5	0.0015
6 1 1 5 1	0.0014
6 1 1 5 2	0.0004
6 1 1 5 3	0.0005
6 1 1 5 4	0.0013
6 1 1 5 5	0.0013
6 1 2 1 1	0.0051
6 1 2 1 2	0.0031
6 1 2 1 3	0.0004
6 1 2 1 4	0.0001
6 1 2 1 5	0.0005
6 1 2 2 1	0.0004
6 1 2 2 2	0.0006
6 1 2 2 3	0.0000
6 1 2 2 4	0.0000
6 1 2 2 5	0.0001
6 1 2 3 1	0.0018
6 1 2 3 2	0.0020
6 1 2 3 3	0.0004
6 1 2 3 4	0.0001
6 1 2 3 5	0.0004

6 1 2 4 1	0.0026
6 1 2 4 2	0.0039
6 1 2 4 3	0.0004
6 1 2 4 4	0.0003
6 1 2 4 5	0.0009
6 1 2 5 1	0.0014
6 1 2 5 2	0.0025
6 1 2 5 3	0.0003
6 1 2 5 4	0.0002
6 1 2 5 5	0.0010
6 2 1 1 1	0.0020
6 2 1 1 2	0.0010
6 2 1 1 3	0.0000
6 2 1 1 4	0.0006
6 2 1 1 5	0.0006
6 2 1 2 1	0.0001
6 2 1 2 2	0.0001
6 2 1 2 3	0.0000
6 2 1 2 4	0.0001
6 2 1 2 5	0.0001
6 2 1 3 1	0.0002
6 2 1 3 2	0.0002
6 2 1 3 3	0.0000
6 2 1 3 4	0.0001
6 2 1 3 5	0.0001
6 2 1 4 1	0.0005
6 2 1 4 2	0.0006
6 2 1 4 3	0.0000
6 2 1 4 4	0.0006
6 2 1 4 5	0.0005
6 2 1 5 1	0.0002
6 2 1 5 2	0.0003
6 2 1 5 3	0.0000
6 2 1 5 4	0.0003
6 2 1 5 5	0.0005
6 2 2 1 1	0.0014
6 2 2 1 2	0.0014
6 2 2 1 3	0.0001
6 2 2 1 4	0.0001
6 2 2 1 5	0.0003
6 2 2 2 1	0.0002
6 2 2 2 2	0.0003
6 2 2 2 3	0.0000
6 2 2 2 4	0.0000
6 2 2 2 5	0.0001
6 2 2 3 1	0.0002
6 2 2 3 2	0.0003
6 2 2 3 3	0.0000

6 2 2 3 4	0.0000
6 2 2 3 5	0.0001
6 2 2 4 1	0.0006
6 2 2 4 2	0.0010
6 2 2 4 3	0.0000
6 2 2 4 4	0.0001
6 2 2 4 5	0.0003
6 2 2 5 1	0.0005
6 2 2 5 2	0.0010
6 2 2 5 3	0.0001
6 2 2 5 4	0.0001
6 2 2 5 5	0.0004
7 1 1 1 1	0.0090
7 1 1 1 2	0.0009
7 1 1 1 3	0.0006
7 1 1 1 4	0.0018
7 1 1 1 5	0.0012
7 1 1 2 1	0.0018
7 1 1 2 2	0.0004
7 1 1 2 3	0.0002
7 1 1 2 4	0.0009
7 1 1 2 5	0.0006
7 1 1 3 1	0.0011
7 1 1 3 2	0.0002
7 1 1 3 3	0.0003
7 1 1 3 4	0.0006
7 1 1 3 5	0.0003
7 1 1 4 1	0.0068
7 1 1 4 2	0.0017
7 1 1 4 3	0.0011
7 1 1 4 4	0.0065
7 1 1 4 5	0.0035
7 1 1 5 1	0.0035
7 1 1 5 2	0.0011
7 1 1 5 3	0.0009
7 1 1 5 4	0.0039
7 1 1 5 5	0.0038
7 1 2 1 1	0.0074
7 1 2 1 2	0.0043
7 1 2 1 3	0.0005
7 1 2 1 4	0.0004
7 1 2 1 5	0.0009
7 1 2 2 1	0.0011
7 1 2 2 2	0.0020
7 1 2 2 3	0.0001
7 1 2 2 4	0.0002
7 1 2 2 5	0.0004
7 1 2 3 1	0.0005

7 1 2 3 2	0.0006
7 1 2 3 3	0.0002
7 1 2 3 4	0.0001
7 1 2 3 5	0.0002
7 1 2 4 1	0.0047
7 1 2 4 2	0.0091
7 1 2 4 3	0.0010
7 1 2 4 4	0.0019
7 1 2 4 5	0.0037
7 1 2 5 1	0.0021
7 1 2 5 2	0.0054
7 1 2 5 3	0.0008
7 1 2 5 4	0.0010
7 1 2 5 5	0.0047
7 2 1 1 1	0.0023
7 2 1 1 2	0.0007
7 2 1 1 3	0.0001
7 2 1 1 4	0.0007
7 2 1 1 5	0.0005
7 2 1 2 1	0.0004
7 2 1 2 2	0.0003
7 2 1 2 3	0.0000
7 2 1 2 4	0.0003
7 2 1 2 5	0.0002
7 2 1 3 1	0.0002
7 2 1 3 2	0.0001
7 2 1 3 3	0.0000
7 2 1 3 4	0.0001
7 2 1 3 5	0.0001
7 2 1 4 1	0.0013
7 2 1 4 2	0.0010
7 2 1 4 3	0.0002
7 2 1 4 4	0.0020
7 2 1 4 5	0.0011
7 2 1 5 1	0.0005
7 2 1 5 2	0.0004
7 2 1 5 3	0.0001
7 2 1 5 4	0.0008
7 2 1 5 5	0.0008
7 2 2 1 1	0.0023
7 2 2 1 2	0.0017
7 2 2 1 3	0.0001
7 2 2 1 4	0.0002
7 2 2 1 5	0.0004
7 2 2 2 1	0.0004
7 2 2 2 2	0.0006
7 2 2 2 3	0.0000
7 2 2 2 4	0.0001

7 2 2 2 5	0.0001
7 2 2 3 1	0.0001
7 2 2 3 2	0.0001
7 2 2 3 3	0.0000
7 2 2 3 4	0.0000
7 2 2 3 5	0.0000
7 2 2 4 1	0.0018
7 2 2 4 2	0.0024
7 2 2 4 3	0.0001
7 2 2 4 4	0.0005
7 2 2 4 5	0.0007
7 2 2 5 1	0.0009
7 2 2 5 2	0.0015
7 2 2 5 3	0.0001
7 2 2 5 4	0.0003
7 2 2 5 5	0.0006

For any clarification or extra data, do not hesitate to contact me. César Augusto Ricardi Morgavi, Department of Social and Legal Science, CUCEA, University of Guadalajara.
cesar.ricardi@cucea.udg.mx
personal email: sociologicalthinktankblog@gmail.com

Cite this data as: Ricardi-Morgavi, C. A. (2026). Comparing Patterns of Intergenerational Class Mobility Using Log-Linear Models: Evidence from Seven Countries, Two Cohorts, and Gendered Stratification. Frontiers special issue.
