# Supplementary material for: Comparing patterns of intergenerational class mobility using log-linear models: evidence from seven countries, two cohorts, and gendered stratification
Source: Front Sociol. 2026 May 1;11:1757240. doi: 10.3389/fsoc.2026.1757240 (PMC13177305; doi:10.3389/fsoc.2026.1757240)
Supplement: Supplementary file 4 [file Data_Sheet_4.pdf]

```
  man 4
  dim 7 2 5 5
  lab P C O D
  *  mod {PCO PCD OD}
  *  mod {PCO PCD POD}
  *  mod {PCO PCD spe(OD,1a,P,b)}
  *  mod {PCO PCD COD}
  *  mod {PCO PCD spe(OD,1a,C,b)}
  *  mod {PCO PCD POD COD}
    mod {PCO PCD spe(OD,1a,PC,b)}
add .05
nse

  dat[101	11	19	17	44
62	25	15	28	70
180	76	185	70	116
85	60	67	62	131
285	279	390	265	1066
96	57	19	14	5
21	75	14	9	39
115	152	88	51	103
21	72	21	69	78
143	195	132	141	542
75	15	25	1	31
30	27	11	20	28
43	31	46	40	26
60	46	45	77	119
85	104	103	137	448
69	21	14	10	6
19	36	2	18	23
46	33	17	17	34
67	53	27	77	47
66	101	30	84	241
96	32	24	9	16
91	43	27	15	30
95	35	52	16	37
34	30	43	38	81
37	48	58	50	120
44	21	4	11	6
28	29	6	9	6
26	21	10	16	22
8	29	8	20	34
11	30	19	38	66
70	54	16	13	33
23	17	7	8	14
44	43	48	37	61
29	45	14	33	45
36	60	29	51	155
54	34	4	12	17
13	14	1	5	9
10	22	8	7	12
11	26	1	13	15
18	41	5	27	32
181	61	15	20	32
11	13	1	4	6
59	38	28	17	25
110	69	13	48	31
46	56	10	29	36
52	39	0	10	13
5	9	0	1	1
5	4	3	5	4
19	22	0	12	19
11	27	1	5	10
166	58	14	20	34
```

8	14	1	4	6
55	36	26	18	20
107	69	14	49	31
42	55	12	28	32
57	40	0	11	16
5	10	0	1	1
6	4	3	5	4
20	22	0	12	18
12	28	1	5	11
258	85	29	35	44
49	42	0	16	19
18	0	37	9	2
212	191	19	128	112
95	112	8	96	142
71	43	1	21	13
13	13	0	8	6
3	0	8	0	0
56	60	1	40	26
25	29	0	17	29

]

*Order of the countries: Mexico, Chile, Uruguay, Spain, Sweden, UK and Germany.
*Order of the cohorts: old 1930-1975; youth 1976-1990.

For any clarification or extra data, do not hesitate to contact me. César Augusto Ricardi Morgavi, Department of Social and Legal Science, CUCEA, University of Guadalajara. cesar.ricardi@cucea.udg.mx personal email: sociologicalthinktankblog@gmail.com

Cite this data as: Ricardi-Morgavi, C. A. (2026). Comparing Patterns of Intergenerational Class Mobility Using Log-Linear Models: Evidence from Seven Countries, Two Cohorts, and Gendered Stratification. Frontiers special issue.
