# Supplementary material for: Comparing patterns of intergenerational class mobility using log-linear models: evidence from seven countries, two cohorts, and gendered stratification
Source: Front Sociol. 2026 May 1;11:1757240. doi: 10.3389/fsoc.2026.1757240 (PMC13177305; doi:10.3389/fsoc.2026.1757240)
Supplement: Supplementary file 5 [file Data_Sheet_5.pdf]

```
man 4
dim 7 2 5 5
lab P S O D
*  mod {PSO PSD OD}
*  mod {PSO PSD POD}
*  mod {PSO PSD spe(OD,1a,P,b)}
*  mod {PSO PSD SOD}
*  mod {PSO PSD spe(OD,1a,S,b)}
*  mod {PSO PSD POD SOD}
  mod {PSO PSD spe(OD,1a,PS,b)}
add .05
nse
dat[131 24      11      28      21
58      36      18      30      51
166     82      181     85      163
68      51      51      117     141
223     204     293     304     1156
66      44      27      3       28
25      64      11      7       58
129     146     92      36      56
38      81      37      14      68
205     270     229     102     452
97      20      14      11      28
35      33      10      31      24
49      20      48      43      44
78      48      46      136     97
83      82      72      180     453
47      16      25      0       9
14      30      3       7       27
40      44      15      14      16
49      51      26      18      69
68      123     61      41      236
69      16      16      11      11
50      40      19      17      16
62      19      42      18      27
18      22      31      43      52
22      28      49      60      85
71      37      12      9       11
69      32      14      7       20
59      37      20      14      32
24      37      20      15      63
26      50      28      28      101
63      26      13      19      36
19      11      5       9       16
31      22      45      31      47
30      38      9       32      32
29      27      17      55      111
61      62      7       6       14
17      20      3       4       7
23      43      11      13      26
10      33      6       14      28
25      74      17      23      76
108     27      11      27      30
6       7       0       4       5
27      6       23      19      19
69      16      7       53      27
31      10      5       28      32
125     73      4       3       15
10      15      1       1       2
37      36      8       3       10
60      75      6       7       23
26      73      6       6       14
110     28      10      28      33
5       8       0       4       5
```

26	6	21	20	15
70	17	6	54	25
30	10	5	27	30
113	70	4	3	17
8	16	1	1	2
35	34	8	3	9
57	74	8	7	24
24	73	8	6	13
174	31	21	39	34
34	17	0	19	14
17	0	24	6	2
152	41	10	142	71
71	25	2	86	80
155	97	9	17	23
28	38	0	5	11
4	0	21	3	0
116	210	10	26	67
49	116	6	27	91]

*Order of the countries: Mexico, Chile, Uruguay, Spain, Sweden, UK and Germany.
*Order of gender: Male, female.

For any clarification or extra data, do not hesitate to contact me. César Augusto Ricardi Morgavi, Department of Social and Legal Science, CUCEA, University of Guadalajara. cesar.ricardi@cucea.udg.mx personal email: sociologicalthinktankblog@gmail.com

Cite this data as: Ricardi-Morgavi, C. A. (2026). Comparing Patterns of Intergenerational Class Mobility Using Log-Linear Models: Evidence from Seven Countries, Two Cohorts, and Gendered Stratification. Frontiers special issue.
