# Supplementary material for: Comparing patterns of intergenerational class mobility using log-linear models: evidence from seven countries, two cohorts, and gendered stratification
Source: Front Sociol. 2026 May 1;11:1757240. doi: 10.3389/fsoc.2026.1757240 (PMC13177305; doi:10.3389/fsoc.2026.1757240)
Supplement: Supplementary file 6 [file Data_Sheet_6.pdf]

```
man 5
dim 7 2 2 5 5
lab P C S O D
* mod {PCSO PCSD OD}
* mod {PCSO PCSD PCOD PSOD CSOD}
 mod {PCSO PCSD spe(OD,1a,PCS,b)}
add .05
nse

dat[71  2       7       15      17
40      13      7       22      31
114     40      120     48      86
51      16      34      60      90
149     111     232     183     749
30      9       12      2       27
22      12      8       6       39
66      36      65      22      30
34      44      33      2       41
136     168     158     82      317
60      22      4       13      4
18      23      11      8       20
52      42      61      37      77
17      35      17      57      51
74      93      61      121     407
36      35      15      1       1
3       52      3       1       19
63      110     27      14      26
4       37      4       12      27
69      102     71      20      135
58      11      12      1       23
22      14      8       14      16
15      15      37      27      15
37      26      23      62      67
52      51      56      107     293
17      4       13      0       8
8       13      3       6       12
28      16      9       13      11
23      20      22      15      52
33      53      47      30      155
39      9       2       10      5
13      19      2       17      8
34      5       11      16      29
41      22      23      74      30
31      31      16      73      160
30      12      12      0       1
6       17      0       1       15
12      28      6       1       5
26      31      4       3       17
35      70      14      11      81
46      9       13      5       7
38      23      16      12      14
53      11      33      9       17
16      12      28      28      34
19      18      35      33      52
50      23      11      4       9
53      20      11      3       16
42      24      19      7       20
18      18      15      10      47
18      30      23      17      68
23      7       3       6       4
12      17      3       5       2
9       8       9       9       10
2       10      3       15      18
3       10      14      27      33
```

21	14	1	5	2
16	12	3	4	4
17	13	1	7	12
6	19	5	5	16
8	20	5	11	33
40	16	11	9	24
14	6	5	7	9
26	16	39	27	40
23	23	8	22	22
24	19	14	35	86
30	38	5	4	9
9	11	2	1	5
18	27	9	10	21
6	22	6	11	23
12	41	15	16	69
23	10	2	10	12
5	5	0	2	7
5	6	6	4	7
7	15	1	10	10
5	8	3	20	25
31	24	2	2	5
8	9	1	3	2
5	16	2	3	5
4	11	0	3	5
13	33	2	7	7
78	12	11	18	23
3	5	0	3	4
24	5	22	16	16
62	6	7	43	18
27	4	5	24	25
103	49	4	2	9
8	8	1	1	2
35	33	6	1	9
48	63	6	5	13
19	52	5	5	11
30	15	0	9	7
3	2	0	1	1
3	1	1	3	3
7	10	0	10	9
4	6	0	4	7
22	24	0	1	6
2	7	0	0	0
2	3	2	2	1
12	12	0	2	10
7	21	1	1	3
77	12	10	18	23
2	6	0	3	4
23	5	20	17	12
63	6	6	44	16
26	4	5	23	22
89	46	4	2	11
6	8	1	1	2
32	31	6	1	8
44	63	8	5	15
16	51	7	5	10
33	16	0	10	10
3	2	0	1	1
3	1	1	3	3
7	11	0	10	9
4	6	0	4	8
24	24	0	1	6
2	8	0	0	0
3	3	2	2	1
13	11	0	2	9
8	22	1	1	3

139	17	20	23	28
30	12	0	13	9
14	0	19	6	2
126	27	9	108	54
62	17	2	76	63
119	68	9	12	16
19	30	0	3	10
4	0	18	3	0
86	164	10	20	58
33	95	6	20	79
35	14	1	16	6
4	5	0	6	5
3	0	5	0	0
26	14	1	34	17
9	8	0	10	17
36	29	0	5	7
9	8	0	2	1
0	0	3	0	0
30	46	0	6	9
16	21	0	7	12

]

*Order of the countries: Mexico, Chile, Uruguay, Spain, Sweden, UK and Germany.
*Order of the cohorts: old 1930-1975; youth 1976-1990.
*Order of gender: Male, female.

For any clarification or extra data, do not hesitate to contact me. César Augusto Ricardi Morgavi, Department of Social and Legal Science, CUCEA, University of Guadalajara. cesar.ricardi@cucea.udg.mx
personal email: sociologicalthinktankblog@gmail.com

Cite this data as: Ricardi-Morgavi, C. A. (2026). Comparing Patterns of Intergenerational Class Mobility Using Log-Linear Models: Evidence from Seven Countries, Two Cohorts, and Gendered Stratification. Frontiers special issue.
